# Continuous electricity from charged total dissolved solids in wastewater using a wood-based ion-selective power generator

**DOI:** 10.1038/s41467-026-75514-7

**Published:** 2026-07-09

**Authors:** Wenqing Yan, Jianguo Sun, Muze Han, Jonas Garemark, Maria F. Cortes Ruiz, Federico M. Orlando, Sophie M. Koch, Liyuan Bao, Evamaria Fuchs, Özen Eroğlu, Christopher Hubert Dreimol, Yong Ding, Feng Chen, Lavinia Heisenberg, Shihang Li, Kunkun Tu

**Affiliations:** 1 Wood Materials Science, Institute for Building Materials, ETH Zürich, Zürich, Switzerland; 2 Jiangsu Key Laboratory of Coal–based Greenhouse Gas Control and Uftilization, Carbon Neutrality Institute, China University of Mining and Technology, Xuzhou, China; 3 School of Environment Science and Spatial Informatics, China, University of Mining and Technology, Xuzhou, China; 4 Wallenberg Wood Science Center, Department of Fiber and Polymer Technology, KTH Royal Institute of Technology, Stockholm, Sweden; 5 The Durability of Engineering Materials Group, Institute for Building Materials, ETH Zürich, Zürich, Switzerland; 6 WoodTec Group, Cellulose & Wood Materials, Empa, Dübendorf, Switzerland; 7 Department of Natural Sciences and Sustainable Resources, Institute of Wood Technology and Renewable Materials, BOKU University, Konrad Lorenz-Straße 24, Tulln/Donau, Austria; 8 School of Digital Technology and Innovation Design, Jiangnan University, No.1800 Lihu Boulevard, Binhu District, Wuxi City, Jiangsu Province China; 9 Key Laboratory of Optoelectronic Chemical Materials and Devices, Ministry of 7 Education, Jianghan University, Wuhan, China; 10 Institute for Theoretical Physics, ETH Zürich, Wolfgang-Pauli-Strasse 27, Zürich, Switzerland; 11 Institute for Theoretical Physics, Heidelberg University, Philosophenweg 16, Heidelberg, Germany

**Keywords:** Pollution remediation, Structural materials

## Abstract

Exploring the potential for secondary utilization of wastewater is a prudent strategy to achieve “take-make-use-reuse” circular economy. Taking advantages of wood’s hierarchical structure and large surface area, in this project, we fabricate surface-encapsulated anion-selective and cation-selective wood membranes (comprising up to 98% eco-friendly materials) through a two-step process: dip-coating with either positively charged 2(dimethylamino)ethyl methacrylate or negatively charged acrylic acid, followed by energy-efficient sunlight-induced polymerization. The output voltage and current of a single modified wood cell (20 × 20 × 3 mm^3^) in modulated wastewater from flue gas desulfurization are 55 mV and 0.6 µA, respectively, tenfold higher than that of untreated wood cells. When five cells are connected in series, the output voltage reaches 0.27 V, sufficient to power simple electronic devices. This underscores its potential for scaling up and its viability for future applications in industrial power plants.

## Abstract (web summary)

Wood-based ion-selective membranes fabricated via sunlight-induced polymerization harvest osmotic energy from industrial wastewater, achieving tenfold higher output than untreated wood and powering electronic devices when connected in series.

## Introduction

More than 80% of the world’s wastewater is released into waterways without any treatment, resulting in contamination of lakes, rivers, oceans, and the potential for environmental challenges^1^. One key reason for wastewater treatment complexity is the existence of charged total dissolved solids, encompassing ions (e.g., Na⁺, K⁺, Ca^2^⁺, Cl⁻, SO_4_^2^⁻, NO_3_⁻) and heavy metal ions (e.g., Pb^2^⁺, Cd^2^⁺, Cu^2^⁺)^2^. They can aggregate into colloidal particles due to electrostatic interactions, rendering their separation challenging^3^. Heavy metals and specific organic compounds exhibit considerable toxicity even at low concentrations^4^. Certain treatment techniques are both energy-intensive and costly^5^, often contributing to the emission of greenhouse gases^6^.

To address these challenges, a shift in perspective is needed. Instead of directly releasing wastewater into the environment, a better approach involves finding secondary uses for it. This means moving away from the traditional linear “take-make-use-dispose” model and adopting the sustainable “take-make-use-reuse” approach, known as the circular economy^7,8^. Extensive efforts have been made to realize the conversion of ionic gradients (caused by charged substances) into usable electricity without external energy input, with relevant technologies including pressure-retarded osmosis (PRO)^9^, capacitor deionization (CDI)^10,11^, reverse electrodialysis (RED)^12,13^, and biomimetic nanofluidic channel membranes^14^. This innovative utilization of the natural osmosis phenomenon has the potential to transform wastewater treatment into a resource-recovery opportunity. The design of ion-selective membranes, encompassing key parameters like pore size distribution, channel connectivity, and surface charge density, is crucial for realizing efficient osmotic power conversion through reverse electrodialysis, as they directly determine ion sieving efficiency and mass transport performance. Existing osmotic energy-harvesting membranes can be categorized into two main types: synthetic material-based membranes (e.g., polymers, two-dimensional materials)^15–20^ and bio-based membranes (e.g., bacterial cellulose, chitosan)^21,22^. Though widely studied for ion-transport capabilities, both synthetic material-based and bio-based osmotic energy-harvesting membranes face critical limitations: they not only require time- and energy-intensive preparative routes for structured selective ion channels and offer limited control over microstructure, but also suffer from poor aqueous stability. Beyond these shared drawbacks, synthetic membranes, despite tunable ion selectivity, are further constrained by non-biodegradability and high fabrication costs; bio-based membranes, while aligning with circular economy principles via biodegradability, lack mechanical strength, which compromises long-term ion-transport reliability.

Wood, a carbon-storing material, has progressively emerged as a long-term green substrate due to its intrinsic sustainability, lightweight, and remarkable mechanical properties. The primary components of wood, cellulose, hemicellulose, and lignin, along with its inherent monolithic hierarchical structure, have attracted research attention to exploit these properties for fabricating bio-based power generators by utilizing ionized wood membranes through molecular modification^23^. This process involves multiple chemical modification steps and ueses toxic reactants as well as organic catalyst, raising environmental and energy consuming concerns. Due to wood’s inherent structural characteristics, 1 mm-thick wood membranes are common preferred for efficient infiltration and high flow rates^24^. Previous studies relied on lumen-filling approaches (e.g., epoxy resin), requiring massive polymer to occupy the lumen cavities^25,26^, and thus causing unsustainable materials waste.

To address this, we herein leverage wood’s inherent hierarchical structure and relatively high specific surface area to focus on cell wall surface modification, avoid lumen filling. We dip-coated the wood in a solution containing polyelectrolyte monomers (positively charged 2(dimethylamino)ethyl methacrylate or negatively charged acrylic acid), with polymerization induced by natural sunlight or simulated UV light, thereby minimizing polymer usage. This straightforward yet highly efficient polymerization process facilitates energy-saving and scalable large-scale production. This design preserves wood’s inherent sustainability, in line with the eco-friendly principle of the circular economy. The resulting nanostructured polyelectrolyte-incorporated wood membrane acts as ion-selective channels, enabling efficient cation or anion selectivity. It establishes a potential difference inside the wood along the tracheid orientation, thereby converting the selective transport of ions through ion-exchange membranes into electrical energy without the need for external pressure or electric fields. Through this strategy, eco-friendly and cost-effective surface-encapsulated anion-selective (S-AWM) and cation-selective (S-CWM) wood membranes can selectively transport total dissolved inorganic solids from effluents, enabling green osmotic energy generation through the salinity gradient between the effluents and clean water. In comparison to untreated wood, the electrical performance increases by up to tenfold. The individual output voltage and current of a single cell (R × T × L: 20 × 20 × 3 mm^3^) in modulated wastewater from flue gas desulfurization are 55 mV and 0.6 µA, respectively. When five cells are connected in series, the output voltage reaches 0.27 V, indicating that enhancing electrical performance is achievable by integrating more cells using various electrical connection modes, tailored to specific applications. This approach offers a practical and viable design strategy for implementing power plants that contribute to the synergy between urban water systems and energy cycles.

### Membrane characterization and sustainable design

**Fig. 1:**
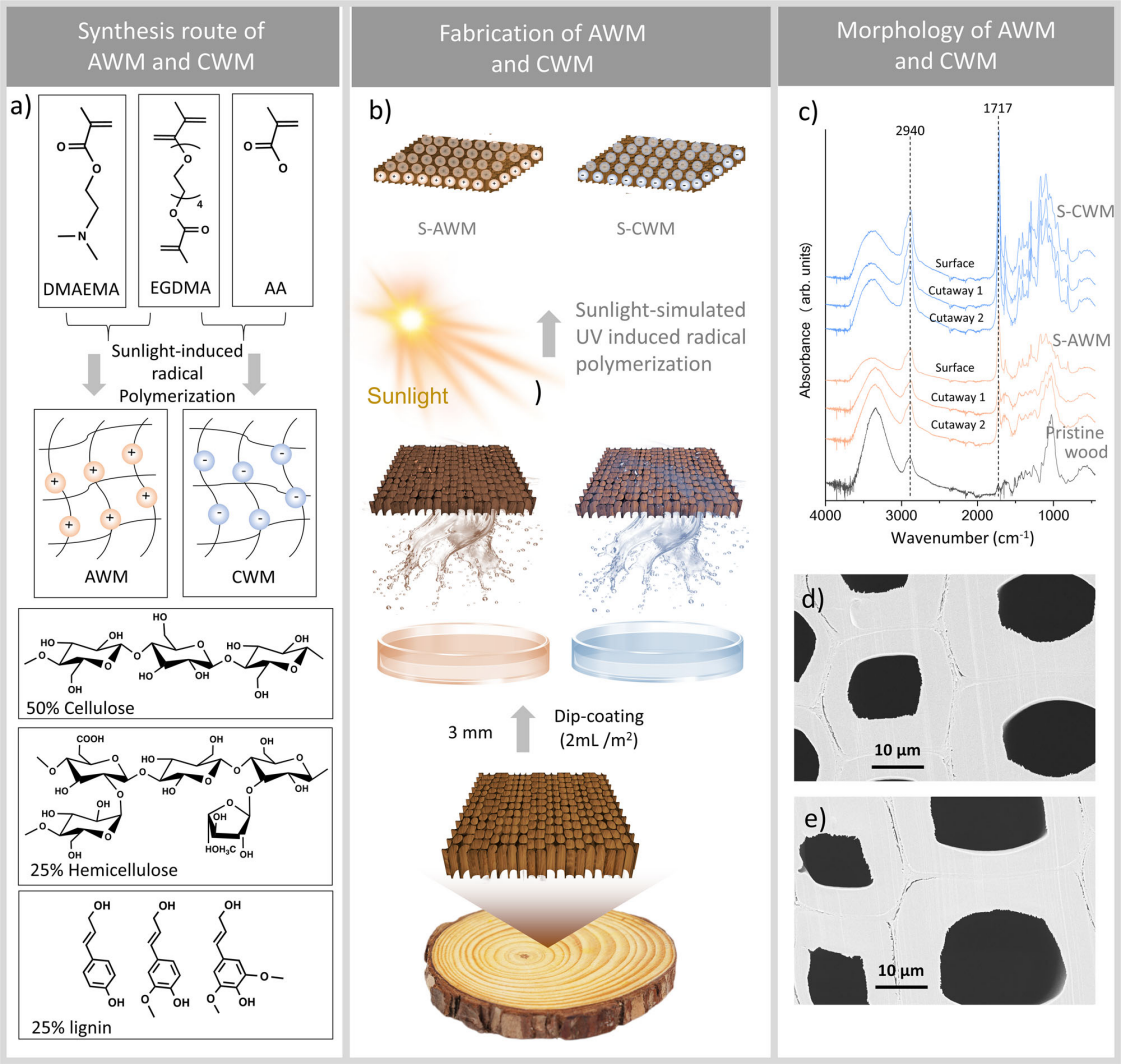
Fabrication and structural characterization of surface-encapsulated polyelectrolyte wood membranes. **a** Chemical structures of 2-(dimethylamino)ethyl methacrylate (DMAEMA), ethylene glycol dimethacrylate (EGDMA), acrylic acid (AA), wood components (50% cellulose, 25% hemicellulose, 25% lignin), and the synthesis route of anion-selective wood membrane (AWM) and cation-selective wood membrane (CWM) via sunlight-induced radical polymerization. **b** Fabrication process schematic (read from bottom to top): In situ polymerization of positively charged polyelectrolyte and negatively charged polyelectrolyte into wood after dip coating for preparing surface-encapsulated anion-selective wood membrane (S-AWM) and surface-encapsulated cation-selective wood membrane (S-CWM). **c** Fourier-transform infrared spectroscopy (FTIR) spectra of pristine wood, surface and two interior cutaways of S-AWM and S-CWM. **d**, **e** Scanning electron microscopy (SEM) images of cross sections: d) Morphology of S-AWM and e) Morphology of pristine wood. Representative SEM images from three independent experiments with similar results.

To develop ion-selective wood membranes with both environmental friendliness and practicality, we fabricated such membranes via a green and prompt sunlight-simulated UV-induced radical polymerization of monomers bearing electrolyte groups (2(dimethylamino)ethyl methacrylate (DMAEMA) for S-AWM and acrylic acid (AA) for S-CWM) (Fig. 1a, b). The radical polymerization mechanism and reaction details are clarified as follows: Under sunlight-simulated UV irradiation, monomers (DMAEMA/AA) generate radicals via photodissociation, H-abstraction, or oxygen initiation, enabling self-initiated radical polymerization without external initiators. These radicals drive chain growth, forming the electrolyte group-functionalized ion-selective wood membranes^27^. The synthetic protocol demonstrates a straightforward, cost-effective, and energy-efficient technique, as evidenced by the following points: (1) High bio-based content: A remarkable 98% of the materials used are derived from wood, making the final device highly bio-based. (2) Minimal solvent consumption: The use of monomer solutions is highly efficient, as they can be reused, and the dip-coating process consumes only 0.001 litres of solution per wood cell (R × T × L: 20 × 20 × 3 mm^3^). (3) Energy-Efficient Polymerization: The polymerization process can be initiated using readily available sunlight, contributing to its energy efficiency. The green substrate is designed based on the internal characteristic structure of wood with a thickness higher than 3 mm, which has less open tracheid features compared to a thinner one (Supplementary Fig. S1)^28^. The connection between tracheids is dependent on abundant bordered pit, with a pit aperture diameter around 1.8~6.5 μm, which efficiently enhances ion selectivity through their sophisticated pore morphology^27^. This phenomenon aligns with a well-recognized principle in membrane science: fewer and more refined channels typically result in lower membrane permeability but higher selectivity^29^. Fig. 1c shows the Fourier Transform Infrared Spectroscopy (FTIR) spectrum of unmodified and modified wood samples. For S-CWM, the peak at 1717 cm^−1^ corresponds to the carbonyl stretching vibration of polyacrylic acid (PAA), and the broad band at 3000–3600 cm^−1^ originates from the O–H stretching vibrations of PAA. For S-AWM, the 1717 cm^−1^ peak is attributed to carbonyl stretching vibrations of poly(2-(dimethylamino)ethyl methacrylate) (PDMAEMA), and the absorption bands around 2940 cm^−1^ arises from C–H stretching of the methyl and methylene groups of PDMAEMA^30^. By comparing the spectra of S-AWM and S-CWM at different locations (surface, Cutaway 1, Cutaway 2), we observe that these characteristic peaks are present in all tested positions, indicating the homogeneous distribution of polyelectrolytes (PAA in S-CWM and PDMAEMA in S-AWM) throughout the wood structure. In contrast, the pristine wood spectrum lacks these peaks, which confirms the successful polymerization and grafting of the polyelectrolytes.

We chose to use wood for two main reasons. Firstly, pure polyelectrolyte networks tend to become hydrogels in aqueous media, which can lead to compromised mechanical properties. By incorporating natural wood, its intrinsic mechanical rigidity enhances the mechanical strength of the resulting polyelectrolyte hydrogel composite, effectively addressing the mechanical weakness of pure polyelectrolyte hydrogels^31^. Secondly, the unique hierarchically porous structure of natural wood provides a high specific surface area, enabling a large surface area of surface-encapsulated polyelectrolyte^24^. This facilitates rapid ion and electron transportation while maintaining a high bio-based proportion. This characteristic is crucial for wood-based surface-encapsulated polyelectrolyte power generators (S-AWM and S-CWM). The cross-sectional SEM images of S-AWM and pristine wood indicate that S-AWM features surface modification of wood tracheids in nano- or microscale, while the feature structure of wood is not disturbed (Fig. 1d, e). Theoretically, this sunlight-induced surface-encapsulation strategy can be universally extended to other wood species and hydroxyl-rich cellulosic scaffolds^32^.

## Results and discussion

### Stability in electrical performance

**Fig. 2:**
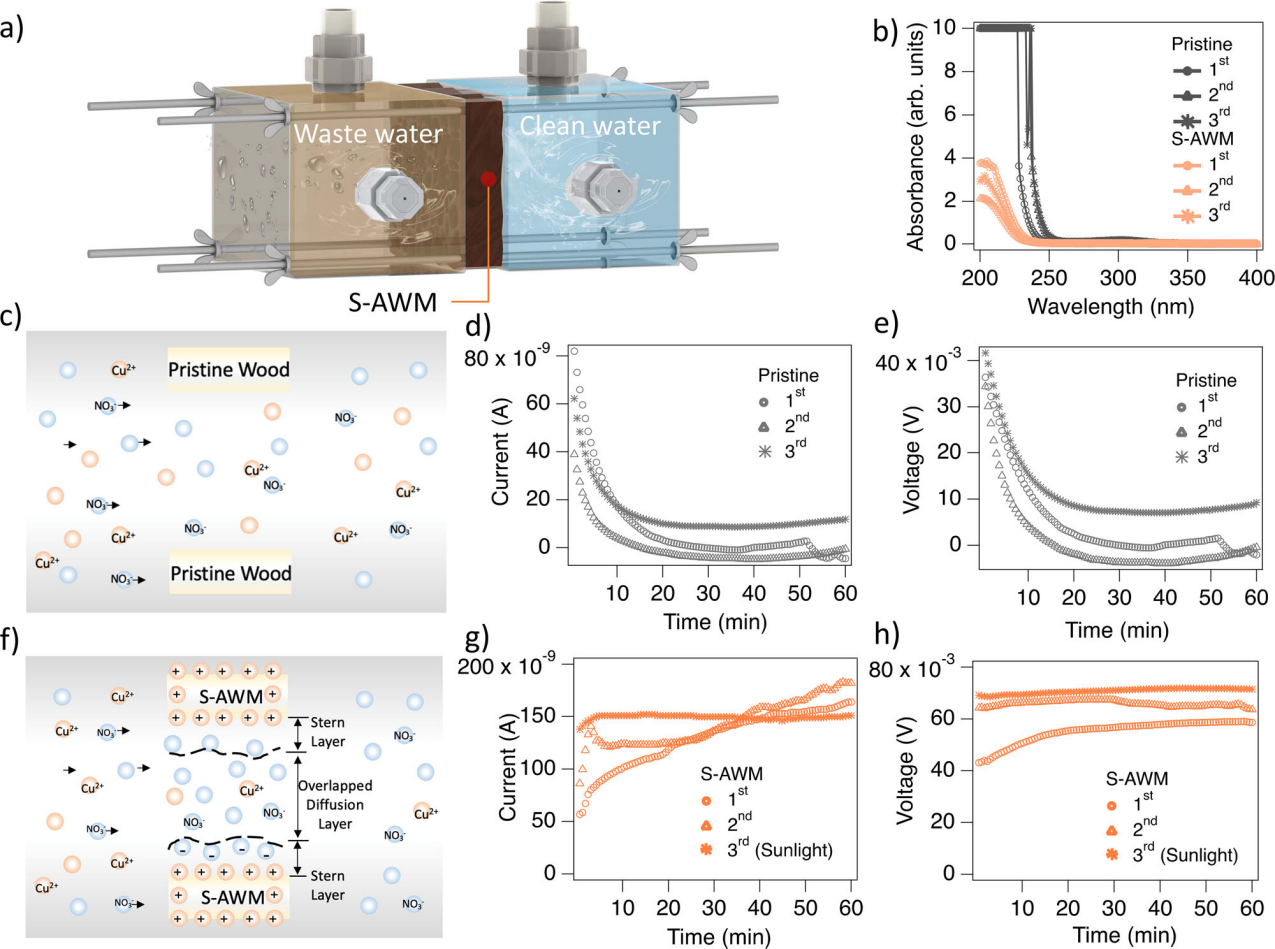
Ion transport mechanism and osmotic energy conversion performance of S-AWM in Cu(NO_3_)_2_ concentration gradients. **a** Scheme of half-cell setup. This setup was used for fundamental studies on electric performance, including the data in Figs. 2, 3, and 4. **b** Ultraviolet-visible spectroscopy (UV-vis) spectra of the clean water chamber after 1 hour of energy generation using pristine wood samples and S-AWM samples. Schematic illustration of the ion transport mechanism in (**c**) pristine wood and (**f**) S-AWM. **d**, **e**, **g**, **h** are energy conversion performance recorded in 10 mM Cu(NO_3_)_2_/water concentration gradient: (**d**) Short-circuit current (I_SC_) of pristine wood. **e** Open-circuit voltage (V_OC_) of pristine wood. **g** I_SC_ of AWM. **h** V_OC_ of AWM. In Fig. d, e (pristine wood), the samples labeled “1^st^”, “2^nd^”, and “3^rd^” are repeat samples prepared under identical conditions. In Fig. g, h (S-AWM), the “1^st^” and “2^nd^” samples are repeats synthesized via UV chamber-induced polymerization, while the “3^rd^” sample were fabricated through real-world sunlight-initiated photopolymerization conducted in Zurich, Switzerland, from 8:00 to 20:00 on 4 August 2022.

The remarkable power generation and excellent ion selectivity of AWM and CWM have opened up new opportunities for energy harvesting from wastewater. In our experimental setup, the wood-based surface-encapsulated ion-selective power generator was positioned between two half-electrochemical cells containing different concentrations of Cu(NO_3_)_2_ solutions, allowing us to extract osmotic energy (Fig. 2a). The osmotic power generators operate through two mechanisms: (1) Diffusion-osmotic mechanism: Ions present in the solution tend to diffuse from the high-concentration side to the low-concentration side through the membrane, resulting in a directional flow of cations or anions. This salinity gradient energy produces electrical energy through Gibbs free energy in the mixing of two electrolyte solutions with different levels of activity^33^. (2) Reverse electrodialysis (RED): Positive and negative ions selectively transport across the membrane^34^. As depicted in Fig. 2b, c, pristine wood reveals less efficient ion selectivity compared to the modified samples, the former presents mainly a pressure-driven mechanism (with a small portion of ion selectivity realized by -COOH from hemicellulose), as the salt gradient reaches equilibrium, the osmotic pressure induced by the gradient difference significantly decreases. While AWM and CWM support the RED mechanism (Fig. f), they show stable and continuous power output. This observation aligns with the open-circuit voltage (V_OC_) and short-circuit current (I_SC_) generated in the presence of a 10 mM Cu(NO_3_)_2_/water concentration gradient (Fig. 2d, e and g, h).

Notably, an observable difference in behavior exists between polymerization methods (Fig. 2g, h). The ‘1^st^’ and ‘2^nd^’ samples, synthesized via rapid high-intensity UV chamber curing, exhibit a gradual increasing trend in current and voltage. This is attributed to the fast polymerization kinetics creating a denser, internally stressed polymer network that requires a prolonged hydration and structural relaxation period to fully open the ion transport channels. Conversely, the ‘3^rd^’ sample, polymerized slowly under mild, natural sunlight over 12 h, demonstrates an immediately stable power output. The slow curing kinetics facilitate the formation of a more uniform, relaxed, and structurally equilibrated polyelectrolyte layer, eliminating the initial hydration lag and highlighting the distinct microstructural superiority of the natural sunlight approach.

### Feature parameters for ion selectivity and energy generation

**Fig. 3:**
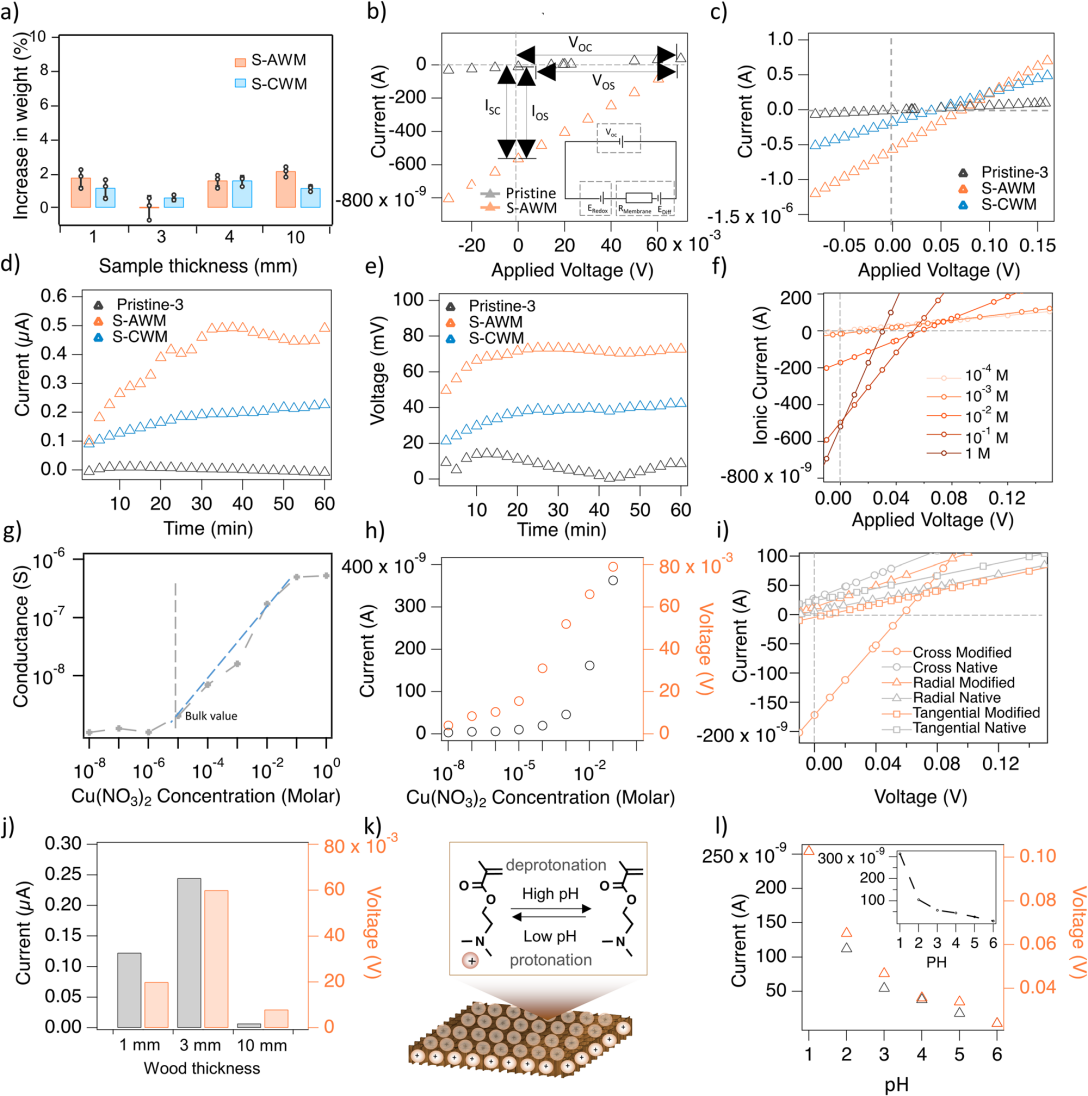
Structural and electrochemical characterization of polyelectrolyte-modified wood membranes and optimization of osmotic energy generation. **a** Increase in weight percentage for S-AWM and S-CWM, calculated from weight values before and after polymerization. Data are presented as mean values ± SD from three independent experiments (*n* = 3). Individual data points are shown as dots. **b** Representative I-V (current-voltage) curves of wood osmotic energy devices obtained when 100 mM Cu(NO_3_)_2_ solution was placed in the left reservoir and 1 mM Cu(NO_3_)_2_ solution was added in the right reservoir. The inserted illustration intercepts the current and voltage axes, corresponding to I_SC_ and V_OC_, respectively. **c** Current-voltage characteristics for pristine and polyelectrolyte surface-encapsulated wood osmotic energy generators obtained in 100 mM/1 mM concentration gradient. **d** I_SC_ of 3 mm-thick pristine wood, S-AWM and S-CWM osmotic generators, generated in 100 mM/1 mM concentration gradient. **e** V_OC_ of 3 mm-thick pristine wood, S-AWM and S-CWM. **f** I-V curves of S-AWM osmotic generators at concentrations from 10^−4 ^M to 1 M Cu(NO_3_)_2_ solution. Lines connecting data points are straight-line connectors and do not represent linear fits. **g** Ionic conductance of the membrane versus electrolyte concentration. The transmembrane ion conductance (black plus sign) apparently deviates from the bulk value (blue dash line) when the electrolyte concentration is < 10^−5^ M, indicating that ion transport is governed by surface charge rather than bulk conductance. The vertical dashed line marks the critical concentration of 10^−5 ^M below which surface-charge-dominated transport prevails. **h** Measured I_SC_ and V_OC_ under different concentration gradients of Cu(NO_3_)_2_ solution. **i** I–V curves of S-AWM osmotic generators featuring different wood planes, obtained in 10 mM/1 mM concentration. Lines connecting data points are straight-line connectors and do not represent linear fits. **j** Measured I_SC_ and V_OC_ using different wood thickness. **k** Protonation and deprotonation of S-AWM. **l** Measured I_SC_ and V_OC_ under different pH conditions.

S-AWM and S-CWM are designed with prioritize environmental friendliness and material efficiency, with over 98 wt% of the materials being biobased (Fig. 3a), utilizing the excellent mechanical properties as well as the high sustainability of wood. This is due to the polymer encapsulated on the lumen surface of wood tracheids in the form of PDMAEMA ultra-thin films, which effectively screens negative charges^35^. The efficient ion-selective functionality can be still provided so that the transportation of counterions can be selectively mediated through the surface-encapsulated PDMAEMA chains.

To investigate the ion transport behavior and voltage output performance of pristine wood and our modified S-AWM/S-CWM membranes under salinity gradient conditions, we measured their I-V responses (Fig. 3b, c) using an electrical cell (Fig. 2a). In Fig. 3c, S-AWM (positive) and S-CWM (negative) both show positive VOC shift vs pristine wood. This is because pristine wood lacks ion selectivity, while both modified membranes enable selective ion transport under a salinity gradient, generating a consistent electric field for positive VOC. The V_OC_ and I_SC_ can be obtained from the intercept on the current axis and the voltage axis, respectively. The redox potential (E_redox_) stems from the nonequal potential drop at the electrode-solution interface. Specifically, rather than originating from the oxidation or reduction of the wastewater itself, this E_redox_ arises because the two fluid chambers possess unequal salt concentrations. Consequently, the Ag/AgCl testing electrode in each chamber experiences a distinct local Nernstian potential, which creates a baseline “non-equal potential drop” across the measurement circuit. The pure osmotic potential (V_OS_) contributed by a power source can be obtained by subtracting the contribution from the electrode-solution interface at different concentrations^36^:

1 $${V}_{{os}}={V}_{{OC}}-{E}_{{redox}}$$

While power generation by pristine wood decreases due to equilibrium in salinity concentration, continuous power generation can be achieved by S-AWM without the need for external voltage input. We conducted stability tests on I_SC_ and V_OC_ generated by S-AWM over a 60 min duration (Fig. 3d, e). The corresponding short-circuit current and open-circuit voltage curves exhibit long-term stability with fluctuation, indicating continuity of electricity generation. In the absence of external voltage, Cu^2+^ and NO_3_^-^ ions diffuse from high concentration to low concentration, and a net current is detected only when the diffusion amount of one ion exceeds that of the other. Pristine wood membranes primarily demonstrate energy conversion dominated by salt gradient diffusion, while S-AWM and S-CWM exhibit an ion-selective energy generation process. The current generated by the S-AWM setup is higher than that generated by S-CWM. This difference in energy conversion performance can be attributed to their respective bulk mobilities (56.6 cm^2^/V·s for Cu^2+^ and 71.5 cm²/V·s for NO_3_^-^)^37^.

To elucidate the influence of concentration gradients on the energy conversion performance of the membranes, we systematically investigated this behavior by gradually increasing the Cu(NO_3_)_2_ concentration from 10^−4 ^M to 1 M, while keeping water on the other side (Fig. 3f–h). As the concentration gradient increases, the produced current densities increase, and the V_OC_ only begins to decrease at 1 M solution. This is proposed to be attributed to the small feature size of the polyelectrolyte network, which establishes a strong ion concentration polarization zone, even under high ionic strength conditions^38^. The ionic conductance of the S-AWM and S-CWM membranes exhibits a dependence on the electrolyte concentration (Cu(NO_3_)_2_). In concentrated salt solutions, where the Debye length (λ_D_) is far below the pore diameter, ion transport follows bulk behavior. However, the transmembrane conductance deviates significantly from the bulk value (gray dashed line) when the electrolyte concentration drops below 10^−5^ M, and λ_D_ approaches the channel dimension. This suggests that ion transport is controlled by surface charge^39^. Six configurations were employed to study the wood planes, as illustrated in Fig. 3i. In the case of modified cross-sections, the absolute values of V_OC_ and I_SC_ were 63 mV and 0.18 µA, respectively. However, in the case of cross-sectional pristine wood, both V_OC_ and I_SC_ decreased to around 0.

To elucidate how structural parameters influence the energy conversion performance of ion-selective wood membranes, we investigated the effect of S-AWM thickness on its performance under varying salinity concentrations (Fig. 3j and Supplementary Fig. S2). With a 3 mm thickness of S-AWM, we achieved a maximum V_OC_ of 60 mV and I_SC_ of 0.24 µA obtained in 10 mM/0.01 mM concentration gradient of Cu(NO_3_)_2_ solution. The electrical performance of both S-AWM and S-CWM is highly sensitive to pH levels due to the polyelectrolyte sidechains within the polymer networks. Specifically, PDMAEMA exhibits a higher degree of deprotonation at elevated pH values (approaching 6) and becomes protonated at lower pH values (approaching 1) (as shown in Fig. 3k, l). Figure 3l illustrates that as the solution on the left side of the membrane becomes more acidic (lower pH), both the generated current and voltage increase. Notably, at pH 1, there is a short circuit current of approximately 250 nano-amperes and an open circuit voltage of 0.1 V. This outcome aligns with our hypothesis, as a lower pH results in a higher concentration of H+ ions in the solution, leading to a more protonated state in S-AWM. Consequently, the membrane carries a stronger positive charge, resulting in greater repulsion of the positively charged copper ions.

**Fig. 4:**
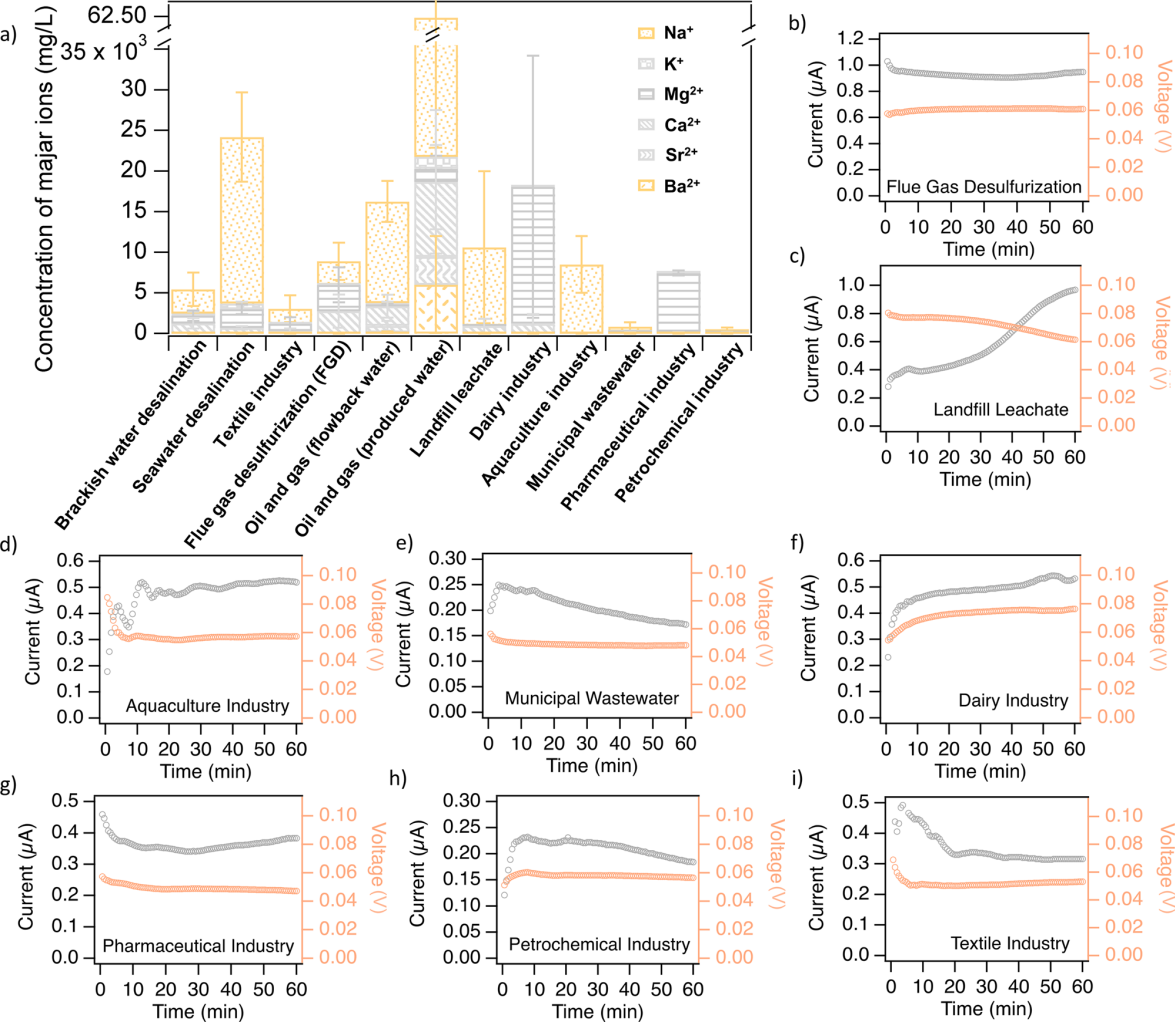
Osmotic energy harvesting from simulated industrial wastewater brines using S-AWM. **a** The main characteristics of brine effluents from different industries, compiled from Panagopoulos et al. ^2^. Bars represent the median ion concentrations and error bars represent the full range of values reported in the literature. **b**–**i** are I_SC_ and V_OC_ generated by S-AWM corresponding to different reaction time from t = 0 to t = 1 h, measured in different industry wastes. The container on the opposite side always contains clean water. The concentration of each characteristic brine solution was simulated as the median value in (**a**), see Supplementary Table S1.

Despite this impressive initial performance at extreme acidity, the long-term chemical durability of the device must be carefully contextualized within varying industrial environments. To rigorously evaluate the operational robustness, extended continuous stability tests were conducted at pH 1 (Supplementary Fig. S3). The results demonstrate that while the S-AWM maintained a stable open-circuit voltage and a steady short-circuit current plateau for the first ~ 1000 min, prolonged exposure eventually led to a decline in current. This degradation is primarily attributed to the acid-catalyzed hydrolysis of hemicellulose and amorphous cellulose, compromising the wood’s structural integrity. Because natural wood is intrinsically sensitive to such prolonged exposure to extreme acidic or alkaline conditions, the current S-AWM is ideally suited for mildly acidic to neutral wastewater streams (e.g., pH 4–8). This operational window effectively covers a vast subset of effluents, including municipal, dairy, and aquaculture wastewater (as simulated in Fig. 4). Within these applicable environments, the inherent hierarchical structure of wood, reinforced by its highly cross-linked lignin matrix, provides significant dimensional stability against macroscopic swelling. Furthermore, the covalent crosslinking of the polyelectrolyte layer via sunlight-induced polymerization prevents rapid polymer leaching under varying ionic strengths. Nevertheless, we acknowledge that for continuous operation over weeks or months in real-world organic-rich wastewater or chemically fluctuating wastewater, wood-based substrates may be susceptible to bio-fouling and slow microbial degradation, and chemical corrosion. Therefore, for industrial relevance and real-world implementation, the execution of standard pre-treatment protocols, such as pH buffering to neutralize highly aggressive streams, alongside coarse filtration to remove organic fouling species, will be essential to preserve the structural integrity of the wood scaffold and prolong the device’s operational lifespan.

To better understand how the polyelectrolyte incorporation influences the electrical performance of the wood electricity generator, 1 mm-thick wood was designed to cooperate with infiltrated and in situ polymerized polyelectrolyte network, leading to infiltrated lumina-filling wood ion-selective power generators (I-AWM and I-CWM, 1 mm) as a comparison to S-AWM and S-CWM. 1 mm-thick pristine wood possesses abundant open tracheids, enabling high water flux but yielding low osmotic current. This is primarily because pristine wood lacks effective ion-selective mechanisms: its tracheid pore size ( ≈ 10–100 μm) is far larger than ion diameters (nm scale), leading to negligible steric effect; meanwhile, the weak charge density of native wood polymers (cellulose/hemicellulose) results in a negligible Donnan effect. Without these intrinsic separation mechanisms, pristine wood cannot achieve selective ion transport, even before considering the ion concentration polarization (ICP) phenomena that further impair selectivity under high salinity conditions^40,41^. When the amount of both-sided open tracheids is large, it possesses the unique property of fast water transport through open tracheids, while closed channels impede water flow^40^. This makes them ideal for energy harvesting from highly saline solutions with high osmotic pressure. In particular, 1 mm wood samples demonstrate fast water flow but lack ion selectivity, necessitating the incorporation of more dense ion-selective polymer networks. Therefore, we introduce the concept of infiltrated lumina-filling samples, namely I-AWM and I-CWM, composed of polyelectrolyte infiltrated networks synthesized through atmospheric infiltration and in situ UV-induced polymerization (Supplementary Fig. S4). The synthesis process significantly increases the weight of I-AWM and I-CWM, with wood thicknesses of 1 mm gaining over 40% weight after polymer modification (Supplementary Fig. S5). However, the weight increase diminishes with increasing thickness due to inefficient infiltration in thick wood, likely caused by incomplete atmospheric infiltration employed during the process. Similar to S-AWM, S-CWM, and other porous osmotic generators, thin wood-based generators attract counter ions while repelling ions of like charge. Notably, the incorporation of chemically crosslinked polyelectrolyte networks in I-AWM and I-CWM amplifies this phenomenon by narrowing the channels formed during the layer-by-layer (LBL) process. E_OS_ arises from the difference between the diffusive fluxes of positive and negative ions due to the ion selectivity of I-AWM and I-CWM. Hence, short circuit current and open circuit voltage generated from I-AWM and I-CWM are a bit higher than S-AWM and S-CWM (Figs. S6–S10, and Fig. 3c–e). The ion transference number ${t}_{n}$ is shown in Supplementary Fig. S11, the calculated mobility μ_+_/μ_−_ of I-AWM is 1.73. The diffusion current reached its maximum on I-AWM at C_high_/C_low_ = 10 mM/1 mM, and the current density reached its peak value 22 mA m^−2^ (Supplementary Fig. S12). Unlike S-AWM, where both microtome-cut and saw-cut samples feature polyelectrolyte thin-film encapsulation on wood tracheids and exhibit a morphology similar to pristine wood (Supplementary Figs. S13–S15 and Fig. 1e), I-AWM showed completely filled lumen in both microtone cut and saw cut samples (Figs. S16–S18). The transport behavior can be regulated by modifying the surface charge properties or adjusting the nanochannel size. One of the most straightforward ways to control the nanochannel size is by adjusting the crosslinking degree of the polymer network.

To investigate the structure-transport relationship and understand how nanochannel size influences ion selectivity, we evaluated the energy conversion behavior of S-AWM under a 100 mM/1 mM salinity gradient across three polymer crosslinking degrees (1%, 5%, and 50%) (Supplementary Fig. S19). The results indicate that an intermediate crosslinking degree (5%) yields the optimal electrical output, whereas a highly crosslinked network (50%) exhibits a dramatic decline in both *I*_*sc*_ and *V*_*oc*_. Mechanistically, a low-to-intermediate crosslinking degree allows the polyelectrolyte chains to remain extended, providing effective overlapping Debye screening lengths for ion selectivity while retaining high free volume for water permeability. Conversely, while highly crosslinked networks possess a higher localized charge density per unit volume, they rigidly restrict the effective free volume size of the channels. This creates excessive steric hindrance for ion mobility and significantly increases the internal membrane resistance, resulting in lower permeability to water^42^. Therefore, achieving optimal ion transport efficiency (*t*_*n*_ ~ 0.6-0.7, Fig. S11) dictates a careful balance: providing sufficient charge density for Donnan exclusion while maintaining adequate free volume for high-flux ionic mass transport. As actual energy generation is also significantly affected by operating conditions, such as the load resistor and solution flow rates, which determine the boundary layer thickness^43^, optimizing this internal structural balance is critical. Considering the high mechanical properties and high biobased proportion^44^, S-AWM and S-CWM are more relevant in the context of applicability and sustainable development in real-world applications.

### Scalability and viability in water-energy circular economy cycles

The two fluid reservoirs separated by S-AWM were filled with modulated brine effluents from different industries. The concentration of each characteristic brine solution was simulated as a median value in Fig. 4a plotted from data in literature^2^, with a fixed volume of 150 mL. The current-voltage curves for S-AWM were collected at different times and various salt concentrations (Fig. 4b–i). The values of I_SC_ and V_OC_ generated by S-AWM exhibit variations, and these differences can result from variations in the composition of the brine effluents. Specifically, when we have the same types of ions present, the concentration of these ions plays a crucial role. For instance, both textile wastewater and flue gas desulfurization wastewater contain ions like Ca^2+^, Na^+^, Mg^2+^, Cl^-^, and SO_4_^2-^. However, what’s noteworthy is that the electrical performance of the ion-selective power generator is notably better in the case of textile wastewater compared to flue gas desulfurization wastewater. This contrast can be attributed to the significantly higher ion concentration in flue gas desulfurization wastewater, which is around 4.5 grams per liter (g/L), as opposed to approximately 1.5 g/L in textile industry wastewater.

**Fig. 5:**
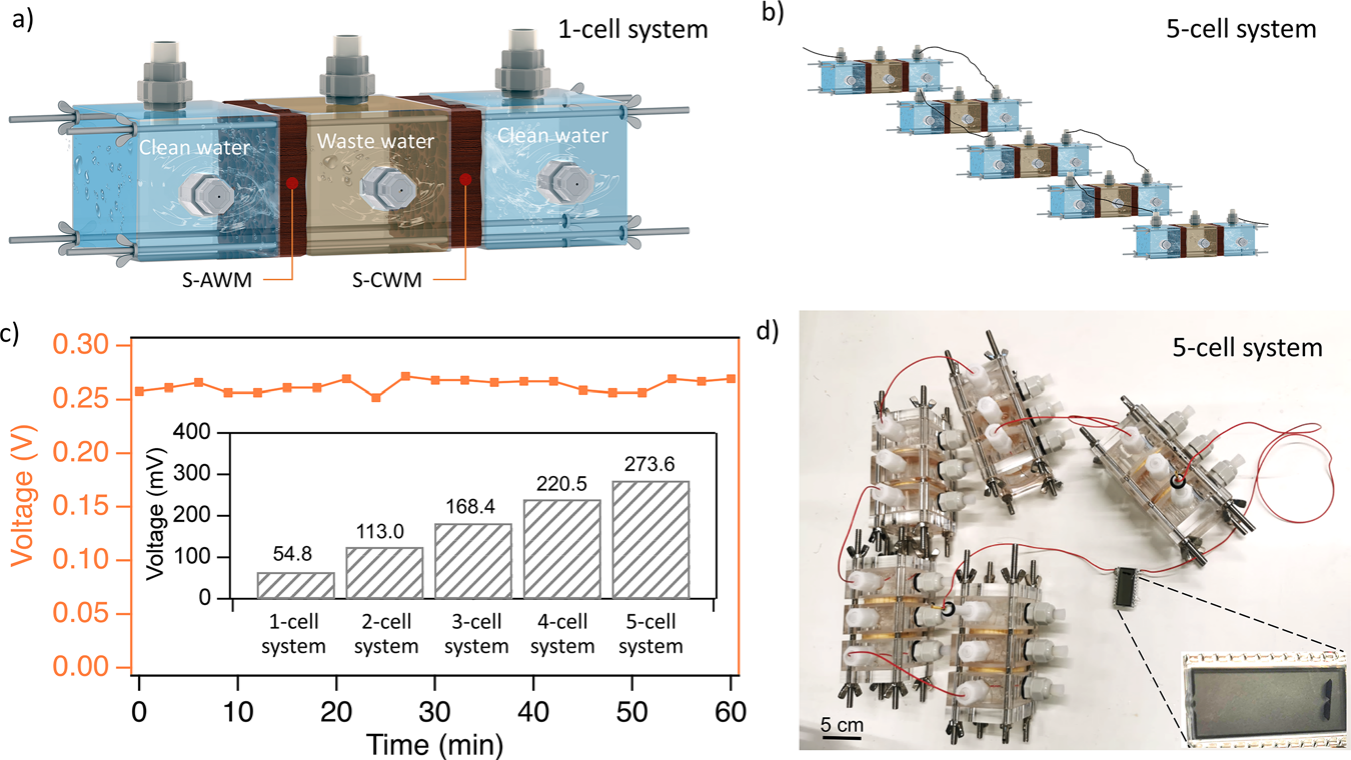
Scalable multi-cell ion-selective power generator for continuous electricity generation. **a** 1 cell system consists of two clean water channels and one wastewater channel. **b** Scheme of a 5-cell system. **c** Continuous electricity generated from a 5-cell system. Insert: the measured voltage value presents additivity upon multiple cells. **d** A five-cell small demonstrator of an ion-selective power generator powering a liquid crystal display (LCD) device.

The synthesis method introduced in this research embodies an environmentally sustainable and economically efficient approach, elevating it as a highly auspicious pathway for the practical implementation of sustainable osmotic energy generation. We not only furnish a viable design blueprint for the creation of osmotic electricity plants but also underscore their potential to substantially augment the symbiotic relationship between urban water systems and energy cycles. While the individual voltage output of one cell was about 0.055 V, the output voltage of five cells connected in series could reach 0.27 V, where each cell consists of two clean water chambers and one wastewater chamber (Fig. 5a–c). This suggests that we can improve electrical performance by adding more cells and using different electrical connection modes tailored to specific applications. To further demonstrate the feasibility for practical applications, we connected the demonstrator of wood based osmotic energy generator (5 cells connected in series) to a commercial light-emitting diode (LED) with conductive wires. As illustrated in Fig. 5d, the LED could be readily switched on when the wastewater was treated.

To realistically evaluate the pathway toward large-scale application, a quantitative assessment of the current power output is necessary. For a single S-AWM cell (active area of 1 cm^2^), the calculated maximum power density (*P*_*max *_= *I*_*sc*_ × *V*_*oc*_ / 4) is approximately 0.08 mW/m^2^. Transitioning from centimeter-scale blocks to module-scale assemblies will inevitably introduce engineering challenges, primarily dominated by increased internal resistance and ICP. To mitigate these limitations in future scaled-up plants, hydraulic management must be optimized. Thus, while the current scale-up demonstration is foundational, it paves the way for specific hydrodynamic engineering to realize industrial osmotic power modules. To contextualize the significance of this work, we benchmarked S-AWM against state-of-the-art osmotic membranes across structural integrity, fabrication scalability, and performance (Supplementary Table S2). While ultrathin 2D films and heavily processed 3D biomaterials can exhibit higher absolute power densities, they heavily rely on destructive chemical pre-treatments, unscalable vacuum filtration, and energy-intensive fabrication. Conversely, S-AWM strategically preserves the pristine wood matrix through a zero-energy, sunlight-driven process. Although sacrificing some peak areal power density, S-AWM possesses overwhelming advantages in environmental sustainability, energy efficiency, and industrial scalability. This makes the device particularly ideal for real-world industrial scenarios with abundant spatial footprints, such as large-scale holding ponds for flue gas desulfurization (FGD) or municipal wastewater, where spatial footprint is less of a limiting factor than the prohibitive capital cost of commercial membranes.

From a techno-economic perspective, while the current lab-scale power density yields a protracted payback period if evaluated solely on electricity sales, the capital expenditure (CAPEX) paradigm is highly disruptive. As previously reported^12^, achieving economically viable osmotic power requires membrane costs to fall below ~ $4.5/ m^2^. High-end commercial synthetic membranes (often costing $500–1000/ m^2^) are inherently excluded by this threshold. In contrast, the estimated material cost of S-AWM, comprising raw spruce (around $1–3/ m^2^), bulk monomers, and free sunlight polymerization, can practically approach this target.

Ultimately, by combining osmotic energy production with wastewater treatment, we contribute to a circular economy that emphasizes wastewater recycling and the promotion of cost-competitive renewable energy sources. The concept of water-energy economic cycles proposed in this paper highlights the importance of integrating these two critical resources in a sustainable and environmentally conscious manner.

In conclusion, our study has successfully demonstrated the remarkable potential of the designed ion-selective power generator composed of 98 wt% bio-based and low-cost resources. Their significant contributions to green and sustainable energy generation are emphasized by using modulated urban and industrial wastewater. These eco-friendly devices exhibit excellent cation/anion selectivity in continuous power generation, enabled by the covalent assembly of negatively or positively charged polyelectrolyte networks within microchannels. By integrating more cells using various electrical connection modes, the output voltage reaches 0.27 V with 5 small cell units(each cell, R × T × L: 20 × 20 × 3 mm^3^) in modulated wastewater from flue gas desulfurization, indicating the potential for scaling up and its viability for future applications in industrial osmotic electricity plants. These wood-based ion-selective power generators offer a sustainable and efficient solution, with the potential to significantly reduce carbon footprints and mitigate reliance on finite fossil resources. They represent a significant step towards a greener future, where renewable energy sources play a vital role in meeting our energy needs while preserving the environment for generations to come.

## Methods

### Chemicals and materials

Cross sections of native wood species, Norway spruce (*Picea abies, all* the wood materials are readily available from the authors, or from standard commercial sources.) with the dimensions of 20 mm × 20 mm × 3 mm (radial × tangential × longitudinal, R × T × L), 20 mm × 20 mm × 1 mm (R × T × L), 20 mm × 20 mm × 10 mm (R × T × L), were cut by saw. The thickness of the wood can be precisely tuned during the cutting steps. The following chemicals were used in the experiment as received: 2(dimethylamino)ethyl methacrylate (DMAEMA, 98%, Sigma-Aldrich), acrylic acid (AA, 99%, Sigma-Aldrich), ethylene glycol dimethacrylate (EGDMA, 98%, Sigma-Aldrich), copper nitrate (Cu(NO_3_)_2_, 99.999%, Sigma-Aldrich), calcium chloride (CaCl_2_, ≥ 93.0%, Sigma-Aldrich), sodium chloride (NaCl, 99.5 + %, for analysis, VWR international), sodium bicarbonate (NaHCO_3_, > 99.7% crystals, Fisher Scientific), sodium sulfate anhydrous (Na_2_SO_4_, ≥ 99.0%, Sigma-Aldrich), sodium phosphate dibasic heptahydrate (Na_2_HPO_4_^.^7H_2_O, ≥ 99.99%, Sigma-Aldrich), magnesium sulphate (MgSO_4_, anhydrous, ≥ 99.5%, Sigma-Aldrich), magnesium chloride (MgCl_2_, 99.9%, Thermo Scientific), potassium sulfate (K_2_SO_4_, ≥ 99.0%, Sigma-Aldrich), Zinc nitrate hexahydrate (Zn(NO_3_)_2_, 98%, Sigma-Aldrich), Cadmium nitrate (Cd(NO_3_)_2_, 98%, Sigma-Aldrich), Palladium nitrate dihydrate (Pb(NO_3_)_2_.2H_2_O, 99%, Sigma-Aldrich).

To represent typical real-world conditions, the simulated (“modulated”) wastewater for various industries was formulated based on the median concentrations of major ions reported in the literature^2^. The specific mass of salts added to 150 mL of water to achieve these simulated concentrations is detailed in Supplementary Table S1.

### Fabrication of S-AWM and S-CWM

Wood samples with different thickness (1 mm, 3 mm, 10 mm) were dip coated with polyelectrolytes via a simple and green method. The process employed neat monomers (no solvent added), specifically, positively charged DMAEMA for S-AWM and negatively charged AA for S-CWM to achieve atmospheric infiltration into the wood. Prior to polymerization, samples were dippedand lifted at a controlled speed of ~ 10 cm/min, with the ambient wood moisture content maintained below 30% to ensure uniform monomer inflitration. These samples are then subjected to sunlight-induced radical polymerization inside an economical UV chamber (UVACUBE 400) with a light intensity of 1000 W/m^2^. The polymerization process lasted for 12 h, resulting in the uniformly surface-encapsulation of wood tracheids with polyelectrolytes. These materials containing anion-selective and cation-selective polymer networks, were evenly distributed within both the wood’s lumen and cell walls. For the S-AWM samples presented in Fig. 2g, h: the ones labeled “1st” and “2nd” are repeat samples prepared following the aforementioned protocol, while the sample labeled “3rd” was synthesized via real-world sunlight-initiated photopolymerization conducted in Zurich, Switzerland, from 8:00 to 20:00 on 4 August 2022.

### Fabrication of I-AWM and I-CWM

I-AWM and I-CWM were fabricated using the same monomer systems as S-AWM and S-CWM, respectively: positively charged DMAEMA for I-AWM and negatively charged AA for I-CWM (no solvent added). Wood samples were first pre-immersed in the neat monomer solution for 3 h to ensure sufficient monomer penetration into the wood channels. Unlike the surface-encapsulation procedure for S-AWM and S-CWM, samples were continuously immersed in the monomer solution throughout the entire polymerization process to achieve interior encapsulation. Polymerization was then initiated using a UV chamber (UVACUBE 400) at a light intensity of 1000 W/m^2^ for 12 h, with the ambient wood moisture content maintained below 30%. The crosslinking degree was controlled by varying the monomer-to-crosslinker (EGDMA) ratio, using three ratios of 1, 5, and 50% (w/w).

### Material characterization

#### ATR-FTIR

Different cutaways in S-AWM, S-CWM were analyzed with an attenuated total reflectance Fourier-transform infrared (ATR-FTIR) spectrometer (Bruker Tensor 27) equipped with an ATR module over the scan range of 400–4000 cm^−1^ to analyze various cutaway sections in both S-AWM and S-CWM. This approach is used to determine whether the modification was homogeneous across the entire sample.

#### SEM

Wood samples were cut into a clear cross-sectional morphologies measuring 5 x 5 x 1 mm with using a microtome. To prevent surface charging during imaging, we sputter-coated (CCU-010 compact coating unit; Safematic) with 10 nm of platinum. A scanning electron microscope (SEM, Thermo Scientific, Ultradry) equipped with a LEO 1530 operating instrument (Zeiss GmbH, Germany) was used to capture the micrographs.

#### Electrochemical testing

The ion transport behavior and osmotic energy conversion experiments were carried out with an electrochemical workstation (Gamry Interface 1010E Potentiostat/Galvanostat/ZRA s/n 28180). The wood-based membrane was mounted between two interchangeable H-type membrane electrolytic tanks. The testing area covered approximately 1 cm^2^. Ag/AgCl electrodes were used to apply a transmembrane electrical potential, which remained stable throughout the testing. To create the series connections, multiple devices were connected using wires. Standard Ag/AgCl electrodes were utilized to convert the transmembrane ionic flux into electronic current via reversible faradaic reactions (Ag + Cl¯ ⇌ AgCl + e¯). Target wastewater ions do not directly participate as redox reactants, preventing immediate metal deposition. For future scaled-up applications, employing isolated electrode chambers with circulating rinse solutions will effectively mitigate long-term electrode fouling and corrosion.

### Reporting summary

Further information on research design is available in the Nature Portfolio Reporting Summary linked to this article.

## Supplementary information

Supplementary Information

Reporting Summary

Transparent Peer Review file

## Source data

Source Data 1

Source data 2

## Data Availability

All data needed to evaluate the conclusions in the paper are present in the paper and/or the Supplementary Materials. Additional data related to this paper may be requested from the authors. Source data are provided in this paper.
