# Supplementary material for: Continuous electricity from charged total dissolved solids in wastewater using a wood-based ion-selective power generator
Source: Nat Commun. 2026 Jul 9;17:8636. doi: 10.1038/s41467-026-75514-7 (PMC13486703; doi:10.1038/s41467-026-75514-7)
Supplement: Supplementary file 1 [file 41467_2026_75514_MOESM1_ESM.pdf]

Supplementary Information

Continuous Electricity from Charged Total Dissolved Solids in Wastewater Using a Wood-based Ion-Selective Power Generator

Wenqing Yan[1 #], Jianguo Sun[1 #], Muze Han[2,3], Jonas Garemark[1], Maria F. Cortes Ruiz[4], Federico M. Orlando[5], Sophie M. Koch[1,6,7], Liyuan Bao[8], Evamaria Fuchs[1], Özen Eroğlu[1], Christopher Hubert Dreimol[1,4], Yong Ding[1,6], Feng Chen[1,9], Lavinia Heisenberg[10,11], Shihang Li[2,3]*, Kunkun Tu[1,2]*

[1]Wood Materials Science, Institute for Building Materials, ETH Zürich, 8093 Zürich, Switzerland

[2] Jiangsu Key Laboratory of Coal–based Greenhouse Gas Control and Utilization, Carbon Neutrality Institute, China University of Mining and Technology, Xuzhou 221008, China

[3] School of Environment Science and Spatial Informatics, China University of Mining and Technology, Xuzhou 221116, China

[4]Wallenberg Wood Science Center, Department of Fiber and Polymer Technology, KTH Royal Institute of Technology, SE-10044 Stockholm, Sweden

[5]The Durability of Engineering Materials Group, Institute for Building Materials, ETH Zürich, 8093 Zürich, Switzerland

[6]WoodTec Group, Cellulose & Wood Materials, Empa, 8600 Dübendorf, Switzerland

[7] Department of Natural Sciences and Sustainable Resources, Institute of Wood Technology and Renewable Materials, BOKU University, Konrad Lorenz-Straße 24, Tulln/Donau 3430, Austria

[8]School of Digital Technology and Innovation Design, Jiangnan University, No.1800 Lihu Boulevard, Binhu District, Wuxi City, Jiangsu Province, P.R.China

[9]Key Laboratory of Optoelectronic Chemical Materials and Devices, Ministry of 7 Education, Jianghan University, Wuhan, 430056, China

[10]Institute for Theoretical Physics, ETH Zürich, Wolfgang-Pauli-Strasse 27, 8093, Zürich, Switzerland

[11]Institute for Theoretical Physics, Heidelberg University, Philosophenweg 16, 69120 Heidelberg, Germany

[#]These authors contributed equally

* Corresponding author: shihangli@cumt.edu.cn; tuk@cumt.edu.cn

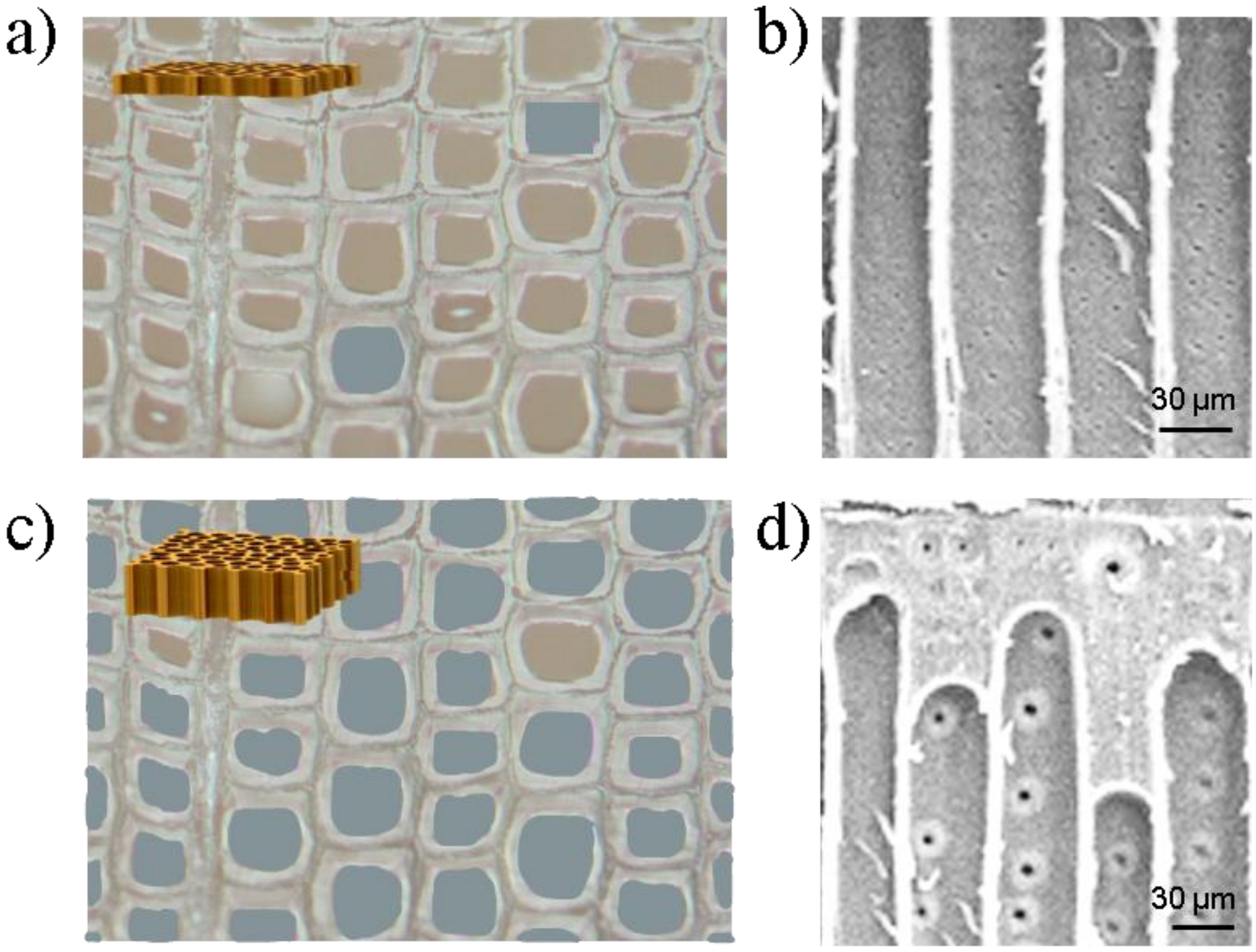

Figure S1. Structural characterization of native spruce wood cross-sections at different thicknesses. a) Scheme of 1 mm-thick native spruce on cross section. Grey filler in lumen represents closed tracheids. b) SEM image of radial plane of 1 mm-thick native spruce, where tracheids are open from both ends. c) Scheme of 3 mm-thick native spruce on cross section. Grey filler in lumen represents closed tracheids. d) SEM image of radial plane of 3 mm-thick native spruce, it contains tracheids closed at least from one ends.

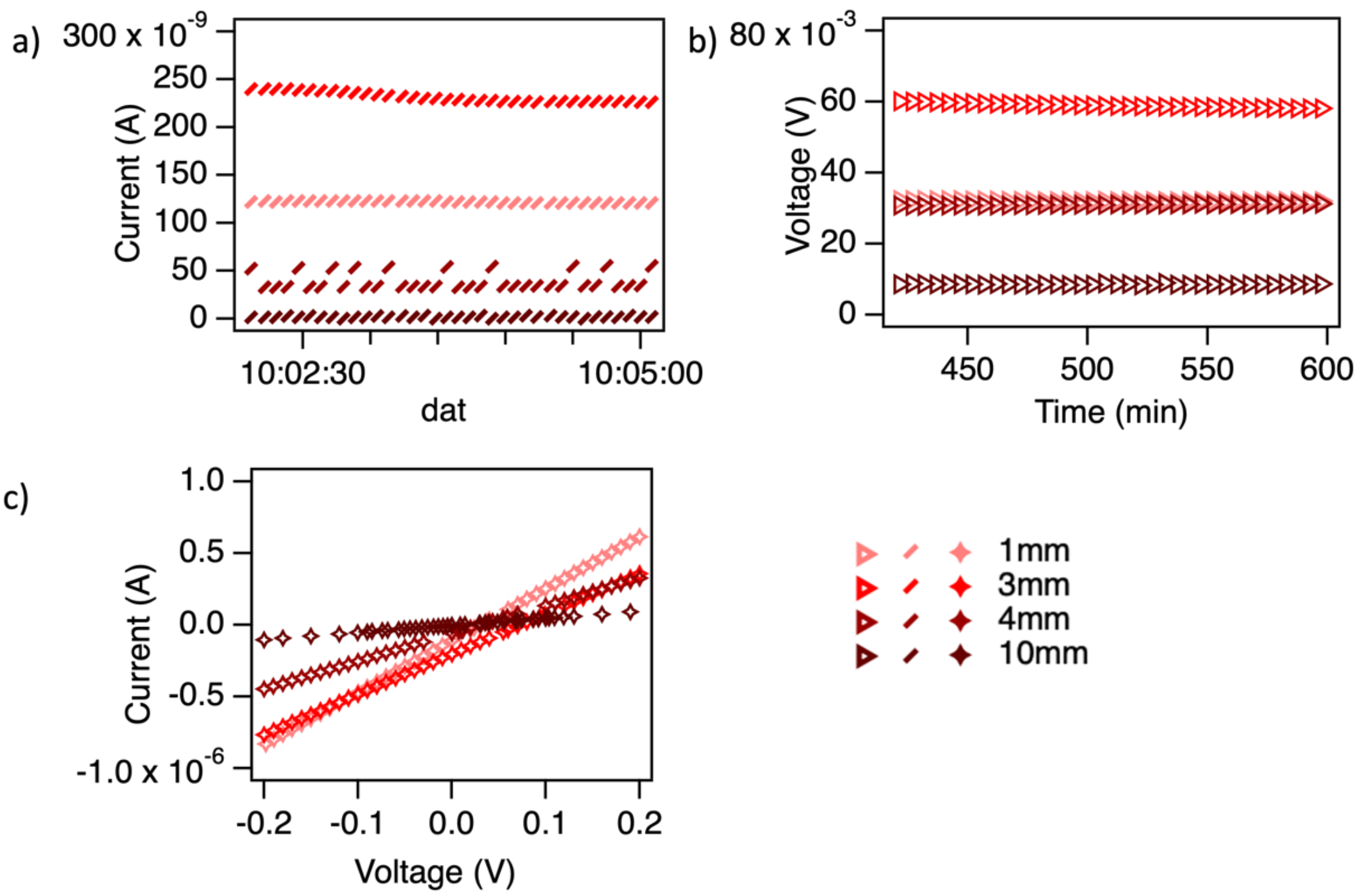

Figure S2. Effect of wood thickness on the osmotic energy conversion performance of S-AWM. a) I_{SC} of S-AWM with different thicknesses, generated in 100mM/1mM concentration gradient. b) V_{OC} of S-AWM with different thicknesses, generated in 100mM/1mM concentration gradient. c) I-V curves of S-AWM with different thicknesses, generated in 100mM/1mM concentration gradient.

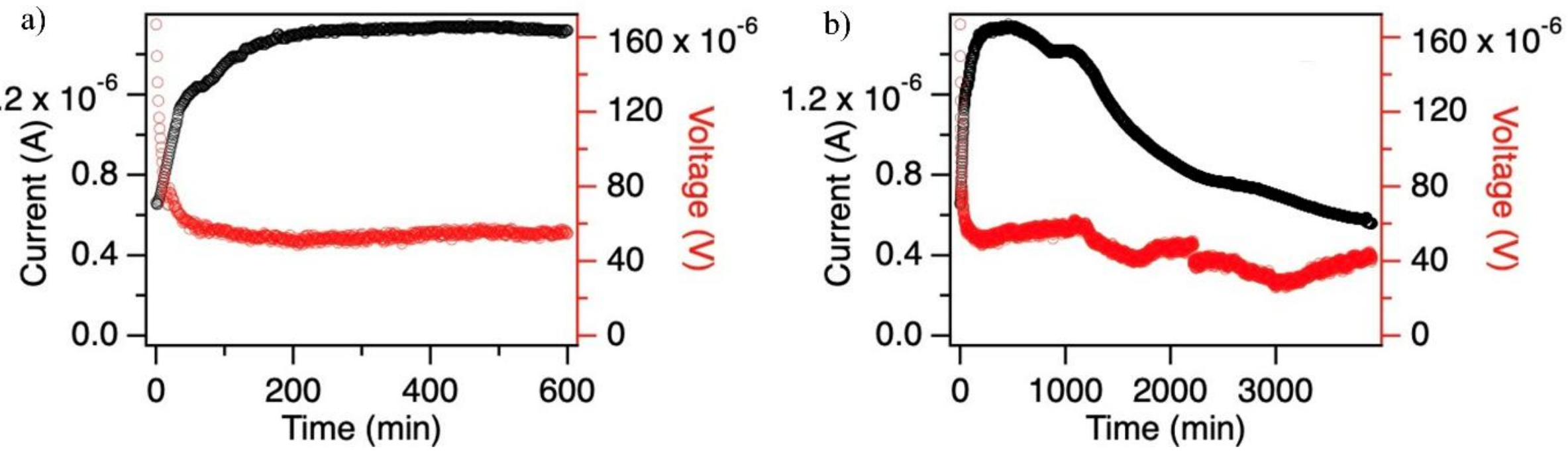

Figure S3. Acid resistance and long-term durability of the S-AWM osmotic power generator. The measurements were conducted under a concentration gradient of 1 mM $Cu(NO_3)_2$ solution versus deionized (DI) water. (a) I_{sc} and V_{oc} monitored over the initial 600 minutes. (b) Extended continuous stability test over 4000 minutes.

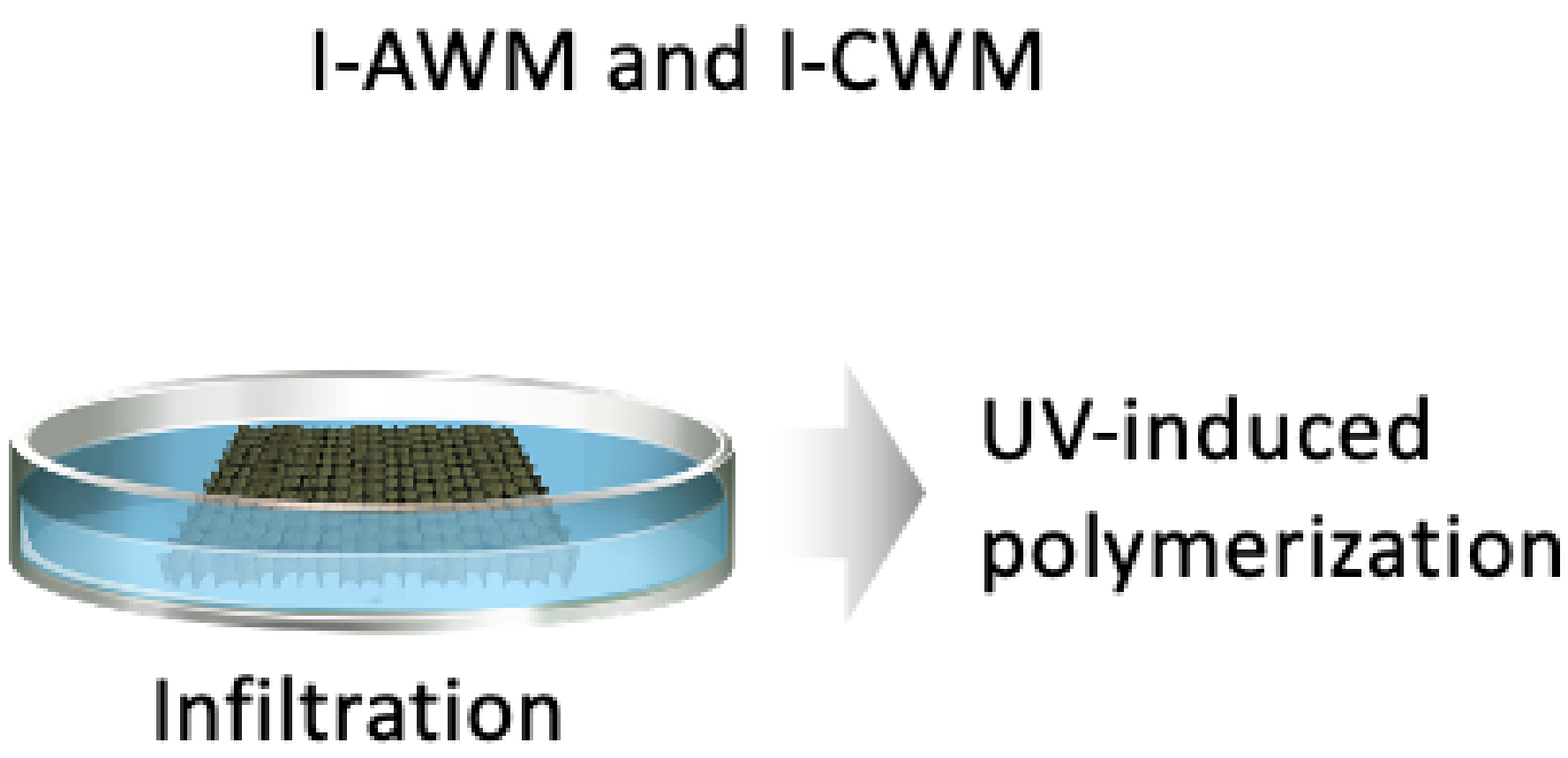

Figure S4. Schematic illustration of the fabrication process of interior-encapsulated wood membranes. Schematic fabricating process of I-AWM and I-CWM.

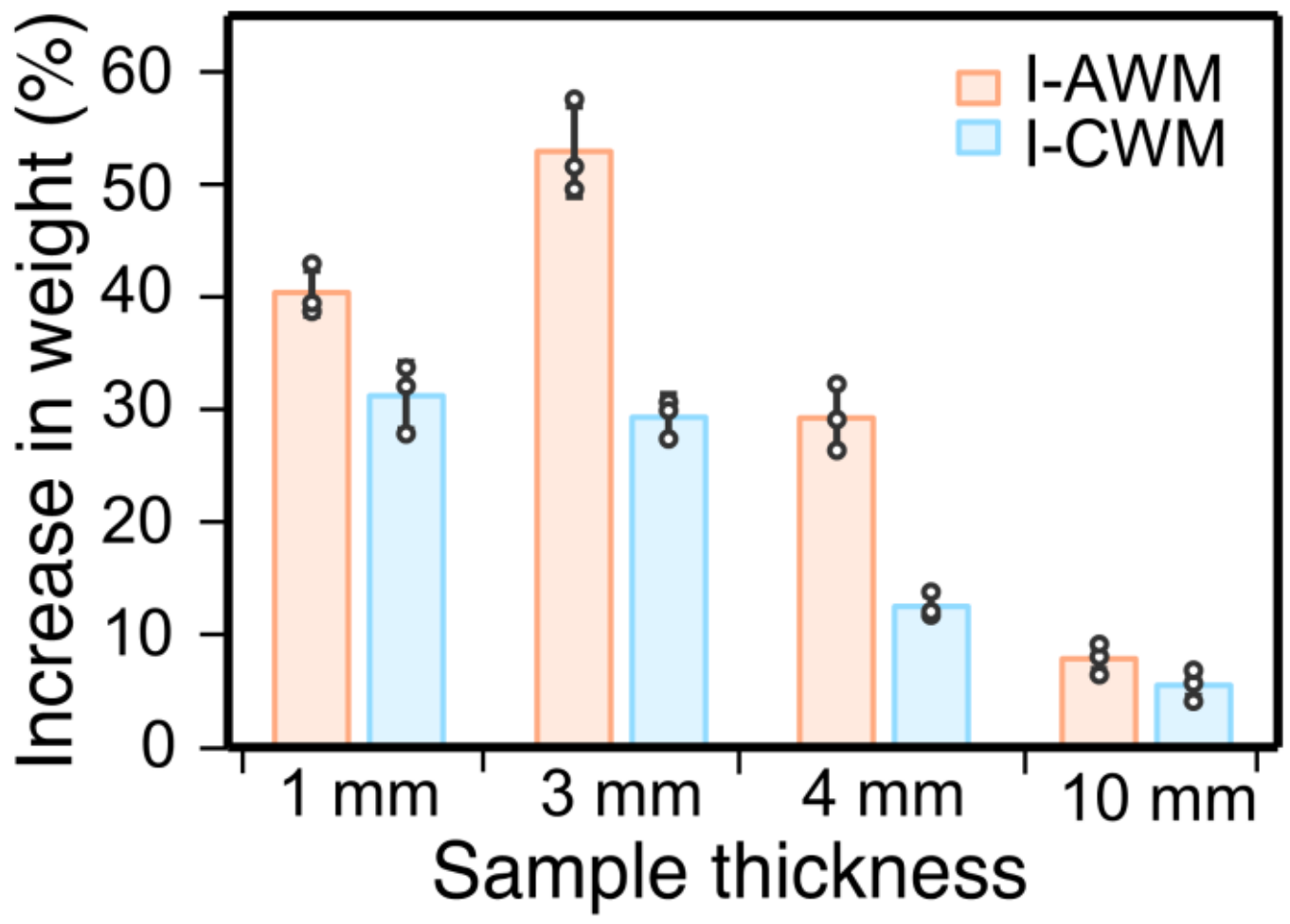

Figure S5. Gravimetric characterization of polyelectrolyte loading in I-AWM and I-CWM. Increase in weight percentage for I-AWM and I-CWM, calculated from weight values before and after polymerization. Error bars represent the standard deviations from three independent experiments (SD, n = 3).

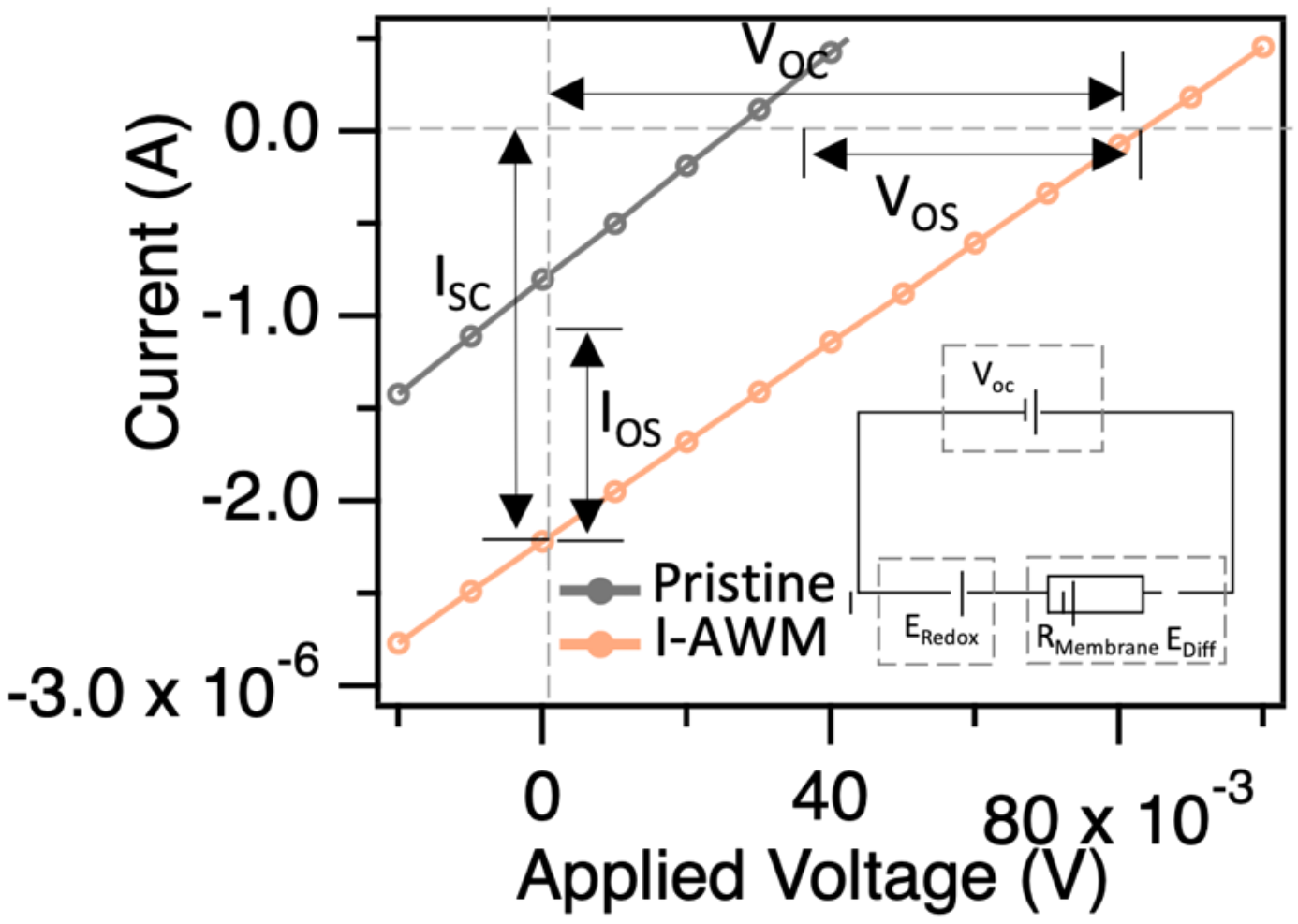

Figure S6. Osmotic energy output characteristics of wood-based devices across a 100-fold $Cu(NO_3)_2$ concentration gradient. Representative I-V curves of wood osmotic energy devices obtained when 100 mM $Cu(NO_3)_2$ solution was placed in the left reservoir and 1 mM $Cu(NO_3)_2$ solution was added in the right reservoir. The insert illustration is intercepts on the current and voltage axes, which are corresponding to I_{SC} and V_{OC} respectively.

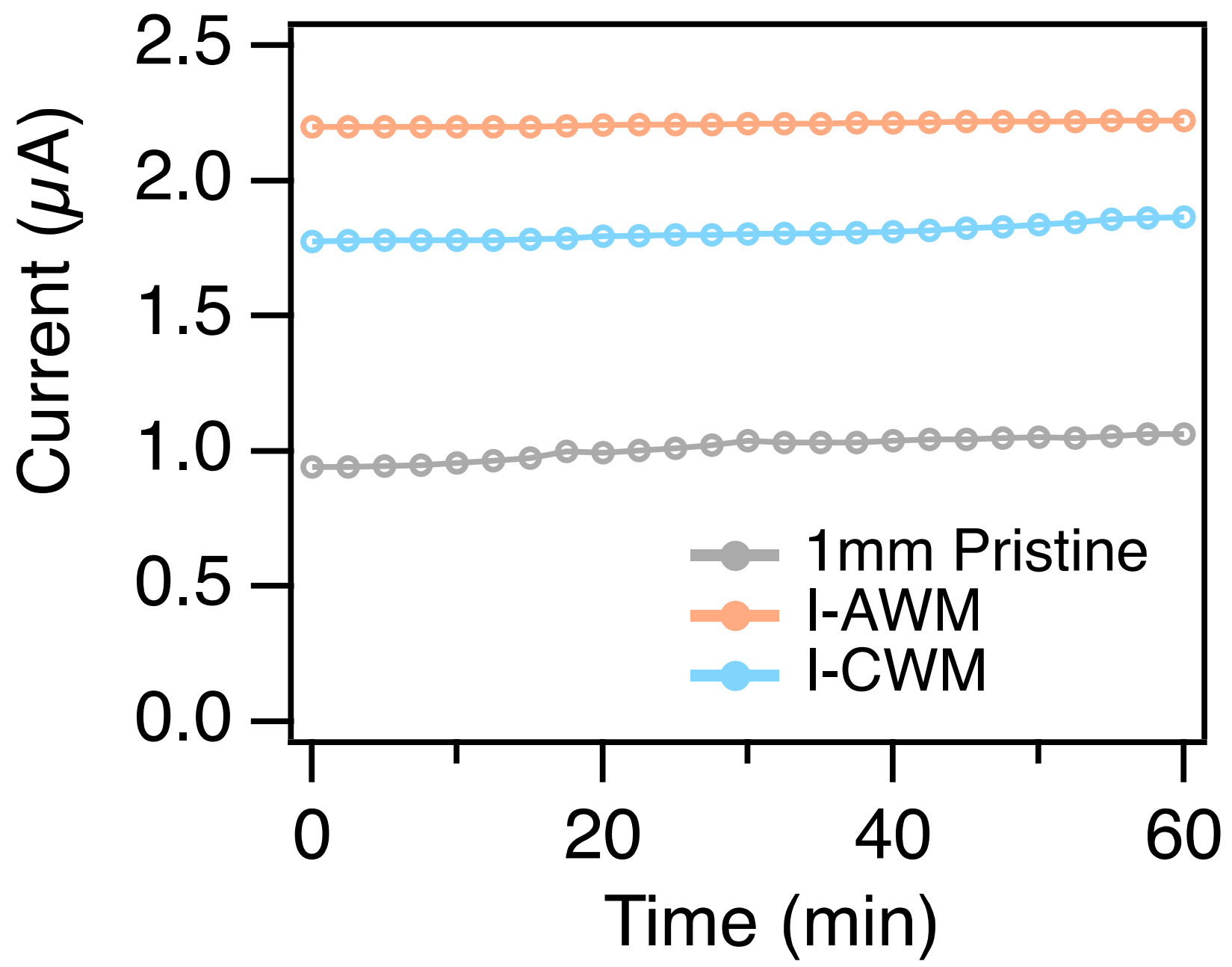

Figure S7. Short-circuit current comparison of 1 mm-thick pristine wood, I-AWM and I-CWM osmotic generators. I_{SC} of 1 mm-thick pristine wood, I-AWM and I-CWM osmotic generators, generated in 100mM/1mM concentration gradient.

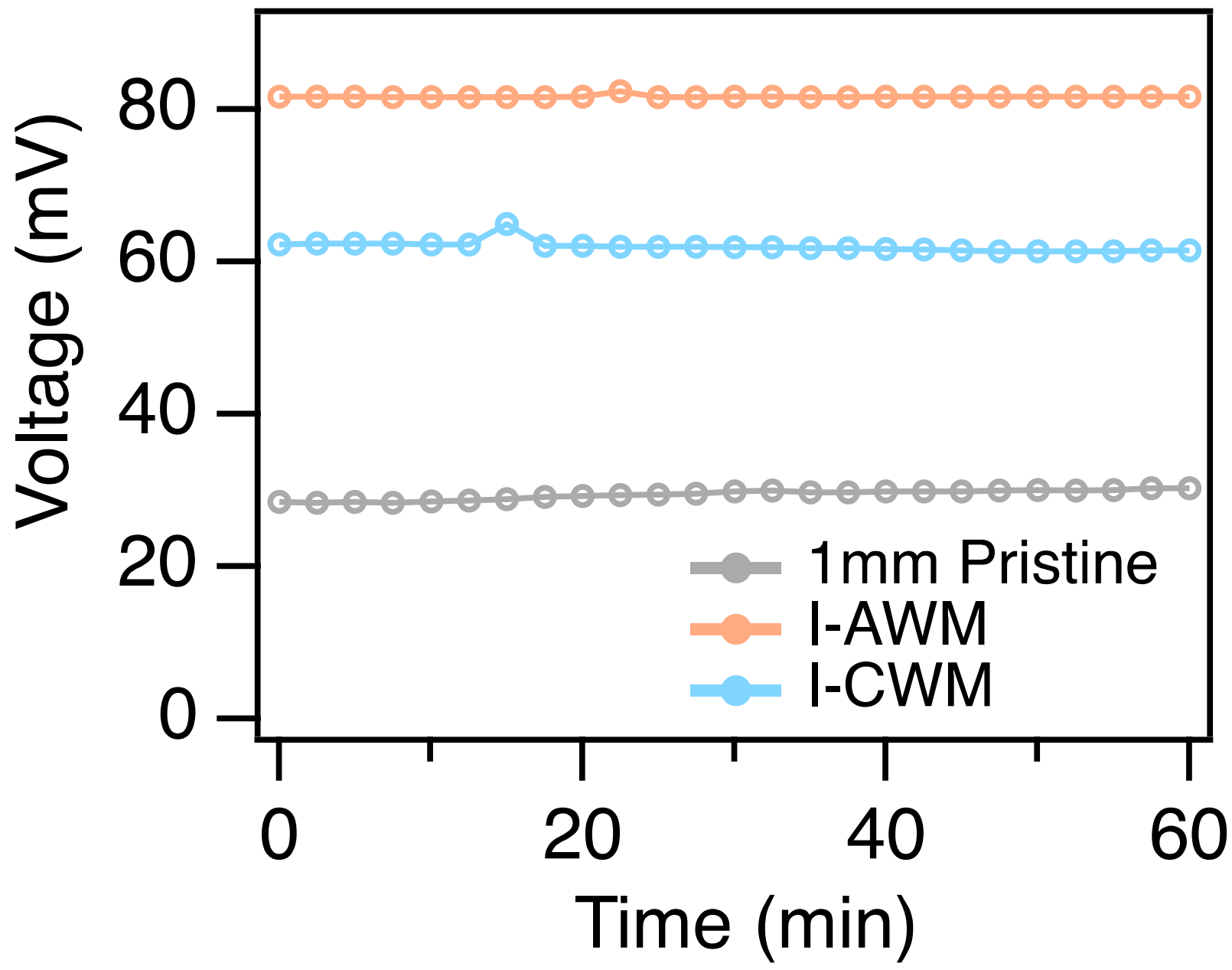

Figure S8. Open-circuit voltage comparison of 1 mm-thick pristine wood, I-AWM and I-CWM osmotic generators. V_{OC} of 1 mm-thick pristine wood, I-AWM and -CWM.

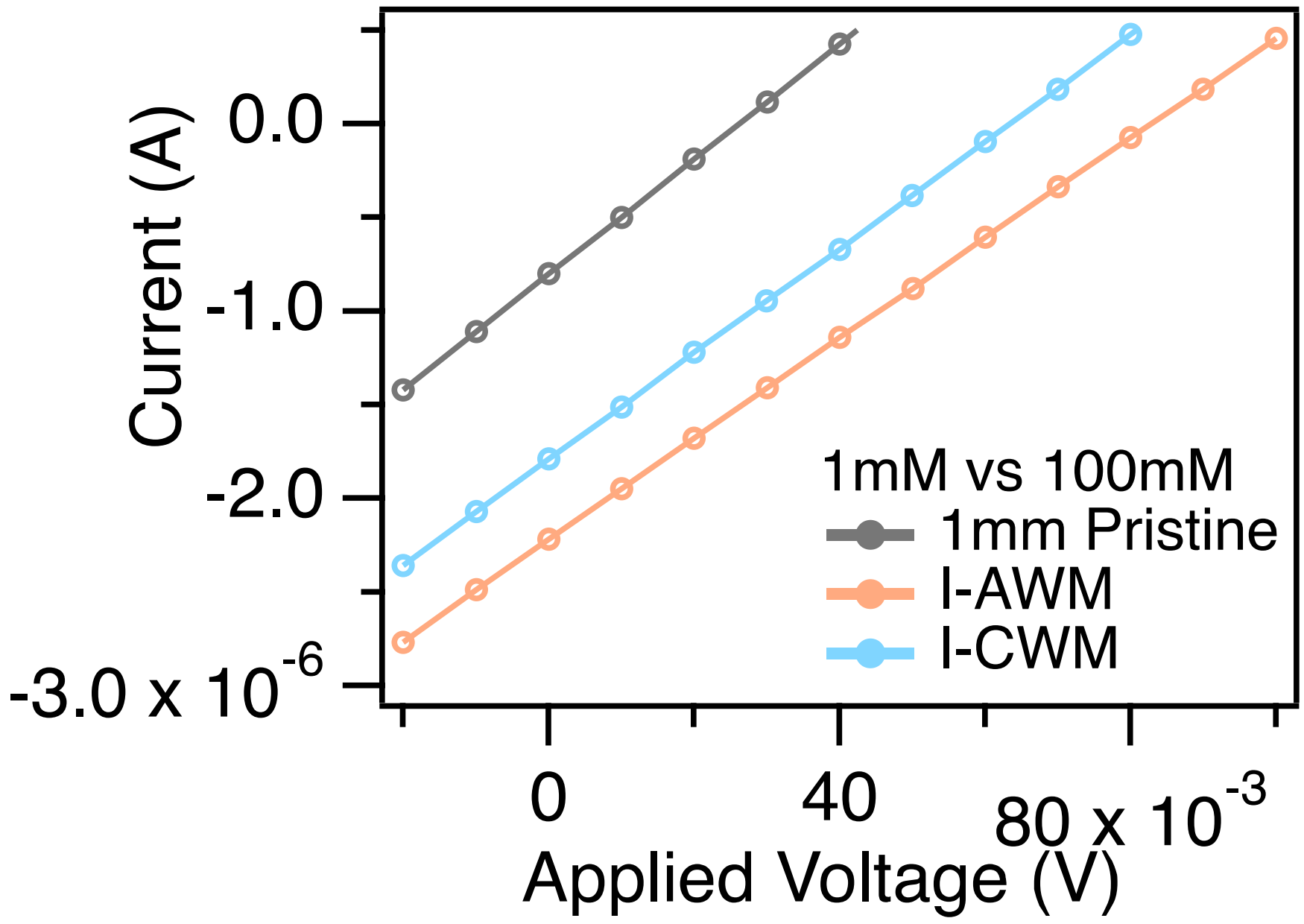

Figure S9. Comparative I-V performance of pristine wood, I-AWM and I-CWM at high concentration gradient. I-V curves obtained in 100mM/1mM concentration gradient.

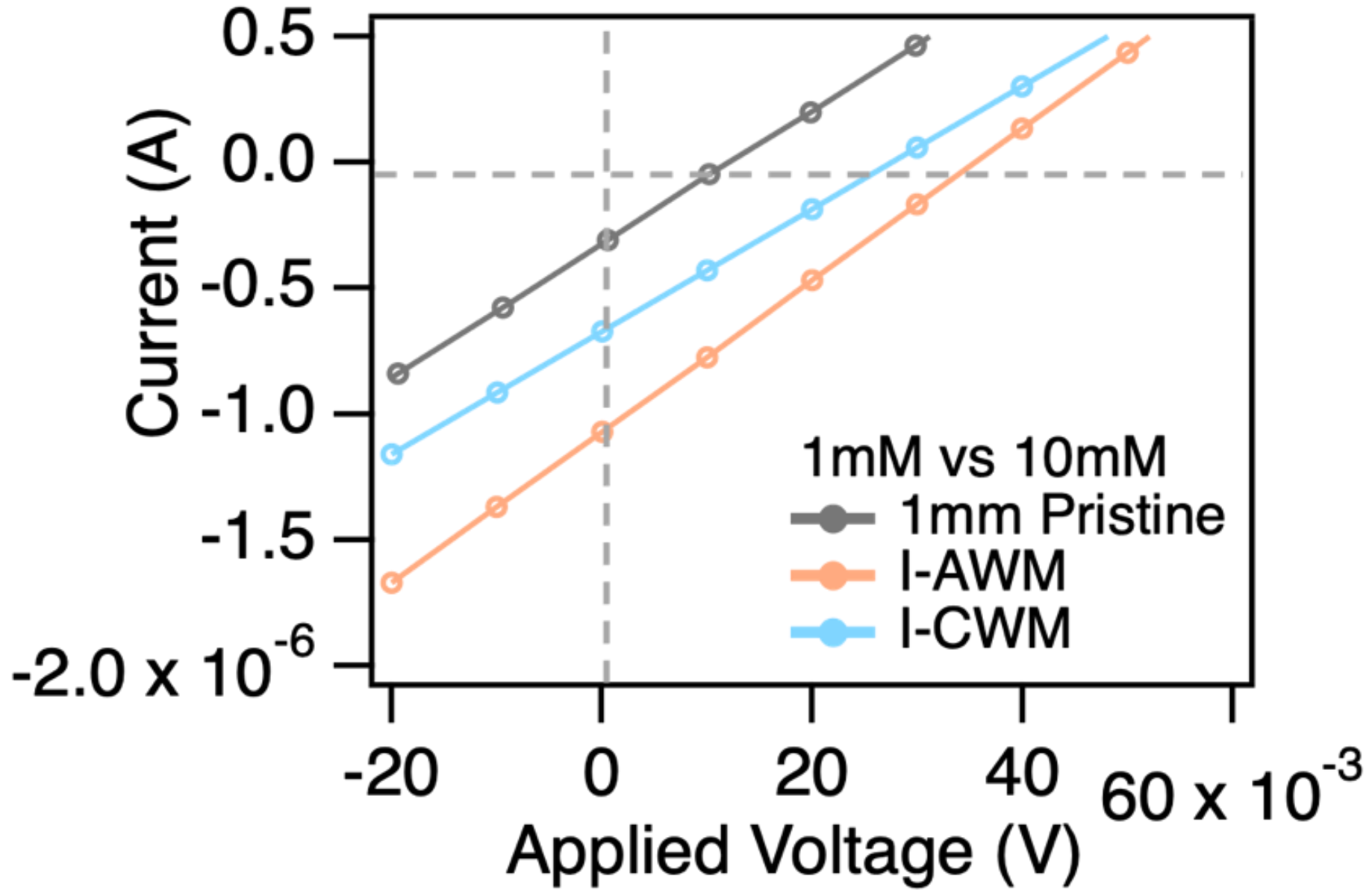

Figure S10. Comparative I-V performance of pristine wood, I-AWM and I-CWM at low concentration gradient. I-V curves obtained when 10 mM $Cu(NO_3)_2$ solution was placed in the left reservoir and 1 mM $Cu(NO_3)_2$ solution was added in the right reservoir.

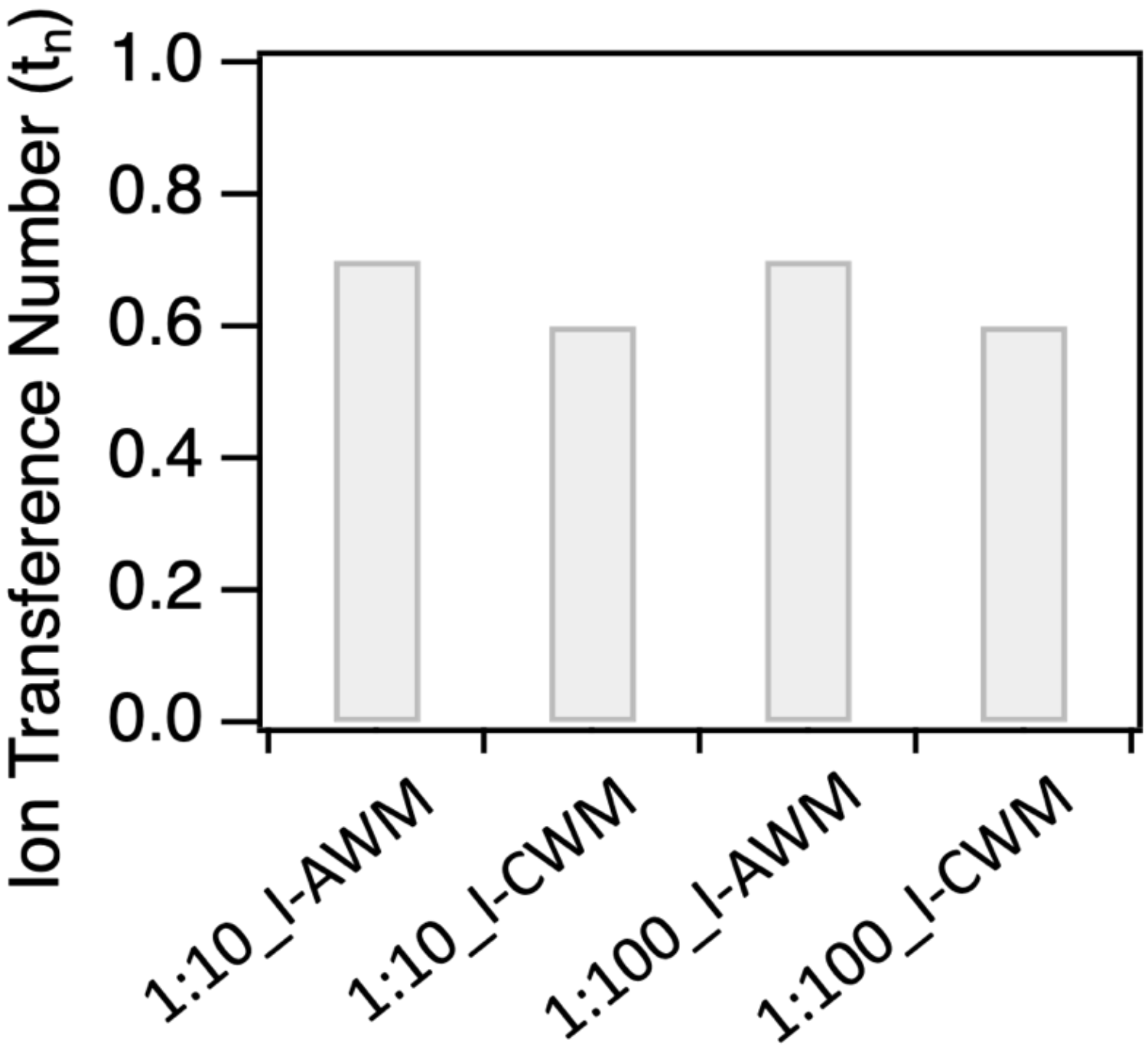

Figure S11. Ion selectivity evaluation of pristine wood and polyelectrolyte-encapsulated wood membranes. Plots of transference number.

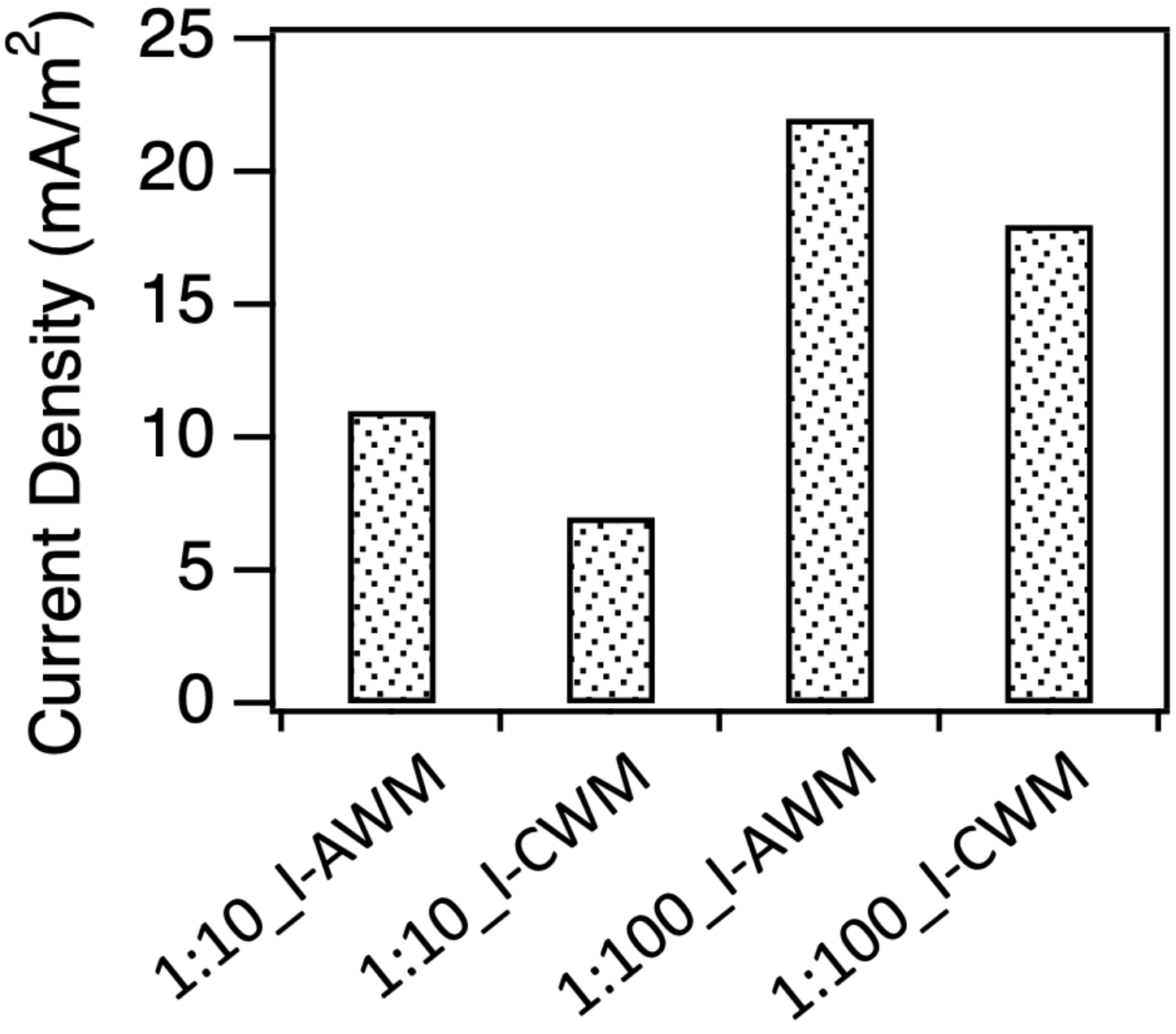

Figure S12. Comparison of osmotic energy conversion efficiency between surface- and interior-encapsulated wood membranes. Energy conversion efficiency at $Cu(NO_3)_2$ concentration gradient of 100mM/1mM and 10mM/1mM.

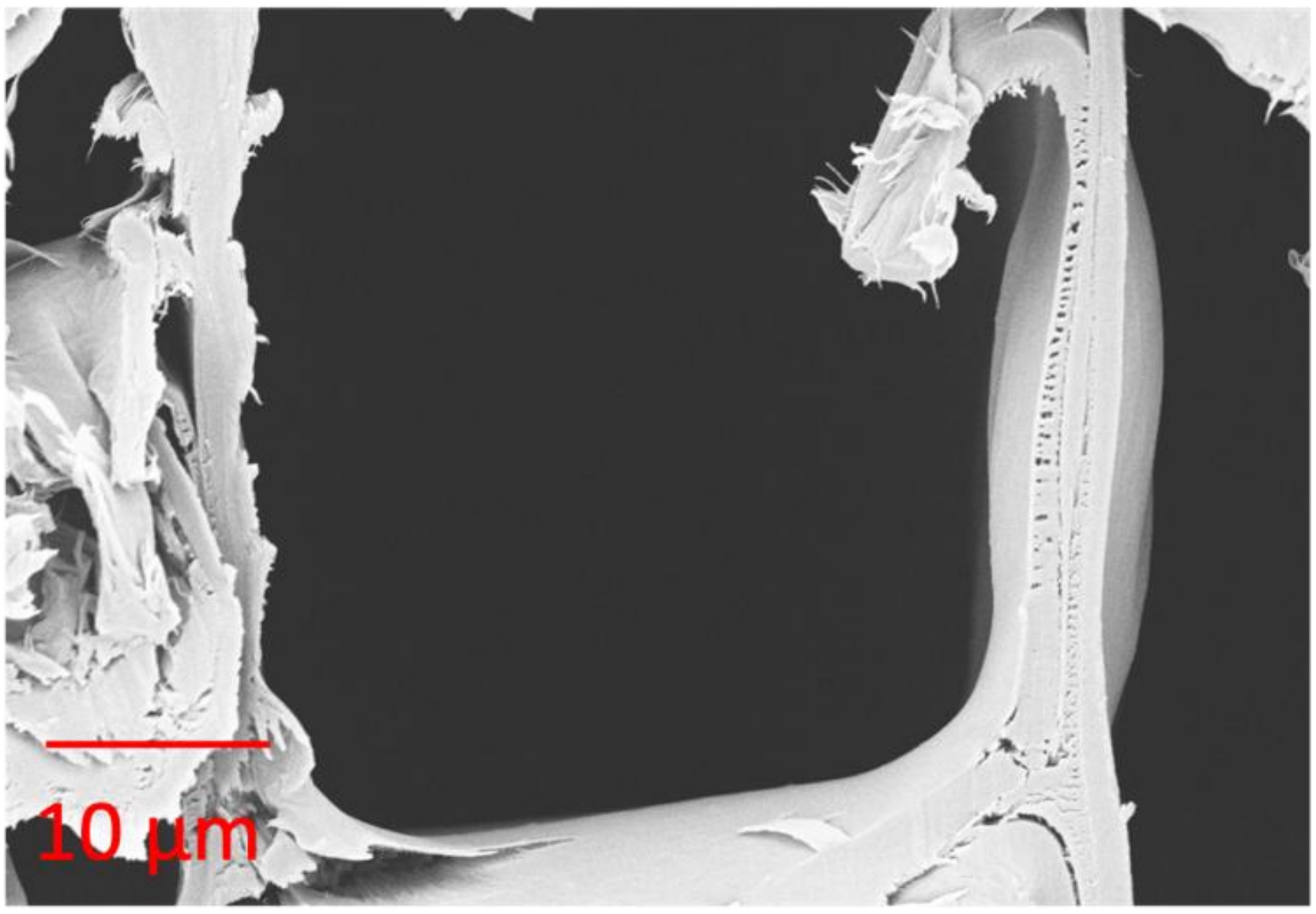

Figure S13. SEM images of the cross-sectional morphology of S-AWM (single cell). SEM images of S-AWM, one cell. Representative SEM images from 3 independent experiments with similar results.

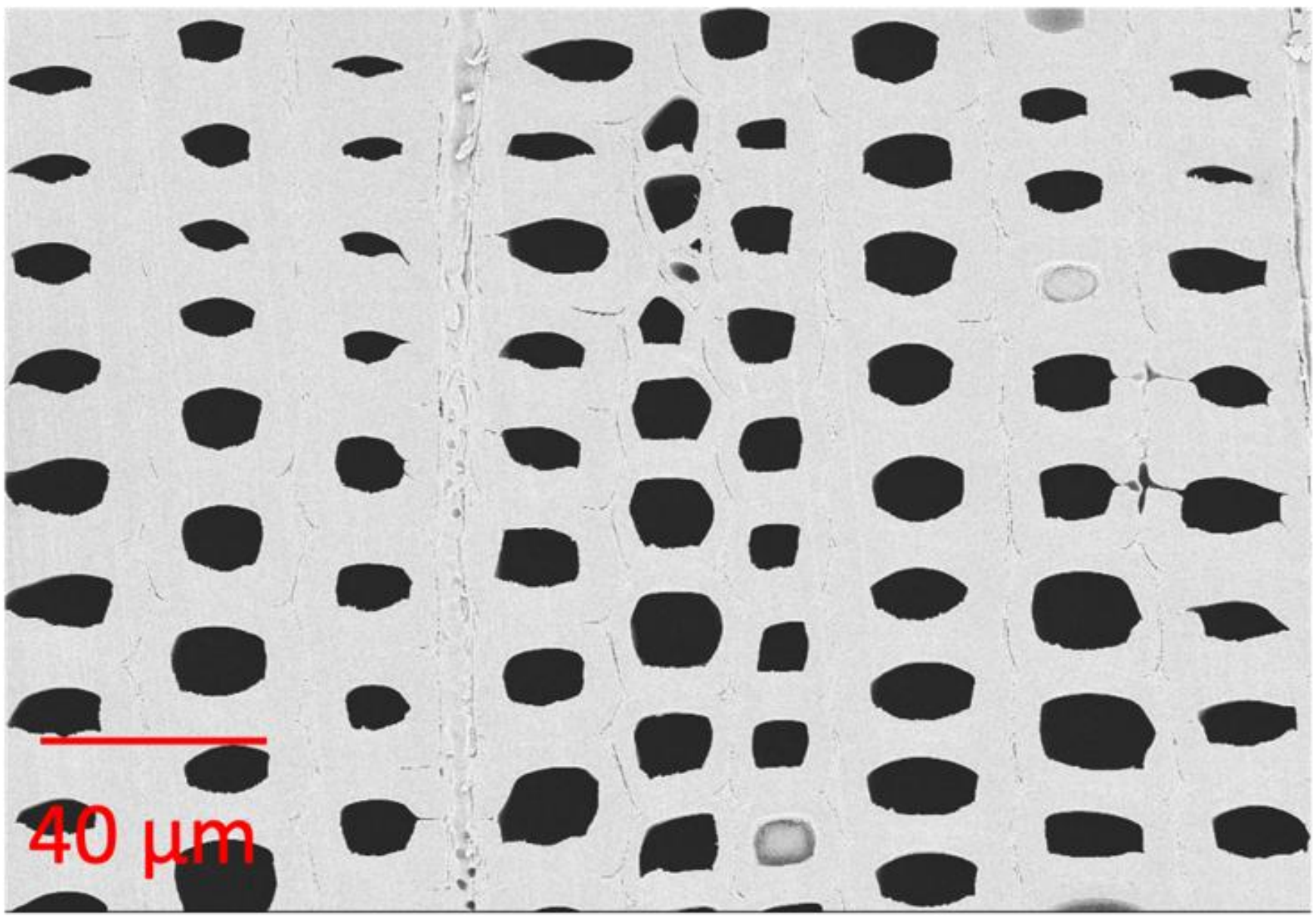

Figure S14. Cross-sectional SEM image of microtome-cut S-AWM at low magnification. Microtome-cut S-AWM, zoom out. Representative SEM images from 3 independent experiments with similar results.

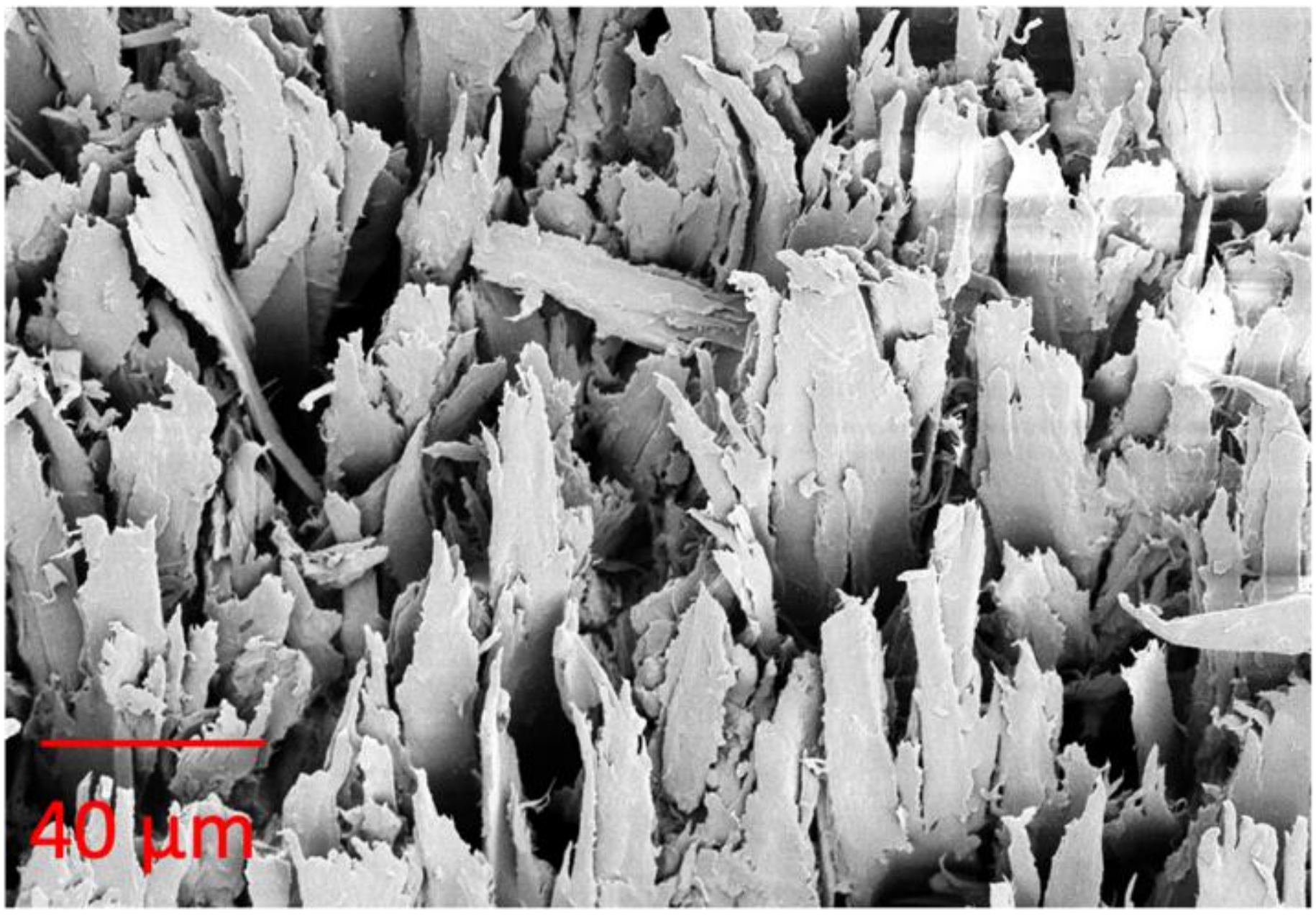

Figure S15. Cross-sectional SEM image of saw-cut S-AWM. Saw-cut S-AWM. Representative SEM images from 3 independent experiments with similar results.

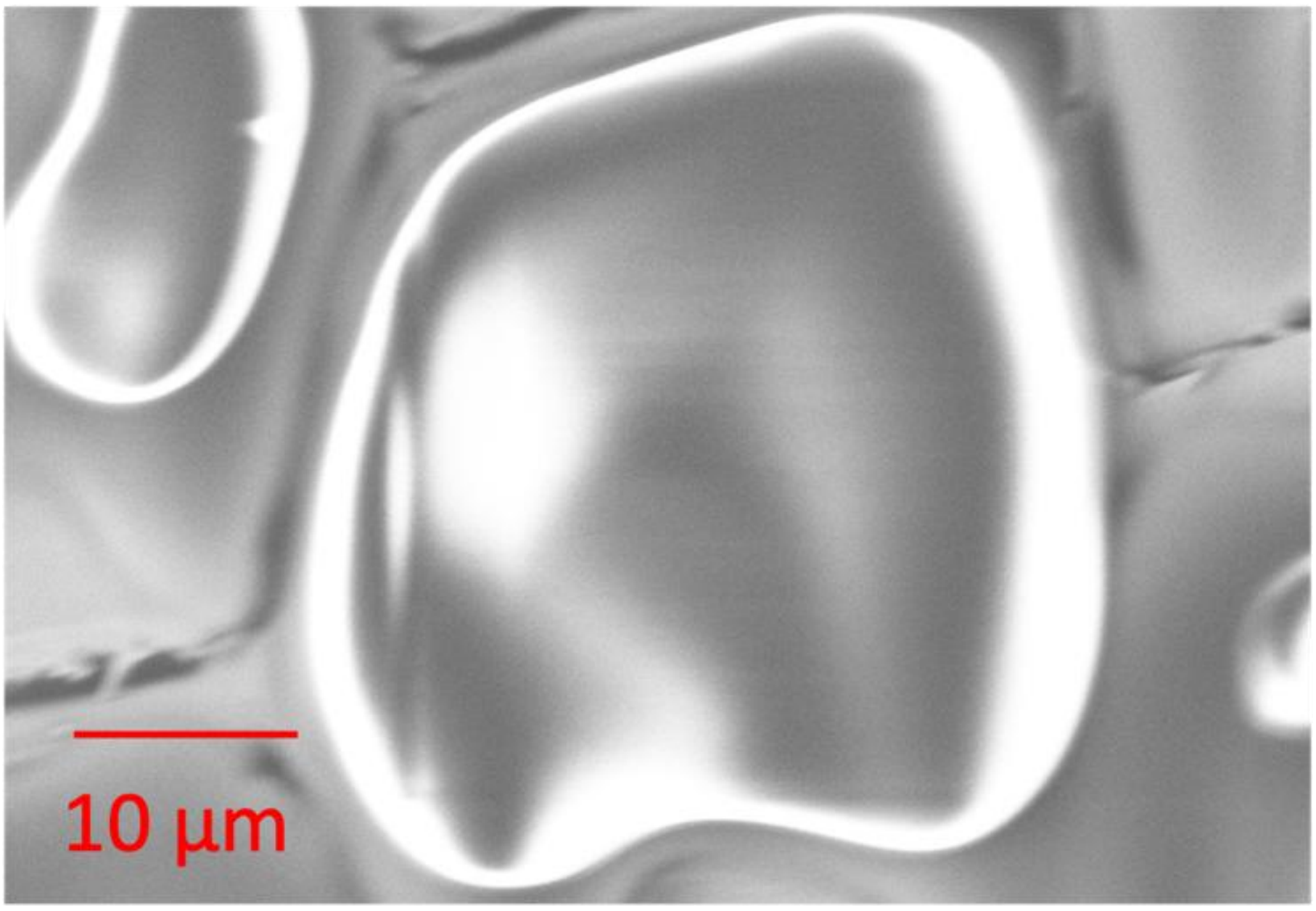

Figure S16. SEM images of the cross-sectional morphology of microtome-cut I-AWM (single cell). Microtome-cut I-AWM, one cell. Representative SEM images from 3 independent experiments with similar results.

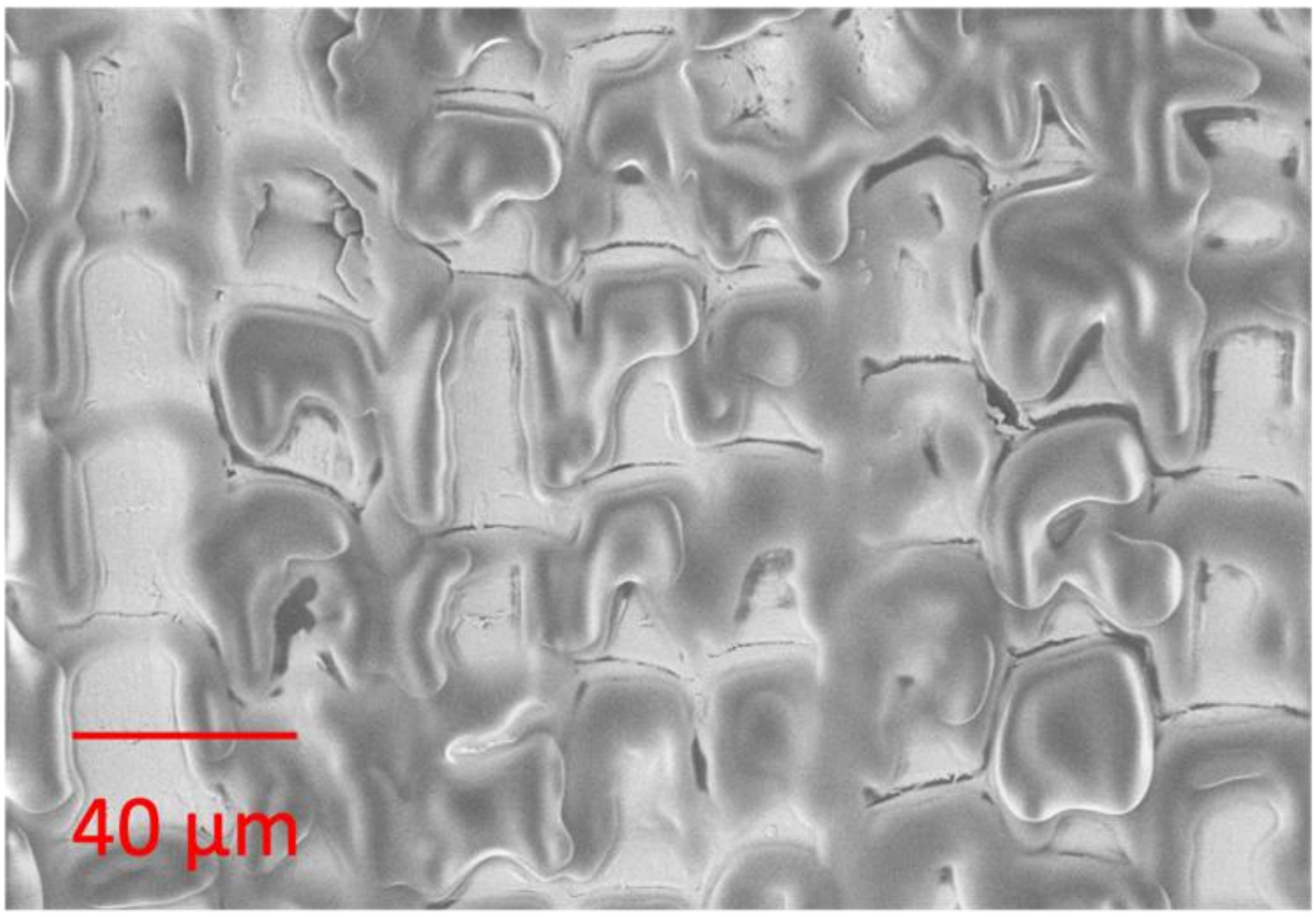

Figure S17. Cross-sectional SEM image of microtome-cut I-AWM at low magnification. Microtome-cut I-AWM, zoom out. Representative SEM images from 3 independent experiments with similar results.

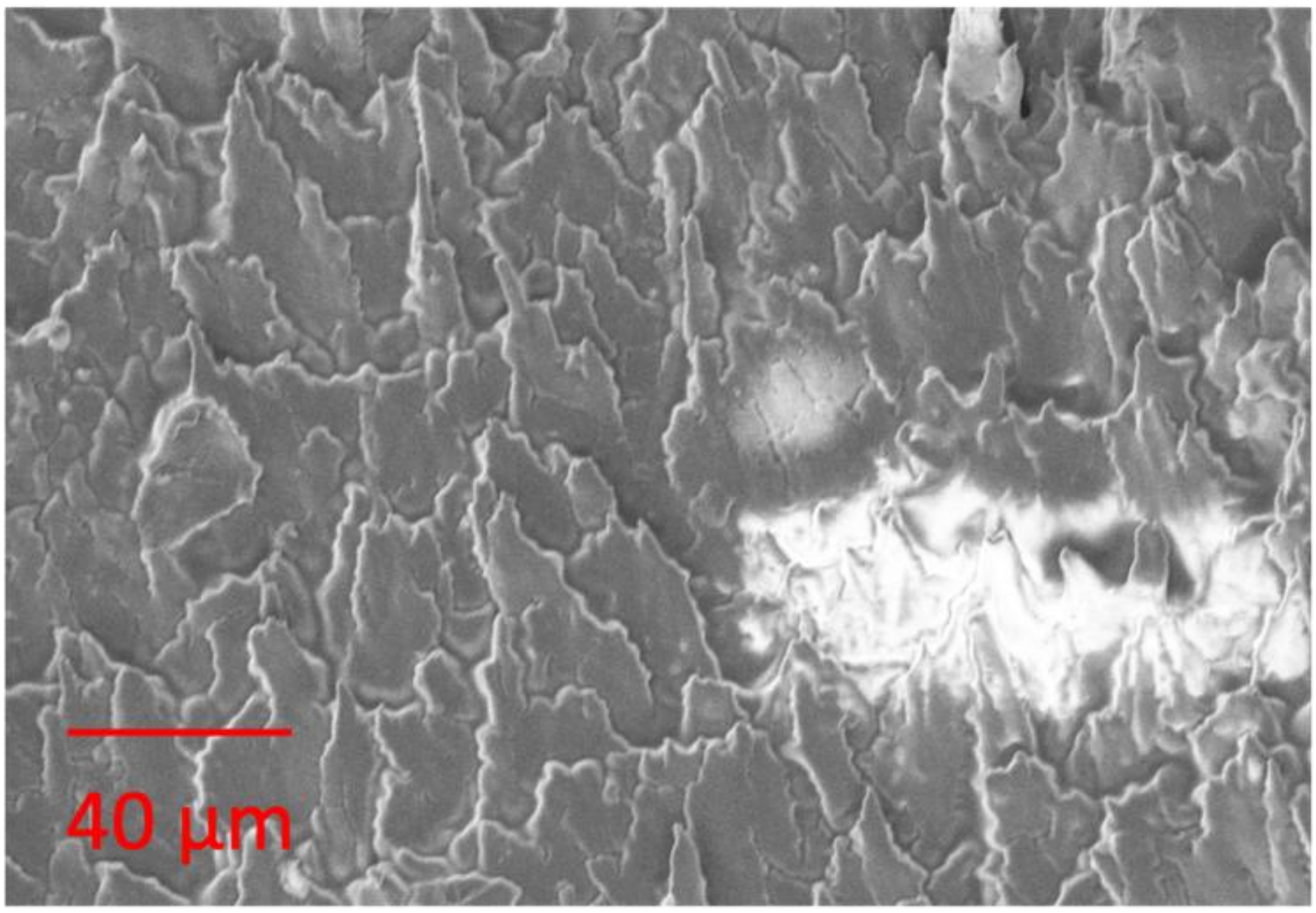

Figure S18. Cross-sectional SEM image of saw-cut I-AWM. Saw-cut I-AWM. Representative SEM images from 3 independent experiments with similar results.

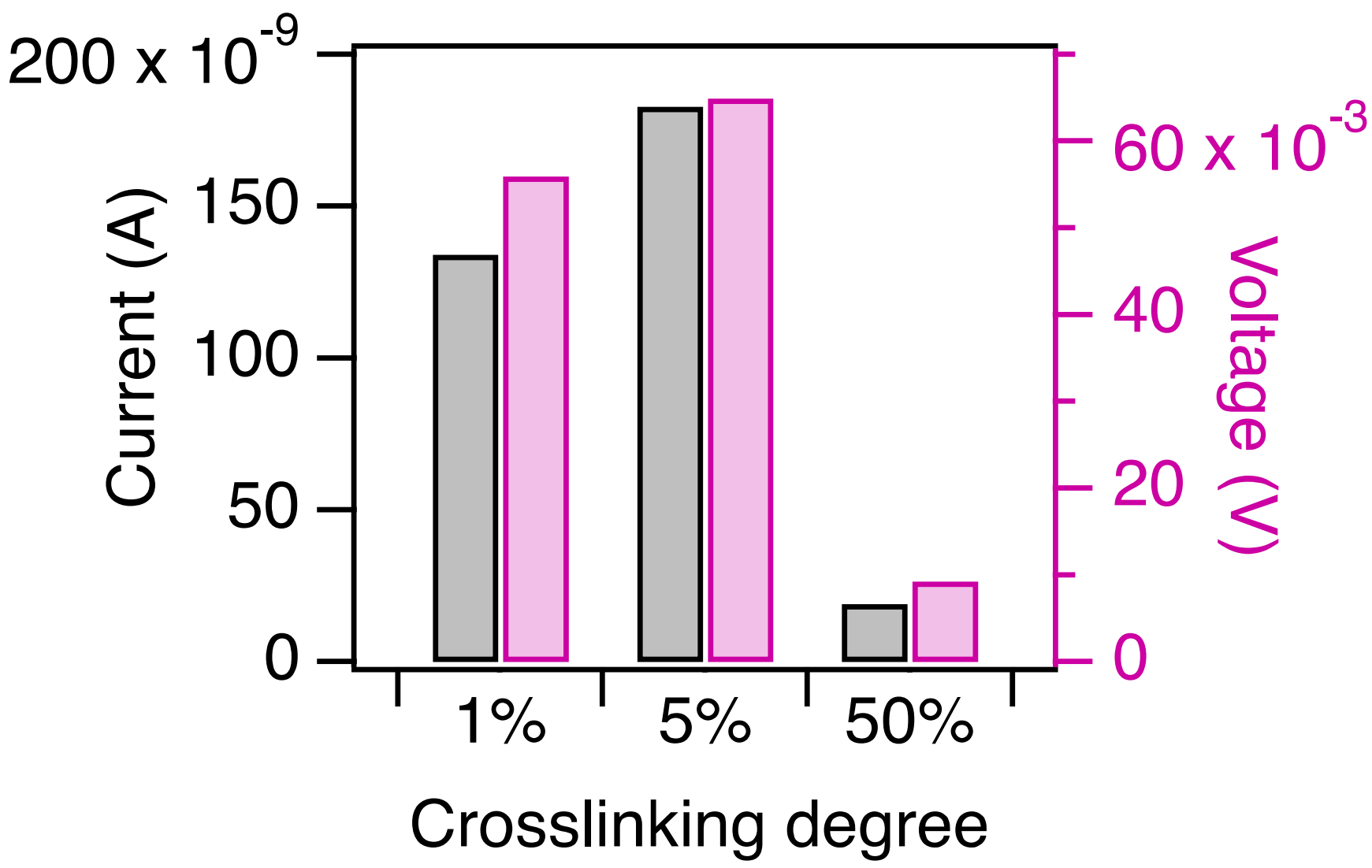

Figure S19. Effect of crosslinking degree on the osmotic energy conversion performance of S-AWM. I_{SC} and V_{OC} of S-AWM presenting different crosslinking degrees, generated in 100 mM/1 mM concentration gradient.

Table S1. Ion composition and concentration of simulated brine effluents from different industries used in osmotic energy harvesting experiments. The concentration of each characteristic brine solution was simulated as approximately the median value shown in Figure 4a.

Textile industry	Flue gas desulfurization (FGD)	Landfill leachate	Dairy industry	Aquaculture industry	Municipal wastewater	Pharmaceutical industry	Petrochemical industry
$CaCl_2$ 0.025 g<br>NaCl 0.140 g<br>$MgSO_4$ 0.061 g	$CaCl_2$ 0.206 g<br>NaCl 0.218 g<br>$MgSO_4$ 0.244 g	$CaCl_2$ 0.142 g<br>Na_2SO_4 0.152 g<br>NaCl 0.55 g	$CaCl_2$ 0.036 g<br>$MgCl_2$ 1.21 g	NaCl 0.25 g<br>Na_2SO_4 0.3 g	$CaCl_2$ 0.01 g<br>$MgSO_4$ 0.006 g<br>NaCl 0.022 g<br>$NaHCO_3$ 0.023 g<br>K_2SO_4 0.005 g	$CaCl_2$ 0.011 g<br>$MgSO_4$ 0.30 g<br>$MgCl_2$ 0.25 g	$CaCl_2$ 0.009 g<br>NaCl 0.026 g<br>Na_2HPO_4 0.005 g

Table S2 Structural, fabrication, and performance comparison of S-AWM/S-CWM against state-of-the-art synthetic and bio-based osmotic membranes

**Materials**	**Main Content**	**Membrane Structure & Scalability**	**Pre-treatment methods**	**Fabrication Process**	**External Energy Input**	**Normalized Power Density**	**Reference**
**S-AWM**	**98%Pristine wood + 2%Polymer**	**3D Bulk**	**None**	**Dip-coating + Sunlight**	**None**	**0.08 mW/m²**	**This work**
Ionized Wood Membrane	Delignified wood + Epoxy resin	3D Bulk	Delignification	Chemical oxidation of wood	Yes	5.14 mW/m²	[1]
Wood /sodium alginate	Delignified wood + Sodium alginate	3D Bulk	Delignification	SA infiltration	Yes	14.43 W/m²	[2]
Wood WEG	Delignified wood + Wood-derived carbon	3D Bulk	Delignification	Carbon coating	Yes	0.67 W/m²	[3]
Bark	Delignified wood bark	3D Bulk	Delignification	Densification	Yes	1.45 W/m²	[4]
ISWM PWM	Delignified wood	3D Bulk	Delignification	Hole engineering	Yes	0.658 W/m²	[5]
Dual-network fiber-hydrogel	Cellulose + Synthetic hydrogel	3D Bulk	Synthetic copolymerization	Cross-linking	Yes	6.75 W/m²	[6]
Reed membrane	Natural reed	3D Bulk	Physical cutting	Chemical activation	Yes	2.6 W/m²	[7]
KL/TCNF-PEDOT:PSS	79% TCNF + 15% Kraft Lignin	2D Film	Lignin/Cellulose extraction & mixing	Vacuum filtration	Yes	1.37 W/m²	[8]
KCNF/MXene	40% Kelp CNF	2D Film	Acid exfoliation & mixing	Vacuum filtration	Yes	10.56 W/m²	[9]
2D-HM	Chitin nanosheets	2D Film	Chemical exfoliation	Vacuum filtration	Yes	2.59 W/m²	[10]
CSs Membrane	Chitin sub-nanosheets	2D Film	Chemical exfoliation	Vacuum filtration	Yes	8.31 W/m²	[11]
CNFs/CNTs	~ 50% Cellulose Nanofibers	2D Film	Chemical extraction & mixing	Vacuum filtration	Yes	4.67 W/m²	[12]

KANF	Synthetic Kevlar aramid	2D Film	Kevlar synthetic processing	Vacuum filtration	Yes	4.8 W/m^2	[13]
**Materials**	**Main Content**	**Membrane Structure & Scalability**	**Pre-treatment methods**	**Fabrication Process**	**External Energy Input**	**Normalized Power Density**	**Reference**
C-MXene/C-HNF	20% Holocellulose HNF	2D Film	Chemical exfoliation & mixing	Vacuum filtration	Yes	1.09 W/m^2	[14]
Natural cellulose n-membrane	Natural cellulose	2D Film	Chemical extraction	Vacuum filtration	Yes	2.6 W/m^2	[15]
A-CNF	Modified cellulose	2D Film	Chemical modification	Vacuum filtration	Yes	0.81 W/m^2	[16]
C-CNF	Modified cellulose	2D Film	Chemical modification	Vacuum filtration	Yes	1.4 W/m^2	[16]
PBONM	Synthetic PBO polymer	2D Film	Toxic solvent dissolution	Electrospinning & Filtration	Yes	7.7 W/m^2	[17]
PNIPAM-g-sCC	~ 40% Cellulose core	2D Film	Graft copolymerization	Vacuum filtration	Yes	10.1 W/m^2	[18]
RCNF	Regenerated cellulose	2D Film	Cellulose dissolution	Wet-spinning & Regeneration	Yes	2.57 W/m^2	[19]

Supplementary References

1 Wu, Q. Y. *et al.* Salinity-gradient power generation with ionized wood membranes. *Adv. Energy Mater.* **10**, 1902590 (2020).

2 Zhou, T. *et al.* Leveraging Natural Wood Structures for Sustainable and High-Performance Osmotic Energy Harvesting. *Small*, e13371 (2026).

3 Zhang, K. *et al.* All-wood-based ionic power generator with dual functions for alkaline wastewater reuse and energy harvesting. *Acs Nano* **18**, 10259-10269 (2024).

4 Song, G. *et al.* Self-adhesive ionic cable derived from natural bark as osmotic energy generator. *Nano Energy* **131**, 110326 (2024).

5 Yang, T. *et al.* Advanced ion-selective wood membranes: Leveraging aligned cellulose nanofibers for enhanced osmotic energy conversion. *Chem. Eng. J.* **496**, 153891 (2024).

6 Cao, L. & Wu, H. Dual-network fiber-hydrogel membrane for osmotic energy harvesting. *Frontiers in Chemistry* **12**, 1401854 (2024).

7 Zheng, X. *et al.* Renewable, All-Natural Flute Membrane with Excellent Mechanical Properties for Osmotic Energy Harvesting. *ACS Appl. Mater. Interfaces* **16**, 70618-70625 (2024).

8 Xu, A., Qi, S., Ahmad, M. & Wang, S. Industrial Kraft Lignin-Functionalized Cellulose Nanofluidic Membrane for Enhanced Osmotic Energy Conversion. *ACS Applied Nano Materials* **8**, 2974-2985 (2025).

9 Zhai, R. *et al.* Kelp Nanofiber-Based Composite Membranes for Highly Efficient Osmotic Energy Conversion. *Adv. Funct. Mater.* **34**, 2313914 (2024).

10 Xiang, Z. *et al.* Sustainable Chitin-Derived 2D Nanosheets with Hierarchical Ion Transport for Osmotic Energy Harvesting. *Adv. Energy Mater.* **14**, 2402304 (2024).

11 Shu, Y. *et al.* 2D Chitin Sub-Nanosheets With Extreme Ion Transport for Nanofluidic Sensing. *Adv. Mater.* **38**, e10095 (2026).

12 Li, X., Xiao, T., Lu, B., He, J. & Zhai, J. Double-network ion channels for high-performance osmotic power generation. *Adv. Mater. Interfaces* **9**, 2101960 (2022).

13 Ding, L. *et al.* Ultrathin and ultrastrong kevlar aramid nanofiber membranes for highly stable osmotic energy conversion. *Adv. Sci.* **9**, 2202869 (2022).

14 Rao, J. *et al.* Nacre-Inspired Mechanically Robust Films for Osmotic Energy Conversion. *Adv. Funct. Mater.* **34**, 2309869 (2024).

15 Chen, C. *et al.* Nanofluidic osmotic power generation from CO2 with cellulose membranes. *Green Carbon* **1**, 58-64 (2023).

16 Wang, S., Sun, Z., Ahmad, M., Fu, W. & Gao, Z. Engineered cellulose nanofibers membranes with oppositely charge characteristics for high-performance salinity gradient power generation by reverse electrodialysis. *Int. J. Biol. Macromol.* **253**, 126608 (2023).

17 Duan, R. *et al.* High-strength, thin PBO nanofiber membrane with long-term stability for osmotic energy conversion. *Adv. Funct. Mater.* **34**, 2311258 (2024).

18 Lin, X. *et al.* Temperature-gated nanocellulose membrane for enhanced and controllable osmotic energy harvesting. *Nano Energy* **107**, 108156 (2023).

19 Zhou, B. *et al.* Aligned regenerated cellulose-based nanofluidic fibers with ultrahigh ionic conductivity and underwater stability for osmotic energy harvesting. *Chem. Eng. J.* **457**, 141167 (2023).
