# Supplementary material for: Continuous electricity from charged total dissolved solids in wastewater using a wood-based ion-selective power generator
Source: Nat Commun. 2026 Jul 9;17:8636. doi: 10.1038/s41467-026-75514-7 (PMC13486703; doi:10.1038/s41467-026-75514-7)
Supplement: Supplementary file 2 [file 41467_2026_75514_MOESM2_ESM.pdf]

Corresponding author(s): Shihang Li; Kunkun Tu

Last updated by author(s): Apr 1, 2026

Reporting Summary

Nature Portfolio wishes to improve the reproducibility of the work that we publish. This form provides structure for consistency and transparency in reporting. For further information on Nature Portfolio policies, see our Editorial Policies and the Editorial Policy Checklist.

Statistics

For all statistical analyses, confirm that the following items are present in the figure legend, table legend, main text, or Methods section.

n/a	Confirmed	
☐	☒	The exact sample size (*n*) for each experimental group/condition, given as a discrete number and unit of measurement
☐	☒	A statement on whether measurements were taken from distinct samples or whether the same sample was measured repeatedly
☒	☐	The statistical test(s) used AND whether they are one- or two-sided<br>*Only common tests should be described solely by name; describe more complex techniques in the Methods section.*
☒	☐	A description of all covariates tested
☒	☐	A description of any assumptions or corrections, such as tests of normality and adjustment for multiple comparisons
☐	☒	A full description of the statistical parameters including central tendency (e.g. means) or other basic estimates (e.g. regression coefficient) AND variation (e.g. standard deviation) or associated estimates of uncertainty (e.g. confidence intervals)
☒	☐	For null hypothesis testing, the test statistic (e.g. *F*, *t*, *r*) with confidence intervals, effect sizes, degrees of freedom and *P* value noted<br>*Give P values as exact values whenever suitable.*
☒	☐	For Bayesian analysis, information on the choice of priors and Markov chain Monte Carlo settings
☒	☐	For hierarchical and complex designs, identification of the appropriate level for tests and full reporting of outcomes
☒	☐	Estimates of effect sizes (e.g. Cohen's *d*, Pearson's *r*), indicating how they were calculated

Our web collection on statistics for biologists contains articles on many of the points above.

Software and code

Policy information about availability of computer code

Data collection	Data were collected using the standard operating software provided with the respective instruments (e.g., Gamry Framework for electrochemical tests, Bruker OPUS for FTIR
Data analysis	Origin 2021

For manuscripts utilizing custom algorithms or software that are central to the research but not yet described in published literature, software must be made available to editors and reviewers. We strongly encourage code deposition in a community repository (e.g. GitHub). See the Nature Portfolio guidelines for submitting code & software for further information.

Data

Policy information about availability of data

All manuscripts must include a data availability statement. This statement should provide the following information, where applicable:

- Accession codes, unique identifiers, or web links for publicly available datasets
- A description of any restrictions on data availability
- For clinical datasets or third party data, please ensure that the statement adheres to our policy

All data needed to evaluate the conclusions in the paper are present in the paper and/or the Supplementary Materials. Source data are provided with this paper. Additional data related to this paper may be requested from the authors.

Research involving human participants, their data, or biological material

Policy information about studies with human participants or human data. See also policy information about sex, gender (identity/presentation), and sexual orientation and race, ethnicity and racism.

Reporting on sex and gender	This study does not involve human participants, their data, or biological material.
Reporting on race, ethnicity, or other socially relevant groupings	Not applicable.
Population characteristics	Not applicable.
Recruitment	Not applicable.
Ethics oversight	Not applicable.

Note that full information on the approval of the study protocol must also be provided in the manuscript.

Field-specific reporting

Please select the one below that is the best fit for your research. If you are not sure, read the appropriate sections before making your selection.

☐ Life sciences ☐ Behavioural & social sciences ☒ Ecological, evolutionary & environmental sciences

For a reference copy of the document with all sections, see nature.com/documents/nr-reporting-summary-flat.pdf

Ecological, evolutionary & environmental sciences study design

All studies must disclose on these points even when the disclosure is negative.

Study description	This study fabricated surface-encapsulated anion-selective (S-AWM) and cation-selective (S-CWM) wood membranes via sunlight-induced polymerization for continuous electricity generation from wastewater. The quantitative factors investigated included membrane thickness (1 mm, 3 mm, 10 mm) , salt concentration gradients , pH levels (pH 1-6) , polyelectrolyte crosslinking degrees (1%, 5%, 50%) , and various simulated industrial wastewaters. Standard deviations for key metrics (e.g., weight increase) were calculated from three independent replicate samples.
Research sample	The primary research samples were native Norway spruce (Picea abies) wood cross-sections cut into specific dimensions (e.g., 20 × 20 × 3 mm^3). Wood was selected as the substrate because its inherent mechanical rigidity reinforces the mechanical strength of the resulting polyelectrolyte hydrogel, and its unique hierarchically porous structure provides a high specific surface area for rapid ion/electron transportation. Modulated industrial wastewaters were formulated based on median ion concentrations sourced from existing literature data.
Sampling strategy	No formal statistical sample-size pre-calculation was performed. In line with standard practices in materials science and electrochemistry, at least three independent sample replicates were tested for quantitative material characterizations (such as weight increment calculations). This sample size is sufficient to ensure basic reproducibility and to identify consistent trends in electrochemical performance without causing excessive material waste.
Data collection	Data were collected using standardized instrumentation. Chemical structures were analyzed using an ATR-FTIR spectrometer (Bruker Tensor 27). Microscopic morphologies were recorded via Scanning Electron Microscopy (Thermo Scientific, Ultradry). Electrochemical data, including open-circuit voltage (VOC) and short-circuit current (ISC), were continuously recorded by an electrochemical workstation (Gamry Interface 1010E) using standard Ag/AgCl electrodes in H-type electrolytic tanks.
Timing and spatial scale	The study was conducted at a laboratory scale, with single-cell active testing areas covering approximately 1 cm^2. The real-world sunlight-initiated photopolymerization sample was specifically prepared in Zurich, Switzerland, from 8:00 to 20:00 on August 4, 2022. Electrochemical continuous stability tests were recorded over varied periods ranging from 60 minutes up to extended periods of 1000 and 4000 minutes to assess long-term operational robustness.
Data exclusions	No data were excluded from the analyses.
Reproducibility	Experimental findings were highly reproducible. Key energy conversion performances were verified using independent repeat samples synthesized under identical conditions (labeled as "1st", "2nd", and "3rd" samples). All attempts to repeat the experiments were successful, and uniform trends were consistently observed across all replicates.
Randomization	Randomization is not highly relevant to this study. This is a materials engineering study where wood samples were cut from the same native batches and deliberately allocated into specific experimental groups based on the targeted chemical modification (e.g., DMAEMA monomer for S-AWM vs. AA monomer for S-CWM) or precise dimensional variables (e.g., 1mm vs. 3mm thickness). Covariates were controlled by maintaining identical testing environments and standardizing the substrate species.

Blinding	Blinding was not relevant to this study. Researchers needed to know the specific treatment conditions, modifications, and testing parameters (e.g., identifying whether a membrane was anion- or cation-selective, or what type of simulated wastewater was in the chamber) to conduct the appropriate characterizations and accurately assess the mechanisms.

Did the study involve field work? ☐ Yes ☒ No

Reporting for specific materials, systems and methods

We require information from authors about some types of materials, experimental systems and methods used in many studies. Here, indicate whether each material, system or method listed is relevant to your study. If you are not sure if a list item applies to your research, read the appropriate section before selecting a response.

Materials & experimental systems

n/a	Involved in the study
☒	☐ Antibodies
☒	☐ Eukaryotic cell lines
☒	☐ Palaeontology and archaeology
☒	☐ Animals and other organisms
☒	☐ Clinical data
☒	☐ Dual use research of concern
☐	☒ Plants

Methods

n/a	Involved in the study
☒	☐ ChIP-seq
☒	☐ Flow cytometry
☒	☐ MRI-based neuroimaging

Dual use research of concern

Policy information about dual use research of concern

Hazards

Could the accidental, deliberate or reckless misuse of agents or technologies generated in the work, or the application of information presented in the manuscript, pose a threat to:

No	Yes
☒	☐ Public health
☒	☐ National security
☒	☐ Crops and/or livestock
☒	☐ Ecosystems
☒	☐ Any other significant area

Experiments of concern

Does the work involve any of these experiments of concern:

No	Yes
☒	☐ Demonstrate how to render a vaccine ineffective
☒	☐ Confer resistance to therapeutically useful antibiotics or antiviral agents
☒	☐ Enhance the virulence of a pathogen or render a nonpathogen virulent
☒	☐ Increase transmissibility of a pathogen
☒	☐ Alter the host range of a pathogen
☒	☐ Enable evasion of diagnostic/detection modalities
☒	☐ Enable the weaponization of a biological agent or toxin
☒	☐ Any other potentially harmful combination of experiments and agents

Plants

Seed stocks	Native wood species, Norway spruce (Picea abies), were used. All the wood materials are readily available from the authors or from standard commercial sources.
Novel plant genotypes	N/A. This study does not involve the production of novel plant genotypes or transgenic plants.
Authentication	N/A. Standard native wood materials were used; no specific genetic authentication was required.
