# Supplementary material for: Continuous electricity from charged total dissolved solids in wastewater using a wood-based ion-selective power generator
Source: Nat Commun. 2026 Jul 9;17:8636. doi: 10.1038/s41467-026-75514-7 (PMC13486703; doi:10.1038/s41467-026-75514-7)
Supplement: Supplementary file 3 [file 41467_2026_75514_MOESM3_ESM.pdf]

nature portfolio

Peer Review File

Continuous Electricity Generation from Charged Total Dissolved Solids in Wastewater Using a Wood-based Ion-Selective Power Generator

Corresponding Author: Dr Kun Tu

This file contains all reviewer reports in order by version, followed by all author rebuttals in order by version.

Version 0:

Reviewer comments:

Reviewer #1

(Remarks to the Author)
This study proposes an innovative surface encapsulation strategy to fabricate ion-selective wood membranes (S-AWM/S-CWM) with high bio-based content (~98 wt%) and successfully demonstrates their capability to generate electricity from wastewater salinity gradients. This work's core innovation, leveraging wood's natural structure for selective surface modification to avoid pore filling and employing sunlight-induced polymerization, stands out notably. It offers a novel approach for developing low-cost, sustainable osmotic energy harvesting devices. The paper is supported by solid data and comprehensive characterization throughout. However, there is room for improvement regarding the depth of mechanistic explanation and long-term stability in real wastewater systems. I therefore recommend minor revision before the work can be considered for publication.
Below are my specific questions and suggestions.
1. When the system treats wastewater, do electrons from the wastewater participate in the electrode reactions? If ions act as reactants in the electrode reactions that generate current, it might lead to adverse effects such as metal deposition or corrosion on electrical devices.
2. Regarding the point on page 11 noting "improved performance but decreased stability at low pH," please elaborate further in the discussion. It must be clearly stated that this limits the direct long-term application of this membrane in strongly acidic wastewater, and potential solutions (such as pre-treatment or pH buffering) should be proposed.
3. The term "modulated wastewater" is used multiple times in the text. It is recommended to clearly specify the exact source (e.g., citing literature) and rationale for selecting its simulated formulation on page 15 or in the methods section, to strengthen the representativeness and reproducibility of the experimental design.
4. Please review and standardize the format of units throughout the entire text (e.g., ensure the use of "μm" instead of "um").
5. Please check the grammar and spelling throughout the full text. For example, on page 3/line 91, "wood' inherent" should be "wood's inherent."

Reviewer #2

(Remarks to the Author)
The manuscript presents an interesting concept by combining bio-based materials with osmotic energy harvesting from wastewater, and the idea of using wood as a hierarchical scaffold for ion-selective transport is appealing from both sustainability and materials engineering perspectives. The work is clearly motivated by circular economy considerations and demonstrates a creative attempt to integrate wastewater utilization with energy recovery. However, in its current form, the study requires major revision before it can be considered for publication in Nature Communications, primarily because several key aspects related to scientific depth, practical relevance, and validation remain insufficiently addressed.

- The central claim of scalability and practical applicability needs to be supported more convincingly. While the manuscript shows that multiple cells connected in series can increase voltage output and power small electronic devices, the demonstrated performance remains at a laboratory scale, and the pathway toward realistic scale-up is not fully discussed. For example, it is not clear how performance would evolve when moving from centimeter-scale wood blocks to module-scale assemblies where internal resistance, concentration polarization, and hydraulic management become dominant limitations. A quantitative discussion of power density, projected module footprint, and comparison with existing osmotic energy

technologies would significantly strengthen the impact. At present, the scale-up argument is largely conceptual rather than engineering-driven.

- The stability assessment is another major limitation. The electrical performance is mainly evaluated over relatively short durations, typically on the order of tens of minutes to one hour, as shown in the stability plots where current and voltage are monitored over about 60 minutes. This is not sufficient to support claims of continuous electricity generation or industrial relevance. Long-term stability under realistic operating conditions, including cyclic salinity gradients, continuous operation over days or weeks, and exposure to real wastewater containing organic matter or fouling species, is necessary. In particular, wood-based substrates may undergo swelling, microbial degradation, or structural fatigue in aqueous environments, and these aspects are not addressed. It would also be important to understand whether the polyelectrolyte layer leaches or degrades during operation, especially under varying pH or ionic strength conditions.

- The manuscript would also benefit from clearer benchmarking against established ion-selective membranes and osmotic energy systems. The reported voltages and currents are compared primarily with pristine wood rather than with state-of-the-art osmotic power membranes such as nanofluidic membranes, ion-exchange membranes, or other bio-based systems. Without such benchmarking, it is difficult to assess whether the observed performance represents a meaningful advance or mainly demonstrates proof-of-concept functionality. Including normalized metrics such as power density per membrane area and comparing them with literature values would help clarify the significance of the results.

- The mechanistic discussion of ion selectivity could also be strengthened. The work attributes improved performance to surface-encapsulated polyelectrolytes and hierarchical wood channels, but quantitative characterization of charge density, effective pore size, or transport selectivity is limited. For example, transference number measurements are shown, but the link between polymer distribution, crosslinking degree, and ion transport efficiency remains largely qualitative. A deeper structure–transport relationship analysis would make the study more compelling for a high-impact audience.

- Another point that requires clarification is the durability of the device under varying chemical environments. The manuscript itself notes reduced stability at low pH due to corrosion effects, which raises concerns about robustness in industrial wastewater streams where pH and ionic composition fluctuate. Demonstrating chemical stability, mechanical integrity, and performance retention after repeated cycles would significantly strengthen the claims of real-world applicability.

Reviewer #3

(Remarks to the Author)
This study reports a wood-based ion-selective power generator designed to convert ionic gradients in wastewater into electrical energy. Wood membranes were modified by surface-encapsulating cationic or anionic polyelectrolytes through sunlight-induced polymerization, producing ion-selective channels. The modified membranes enable selective ion transport under salinity gradients and generate electricity without external pressure or applied voltage. A single device produced an open-circuit voltage of 55 mV and a current of 0.6 μA in simulated flue gas desulfurization wastewater. The results demonstrate that surface-modified wood membranes can achieve continuous osmotic energy conversion. Please first address the following comments and revise the technical issue to improve the manuscript's quality.

1. Both the voltage and current output of this device are very low, and the whole device takes up a lot of space. Therefore, the volumetric power density is not compelling compared with other energy generators. The author should provide a real scenario of this device and address more about the significance of this work.
2. The authors talk about the circular economy. However, the authors need to make a careful discussion about the cost of wood modification and consider the corresponding payback period, especially since the power output of this device is very low.
3. In Figures 1d and e, it is difficult to see the structure of the wood. I cannot see the sketched and interconnected structures. What is the difference between these two structures?
4. In Figures 2g and h, why do the current and voltage output of the device demonstrate an increasing trend in 1st and 2nd. It should not be the fluctuation. Besides, the performance of the device based on sunlight polymerization demonstrates a totally different behavior with a very stable power output. The authors do not elaborate on the underlying reason behind it. In my opinion, it should be the internal microstructure difference based on the different polymerization method. Please add more discussion in this part. This is very important.
5. Please elaborate on why the redox reaction potential takes place in this work. What does non-equal potential drop mean?
6. How about the universality of the wood? Can this approach be applied to other types of wood or other systems?
7. Figure 4 is poorly arrangement and please reorganize this figure. Actually, this is a big figure, but it only takes up a very small space on this paper. Please rearrange it. Some typos should be revised.

Version 1:

Reviewer comments:

Reviewer #1

(Remarks to the Author)
The authors have addressed all the issues properly. Therefore, I would like to recommend the acceptance of this revised manuscript.

Reviewer #2

(Remarks to the Author)
The comments are addressed properly.

Reviewer #3

(Remarks to the Author)
I have read the response letter with the revised manuscript carefully. The authors have done a great job in their revision, which dispelled all of my concerns. As such, I would like to give my highest recommendation to this wonderful work. In a short summary, this work presents a wood-based ion-selective power generator that converts salinity gradients in wastewater into continuous electricity using surface-encapsulated polyelectrolyte-modified wood membranes. By exploiting wood's hierarchical porous structure, the authors fabricate anion- and cation-selective membranes through dip-coating and sunlight-induced polymerization, achieving a device composed of about 98% bio-based materials. The study provides a low-cost, scalable, and sustainable route for coupling wastewater utilization with osmotic energy harvesting in circular water-energy systems.

Open Access This Peer Review File is licensed under a Creative Commons Attribution 4.0 International License, which permits use, sharing, adaptation, distribution and reproduction in any medium or format, as long as you give appropriate credit to the original author(s) and the source, provide a link to the Creative Commons license, and indicate if changes were made.
In cases where reviewers are anonymous, credit should be given to 'Anonymous Referee' and the source.
The images or other third party material in this Peer Review File are included in the article's Creative Commons license, unless indicated otherwise in a credit line to the material. If material is not included in the article's Creative Commons license and your intended use is not permitted by statutory regulation or exceeds the permitted use, you will need to obtain permission directly from the copyright holder.
To view a copy of this license, visit https://creativecommons.org/licenses/by/4.0/

Reviewer #1

This study proposes an innovative surface encapsulation strategy to fabricate ion-selective wood membranes (S-AWM/S-CWM) with high bio-based content (~98 wt%) and successfully demonstrates their capability to generate electricity from wastewater salinity gradients. This work's core innovation, leveraging wood's natural structure for selective surface modification to avoid pore filling and employing sunlight-induced polymerization, stands out notably. It offers a novel approach for developing low-cost, sustainable osmotic energy harvesting devices. The paper is supported by solid data and comprehensive characterization throughout. However, there is room for improvement regarding the depth of mechanistic explanation and long-term stability in real wastewater systems. I therefore recommend minor revision before the work can be considered for publication. Below are my specific questions and suggestions.

Response: We sincerely appreciate your positive evaluation of our work and the constructive feedback. Your insights have significantly helped us improve the quality and depth of our manuscript. Below is our point-by-point responses to your questions and detail the corresponding revisions made to the manuscript.

Q1: When the system treats wastewater, do electrons from the wastewater participate in the electrode reactions? If ions act as reactants in the electrode reactions that generate current, it might lead to adverse effects such as metal deposition or corrosion on electrical devices.

Response: Many thanks for the question. In our laboratory setup, the electrical current is generated through the RED mechanism driven by the osmotic pressure gradient, not by the direct redox consumption of the wastewater ions (like Cu^{2+} or heavy metals) acting as the primary electron donors/acceptors. We utilized standard Ag/AgCl electrodes as shown in the Figure R1, which undergo reversible reactions ($Ag + Cl^- \rightleftharpoons AgCl + e^-$) to convert the ionic flux traversing the membrane into an electronic current in the external circuit. Therefore, the metal ions from the wastewater do not directly deposit onto the electrodes during short-term operation.

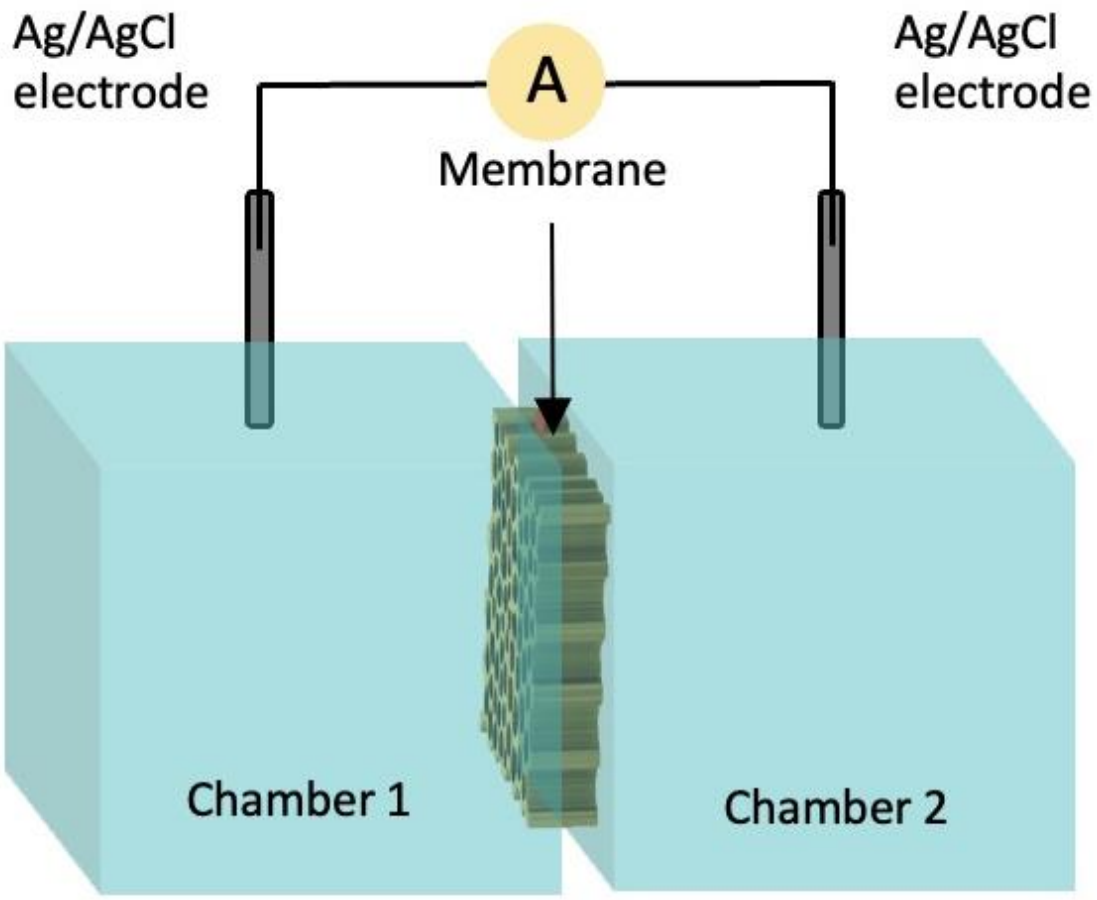

Figure R1. Schematic illustration of the laboratory electrochemical measurement setup. The system utilizes standard Ag/AgCl electrodes in both chambers to detect the electrical current generated by the RED mechanism across the ion-selective wood membrane.

However, you are entirely correct that in long-term, direct application without specialized electrode chambers, there is a risk of ion depletion or electrode fouling. In scaled-up applications, this is typically mitigated by employing isolated electrode chambers with circulating rinse solutions. We have clarified this mechanism in the revised manuscript to prevent any misunderstanding.

Mauscript:

Standard Ag/AgCl electrodes were utilized to convert the trans-membrane ionic flux into electronic current via reversible faradaic reactions ($Ag + Cl^{-} \rightleftharpoons AgCl + e^{-}$). Target wastewater ions do not directly participate as redox reactants, preventing immediate metal deposition. For future scaled-up applications, employing isolated electrode chambers with circulating rinse solutions will effectively mitigate long-term electrode fouling and corrosion.

Q2: Regarding the point on page 11 noting "improved performance but decreased stability at low pH," please elaborate further in the discussion. It must be clearly stated that this limits the direct long-term application of this membrane in strongly acidic wastewater, and potential solutions (such as pre-treatment or pH buffering) should be proposed.

Response: Many thanks for the suggestion. The decreased structural stability at low pH is primarily attributed to the acid-catalyzed hydrolysis of the hemicellulose and

amorphous cellulose within the wood scaffold, which degrades its mechanical integrity over prolonged periods.

To rigorously assess this limitation, we have conducted extended long-term stability tests continuously operating the device at pH=1 for over 4000 minutes. As shown in the newly provided long-term test data (now included as Figure S3 in the Supporting Information), while the open-circuit voltage remains relatively stable for the first ~600 minutes, a notable decline in current output occurs after approximately 1000 minutes, confirming the long-term degradation limits in strongly acidic environments. We have stated this limitation in the manuscript and added the reviewer's suggested solutions, emphasizing that practical deployment in highly acidic effluents will necessitate pre-treatment steps such as pH buffering.

Manuscript:

Add the following paragraph to the manuscript:

To rigorously evaluate the operational robustness, extended continuous stability tests were conducted at pH 1 (Figure S3). The results demonstrate that while the S-AWM maintained a stable open-circuit voltage and a steady short-circuit current plateau for the first ~1000 minutes, prolonged exposure eventually led to a decline in current. This degradation is primarily attributed to the acid-catalyzed hydrolysis of hemicellulose and amorphous cellulose compromising the wood's structural integrity. Despite this limitation, the inherent hierarchical structure of wood, reinforced by its highly cross-linked lignin matrix, provides significant dimensional stability against macroscopic swelling. Furthermore, the covalent crosslinking of the polyelectrolyte layer via sunlight-induced polymerization prevents rapid polymer leaching under varying ionic strengths. Nevertheless, we acknowledge that for continuous operation over weeks or months in real-world organic-rich wastewater, wood-based substrates may be susceptible to bio-fouling and slow microbial degradation. Therefore, for industrial relevance and real-world implementation, the execution of standard pre-treatment protocols, such as pH neutralization or buffering, alongside coarse filtration to remove organic fouling species, will be essential to preserve structural integrity and prolong the device's operational lifespan.

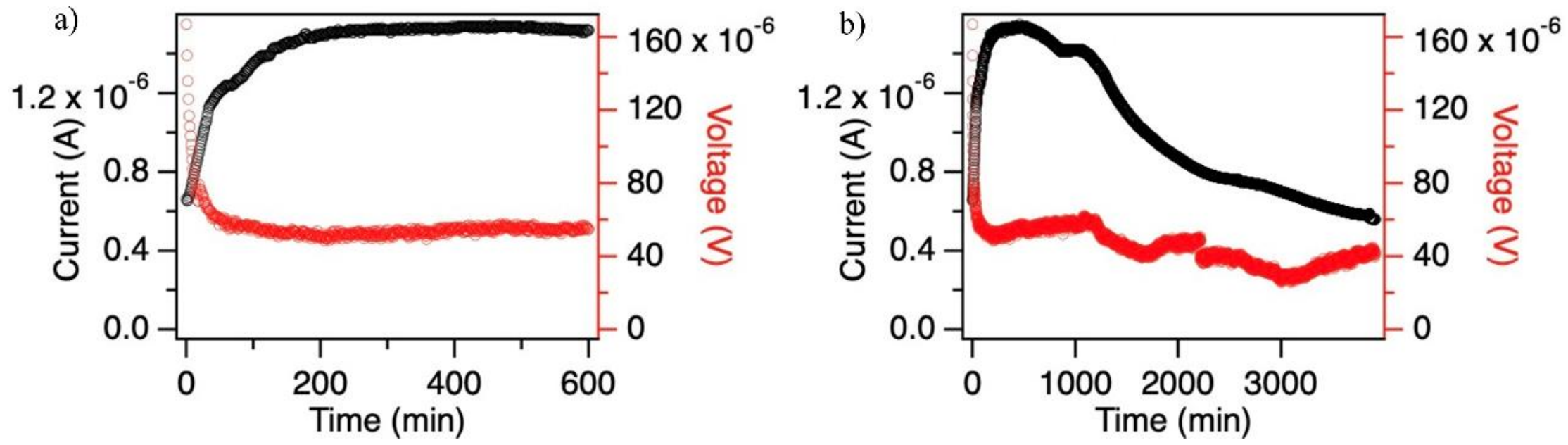

Figure S3. Long-term operational stability of the S-AWM osmotic power generator under extremely acidic conditions (pH = 1). The measurements were conducted under a concentration gradient of 1 mM $Cu(NO_3)_2$ solution versus deionized (DI) water. (a) I_{sc} and V_{oc} monitored over the initial 600 minutes. (b) Extended continuous stability test over 4000 minutes.

Q3: The term "modulated wastewater" is used multiple times in the text. It is recommended to clearly specify the exact source (e.g., citing literature) and rationale for selecting its simulated formulation on page 15 or in the methods section, to strengthen the representativeness and reproducibility of the experimental design.

Response: Thank you for highlighting this oversight. We agree that the rationale and formulation details need to be explicitly detailed in the main text and methods to ensure full reproducibility. We formulated the modulated wastewater based on the reference of Panagopoulos (2022), specifically using the median concentrations of major ions (Na^+, K^+, Ma^{2+}, Ca^{2+}, Cl^-, SO_4^{2-}) reported across various industrial effluents. We have expanded the "Chemicals and materials" section to clarify this methodology.

Manuscript:

In the "Chemicals and materials" section, we added the following text: To represent typical real-world conditions, the simulated ("modulated") wastewater for various industries was formulated based on the median concentrations of major ions reported in the literature[2]. The specific mass of salts added to 150 mL of water to achieve these simulated concentrations is detailed in Figure S20.

2 Panagopoulos, A. Study and evaluation of the characteristics of saline wastewater (brine) produced by desalination and industrial plants. Environmental Science and Pollution Research 29, 23736-23749 (2022).

Q4: Please review and standardize the format of units throughout the entire text (e.g.,

ensure the use of "μm" instead of "um").

Response: We apologize for the error. We have conducted a thorough review of the entire manuscript and Supporting Information to standardize all unit formatting. All instances of "um" have been corrected to "μm".

Manuscript:

1. The connection between tracheids is dependent on abundant bordered pit, with a pit aperture diameter around 1.8~ 6.5 μm, which efficiently enhance ion selectivity through their sophisticated pore morphology.

2. This is primarily because pristine wood lacks effective ion-selective mechanisms: its tracheid pore size (≈10–100 μm) is far larger than ion diameters (nm scale), leading to negligible steric effect; meanwhile, the weak charge density of native wood polymers (cellulose/hemicellulose) results in a negligible Donnan effect.

Q5: Please check the grammar and spelling throughout the full text. For example, on page 3/line 91, "wood' inherent" should be "wood's inherent."

Response: We are sorry for these errors. We have corrected "wood' inherent" to "wood's inherent" as suggested.

Manuscript:

1. Due to wood's inherent structural characteristics, 1 mm-thick wood membranes are common preferred for efficient infiltration and high flow rates.

2. To address this, we herein leverage wood's inherent hierarchical structure and relatively high specific surface area to focus on cell wall surface modification, avoid lumen filling.

Reviewer #2

The manuscript presents an interesting concept by combining bio-based materials with osmotic energy harvesting from wastewater, and the idea of using wood as a hierarchical scaffold for ion-selective transport is appealing from both sustainability and materials engineering perspectives. The work is clearly motivated by circular economy considerations and demonstrates a creative attempt to integrate wastewater utilization with energy recovery. However, in its current form, the study requires major revision before it can be considered for publication in Nature Communications, primarily because several key aspects related to scientific depth, practical relevance, and validation remain insufficiently addressed.

Response: We sincerely thank the reviewer for the rigorous and constructive evaluation of our manuscript. In this revision, we have included quantitative benchmarking, extended long-term stability data (exceeding 4000 minutes), and deepened our mechanistic discussion on structure-transport relationships. We believe these comprehensive revisions have thoroughly addressed your concerns and aligned the manuscript with the rigorous standards of *Nature Communications*.

Q1: The central claim of scalability and practical applicability needs to be supported more convincingly. While the manuscript shows that multiple cells connected in series can increase voltage output and power small electronic devices, the demonstrated performance remains at a laboratory scale, and the pathway toward realistic scale-up is not fully discussed. For example, it is not clear how performance would evolve when moving from centimeter-scale wood blocks to module-scale assemblies where internal resistance, concentration polarization, and hydraulic management become dominant limitations. A quantitative discussion of power density, projected module footprint, and comparison with existing osmotic energy technologies would significantly strengthen the impact. At present, the scale-up argument is largely conceptual rather than engineering-driven.

Response: We completely agree with the reviewer that transitioning from a laboratory scale to a module-scale assembly entails complex engineering challenges. In the revised manuscript, we have added a dedicated quantitative discussion to address scalability.

Specifically, we calculated the maximum power density (P_{max}) of our current single S-AWM cell (active area ~1 cm^2, I_{sc} = 0.6 μA, V_{oc} = 55 mV) to be approximately 0.08 mW/m^2. While this absolute areal power density is lower than state-of-the-art ultrathin 2D nanofluidic membranes, our bulk wood-based system offers an unprecedented advantage in cost-effectiveness and module stacking volume. We also explicitly acknowledge that during scale-

up, internal resistance an ICP will become dominant limitations. We have now proposed in the text that future engineering designs must focus on optimizing fluid channel hydrodynamics to minimize boundary layer thickness and mitigate ICP.

Manuscript:

To realistically evaluate the pathway toward large-scale application, a quantitative assessment of the current power output is necessary. For a single S-AWM cell (active area of 1 cm^2), the calculated maximum power density ($P_{max} = I_{sc} \times V_{oc} / 4$) is approximately 0.08 mW/m^2. Transitioning from centimeter-scale blocks to module-scale assemblies will inevitably introduce engineering challenges, primarily dominated by increased internal resistance and ICP. To mitigate these limitations in future scaled-up plants, hydraulic management must be optimized. Thus, while the current scale-up demonstration is foundational, it paves the way for specific hydrodynamic engineering to realize industrial osmotic power modules.

Q2: The stability assessment is another major limitation. The electrical performance is mainly evaluated over relatively short durations, typically on the order of tens of minutes to one hour, as shown in the stability plots where current and voltage are monitored over about 60 minutes. This is not sufficient to support claims of continuous electricity generation or industrial relevance. Long-term stability under realistic operating conditions, including cyclic salinity gradients, continuous operation over days or weeks, and exposure to real wastewater containing organic matter or fouling species, is necessary. In particular, wood-based substrates may undergo swelling, microbial degradation, or structural fatigue in aqueous environments, and these aspects are not addressed. It would also be important to understand whether the polyelectrolyte layer leaches or degrades during operation, especially under varying pH or ionic strength conditions.

Response: We sincerely thank the reviewer for identifying this critical gap. While conducting multi-week or multi-month trials in real, unfiltered wastewater containing complex organic foulants is extremely challenging within the revision timeframe, we have comprehensively addressed your concerns through a combination of extended accelerated stress testing, theoretical material analysis, and transparent discussion of operational boundaries.

(1) Long-term stability and leaching (Accelerated Stress Test): To substantiate our claims of chemical stability and evaluate polyelectrolyte degradation, we conducted extended continuous operational tests for over 4000 minutes (~66 hours) under extreme corrosive stress (pH = 1). As presented in the newly added Figure S3, the open-circuit voltage remains highly stable, and the current output maintains a steady plateau for the first ~1000 minutes before

exhibiting degradation. This severe condition serves as an accelerated test, proving that the covalent crosslinking of the polyelectrolyte layer via sunlight-induced polymerization effectively anchors the functional groups and prevents rapid leaching under varying ionic strengths.

(2) Swelling and structural fatigue: Unlike pure synthetic hydrogels that suffer from severe macroscopic swelling and structural fatigue in aqueous environments, the hierarchical channels of our wood-based substrate are naturally reinforced by a highly cross-linked lignin matrix. This intrinsic structural rigidity provides far superior dimensional stability, effectively resisting severe swelling during continuous exposure to aqueous gradients.

(3) Exposure to real wastewater and biological fouling: We explicitly agree with your assessment regarding real wastewater environments. In organic-rich effluents, bio-fouling and slow microbial degradation of the cellulose components are inevitable over extended months. Rather than claiming absolute immunity, we have integrated these practical realities into the revised manuscript. We now clearly stress that for industrial relevance, standard pre-treatments, such as coarse filtration to remove organic foulants and pH buffering to prevent acid-catalyzed hydrolysis, are necessary prerequisites prior to feeding the wastewater into the actual RED modules.

We believe this transparent approach, supported by our extended 66-hour continuous baseline stability data, provides a much more rigorous and realistic evaluation of the device's practical applicability.

Manuscript:

We added the following paragraph to the manuscript:

Despite this impressive initial performance at extreme acidity, the long-term chemical durability of the device must be carefully contextualized within varying industrial environments. To rigorously evaluate the operational robustness, extended continuous stability tests were conducted at pH 1 (Figure S3). The results demonstrate that while the S-AWM maintained a stable open-circuit voltage and a steady short-circuit current plateau for the first ~1000 minutes, prolonged exposure eventually led to a decline in current. This degradation is primarily attributed to the acid-catalyzed hydrolysis of hemicellulose and amorphous cellulose compromising the wood’s structural integrity.

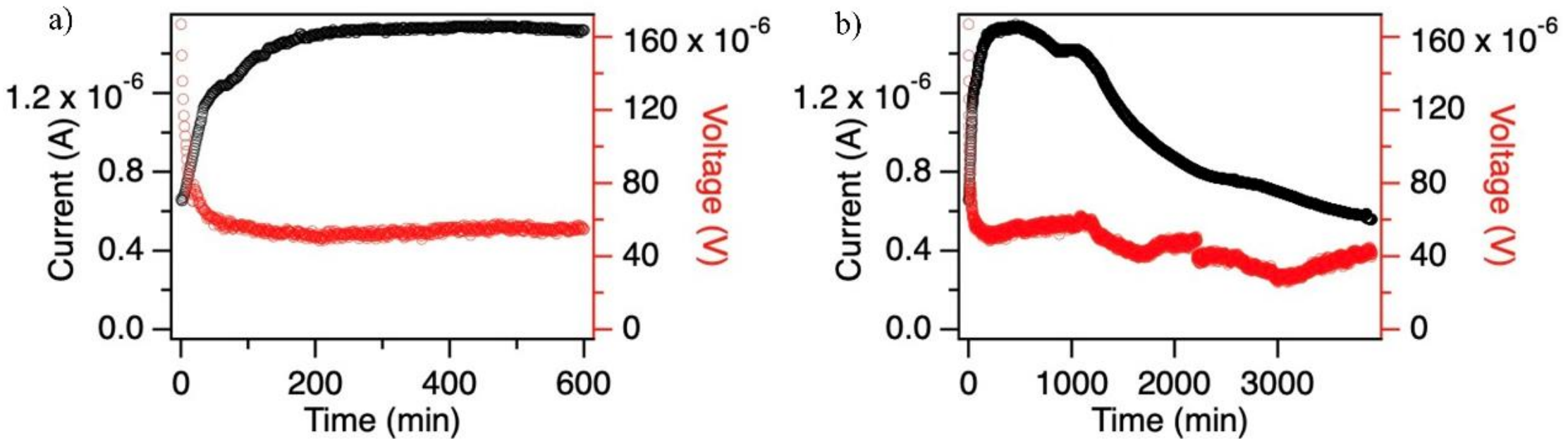

Figure S3. Long-term operational stability of the S-AWM osmotic power generator under extremely acidic conditions (pH = 1). The measurements were conducted under a concentration gradient of 1 mM $Cu(NO_3)_2$ solution versus deionized (DI) water. (a) I_{sc} and V_{oc} monitored over the initial 600 minutes. (b) Extended continuous stability test over 4000 minutes.

Q3: The manuscript would also benefit from clearer benchmarking against established ion-selective membranes and osmotic energy systems. The reported voltages and currents are compared primarily with pristine wood rather than with state-of-the-art osmotic power membranes such as nanofluidic membranes, ion-exchange membranes, or other bio-based systems. Without such benchmarking, it is difficult to assess whether the observed performance represents a meaningful advance or mainly demonstrates proof-of-concept functionality. Including normalized metrics such as power density per membrane area and comparing them with literature values would help clarify the significance of the results.

Response: We sincerely appreciate the reviewer's constructive suggestion, which adds essential context and depth to our findings. In response, we have introduced a comprehensive benchmarking discussion in the revised manuscript and added a detailed comparison table (**Table S1**) in the Supporting Information. This new table systematically benchmarks our S-AWM/CWM systems against state-of-the-art 3D bulk biomaterials and 2D reconstituted osmotic membranes.

As requested, we have included normalized metrics (normalized power density, W m^{-2}) and compared them with literature values. More importantly, to comprehensively assess the "meaningful advance" of our work for practical applications, we also benchmarked critical industrial metrics: pre-treatment methods, fabrication scalability, external energy input, and bio-based content.

Our benchmarking reveals a crucial trade-off in the current osmotic energy field. While ultrathin 2D reconstituted membranes and heavily processed 3D materials achieve remarkably high peak power densities per unit area due to extreme nanoscale confinement, they are fundamentally bottlenecked by energy-intensive, unscalable fabrication processes (e.g., vacuum filtration, electrospinning) and harsh, destructive chemical pre-treatments (e.g., acid

exfoliation, severe delignification).

In contrast, our system represents a paradigm shift from lab-scale peak performance to massive industrial volume-to-cost efficiency. The meaningful advance here is achieving a highly functional RED membrane that thoroughly resolves the scalability and sustainability bottlenecks. Our system uniquely preserves the pristine macroscopic structure of wood (zero destructive pre-treatment) and utilizes a completely solvent-free, zero-external-energy (sunlight-driven) dip-coating process. By utilizing >98 wt% raw natural biomass, our device trades lab-scale peak areal power density for unparalleled fabrication scalability, structural integrity, and bulk material affordability. We have significantly reframed the discussion in the revised manuscript to clearly highlight this paradigm shift and the practical significance of our results.

Manuscript:

In the end of the disscussion, we added the following text:

To contextualize the significance of this work, we benchmarked S-AWM against state-of-the-art osmotic membranes across structural integrity, fabrication scalability, and performance (Table S1). While ultrathin 2D films and heavily processed 3D biomaterials can exhibit higher absolute power densities, they heavily rely on destructive chemical pre-treatments, unscalable vacuum filtration, and energy-intensive fabrication. Conversely, S-AWM strategically preserves the pristine wood matrix through a zero-energy, sunlight-driven process. Although sacrificing some peak areal power density, S-AWM possesses overwhelming advantages in environmental sustainability, energy efficiency, and industrial scalability.

Table S1 Structural, fabrication, and performance comparison of S-AWM/S-CWM against state-of-the-art synthetic and bio-based osmotic membranes

**Materials**	**Main Content**	**Membrane Structure & Scalability**	**Pre-treatment methods**	**Fabrication Process**	**External Energy Input**	**Normalized Power Density**	**Reference**
**S-AWM**	**98%Pristine wood + 2%Polymer**	**3D Bulk**	**None**	**Dip-coating + Sunlight**	**None**	**0.08 mW/m^2**	**This work**
Ionized Wood Membrane	Delignified wood + Epoxy resin	3D Bulk	Delignification	Chemical oxidation of wood	Yes	5.14 mW/m^2	[1]
Wood /sodium alginate	Delignified wood + Sodium alginate	3D Bulk	Delignification	SA infiltration	Yes	14.43 W/m^2	[2]
Wood WEG	Delignified wood + Wood-derived carbon	3D Bulk	Delignification	Carbon coating	Yes	0.67 W/m^2	[3]
Bark	Delignified wood bark	3D Bulk	Delignification	Densification	Yes	1.45 W/m^2	[4]
ISWM PWM	Delignified wood	3D Bulk	Delignification	Hole engineering	Yes	0.658 W/m^2	[5]
Dual-network fiber-hydrogel	Cellulose + Synthetic hydrogel	3D Bulk	Synthetic copolymerization	Cross-linking	Yes	6.75 W/m^2	[6]
Reed membrane	Natural reed	3D Bulk	Physical cutting	Chemical activation	Yes	2.6 W/m^2	[7]

KL/TCNF-PEDOT:PSS	79% TCNF + 15% Kraft Lignin	2D Film	Lignin/Cellulose extraction & mixing	Vacuum filtration	Yes	1.37 W/m^2	[8]
KCNF/MXene	40% Kelp CNF	2D Film	Acid exfoliation & mixing	Vacuum filtration	Yes	10.56 W/m^2	[9]
2D-HM	Chitin nanosheets	2D Film	Chemical exfoliation	Filtration	Yes	2.59 W/m^2	[10]
CSs Membrane	Chitin sub-nanosheets	2D Film	Chemical exfoliation	Vacuum filtration	Yes	8.31 W/m^2	[11]
CNFs/CNTs	~ 50% Cellulose Nanofibers	2D Film	Chemical extraction & mixing	Vacuum filtration	Yes	4.67 W/m^2	[12]
KANF	Synthetic Kevlar aramid	2D Film	Kevlar synthetic processing	Vacuum filtration	Yes	4.8 W/m^2	[13]
C-MXene/C-HNF	20% Holocellulose HNF	2D Film	Chemical exfoliation & mixing	Vacuum filtration	Yes	1.09 W/m^2	[14]
Natural cellulose n-membrane	Natural cellulose	2D Film	Chemical extraction	Vacuum filtration	Yes	2.6 W/m^2	[15]
A-CNF	Modified cellulose	2D Film	Chemical modification	Filtration	Yes	0.81 W/m^2	[16]
C-CNF	Modified cellulose	2D Film	Chemical modification	Filtration	Yes	1.4 W/m^2	[16]
PBONM	Synthetic PBO polymer	2D Film	Toxic solvent dissolution	Electrospinning & Filtration	Yes	7.7 W/m^2	[17]
PNIPAM-g-sCC	~ 40% Cellulose core	2D Film	Graft copolymerization	Filtration	Yes	10.1 W/m^2	[18]
RCNF	Regenerated cellulose	2D Film	Cellulose dissolution	Wet-spinning & Regeneration	Yes	2.57 W/m^2	[19]

References

1 Wu, Q. Y. *et al.* Salinity-gradient power generation with ionized wood membranes. *Adv. Energy Mater.* **10**, 1902590 (2020).

2 Zhou, T. *et al.* Leveraging Natural Wood Structures for Sustainable and High-Performance Osmotic Energy Harvesting. *Small*, e13371 (2026).

3 Zhang, K. *et al.* All-wood-based ionic power generator with dual functions for alkaline wastewater reuse and energy harvesting. *Acs Nano* **18**, 10259-10269 (2024).

4 Song, G. *et al.* Self-adhesive ionic cable derived from natural bark as osmotic energy generator. *Nano Energy* **131**, 110326 (2024).

5 Yang, T. *et al.* Advanced ion-selective wood membranes: Leveraging aligned cellulose nanofibers for enhanced osmotic energy conversion. *Chem. Eng. J.* **496**, 153891 (2024).

6 Cao, L. & Wu, H. Dual-network fiber-hydrogel membrane for osmotic energy harvesting. *Frontiers in Chemistry* **12**, 1401854 (2024).

7 Zheng, X. *et al.* Renewable, All-Natural Flute Membrane with Excellent Mechanical Properties for Osmotic Energy Harvesting. *ACS Appl. Mater. Interfaces* **16**, 70618-70625 (2024).

8 Xu, A., Qi, S., Ahmad, M. & Wang, S. Industrial Kraft Lignin-Functionalized Cellulose Nanofluidic Membrane for Enhanced Osmotic Energy Conversion. *ACS Applied Nano Materials* **8**, 2974-2985 (2025).

9 Zhai, R. *et al.* Kelp Nanofiber-Based Composite Membranes for Highly Efficient Osmotic Energy Conversion. *Adv. Funct. Mater.* **34**, 2313914 (2024).

10 Xiang, Z. *et al.* Sustainable Chitin-Derived 2D Nanosheets with Hierarchical Ion Transport for Osmotic Energy Harvesting. *Adv. Energy Mater.* **14**, 2402304 (2024).

11 Shu, Y. *et al.* 2D Chitin Sub-Nanosheets With Extreme Ion Transport for Nanofluidic Sensing. *Adv. Mater.* **38**, e10095 (2026).

12 Li, X., Xiao, T., Lu, B., He, J. & Zhai, J. Double-network ion channels for high-performance osmotic power generation. *Adv. Mater. Interfaces* **9**, 2101960 (2022).

13 Ding, L. *et al.* Ultrathin and ultrastrong kevlar aramid nanofiber membranes for highly stable osmotic energy conversion. *Adv. Sci.* **9**, 2202869 (2022).

14 Rao, J. *et al.* Nacre-Inspired Mechanically Robust Films for Osmotic Energy Conversion. *Adv. Funct. Mater.* **34**, 2309869 (2024).

15 Chen, C. *et al.* Nanofluidic osmotic power generation from CO2 with cellulose membranes. *Green Carbon* **1**, 58-64 (2023).

16 Wang, S., Sun, Z., Ahmad, M., Fu, W. & Gao, Z. Engineered cellulose nanofibers membranes with oppositely charge characteristics for high-performance salinity gradient power generation by reverse electrodialysis. *Int. J. Biol. Macromol.* **253**, 126608 (2023).

17 Duan, R. *et al.* High-strength, thin PBO nanofiber membrane with long-term stability for osmotic energy conversion. *Adv. Funct. Mater.* **34**, 2311258 (2024).
18 Lin, X. *et al.* Temperature-gated nanocellulose membrane for enhanced and controllable osmotic energy harvesting. *Nano Energy* **107**, 108156 (2023).
19 Zhou, B. *et al.* Aligned regenerated cellulose-based nanofluidic fibers with ultrahigh ionic conductivity and underwater stability for osmotic energy harvesting. *Chem. Eng. J.* **457**, 141167 (2023).

Q4: The mechanistic discussion of ion selectivity could also be strengthened. The work attributes improved performance to surface-encapsulated polyelectrolytes and hierarchical wood channels, but quantitative characterization of charge density, effective pore size, or transport selectivity is limited. For example, transference number measurements are shown, but the link between polymer distribution, crosslinking degree, and ion transport efficiency remains largely qualitative. A deeper structure–transport relationship analysis would make the study more compelling for a high-impact audience.

Response: We agree that a deeper structure-transport analysis is necessary. In the revised manuscript, we have expanded our mechanistic discussion linking the crosslinking degree to transport selectivity.

We have now elaborated on the mechanism where crosslinking degrees of 1%, 5%, and 50% were evaluated (Figure S19). A low crosslinking degree allows the PDMAEMA/PAA chains to remain extended, providing effective overlapping Debye screening lengths within the micro-channels while retaining high free volume for water permeability. Conversely, a highly crosslinked network (50%) drastically increases the localized charge density but rigidly restricts the effective free volume size of the nano-channels. This creates excessive steric hindrance and increases internal electrical resistance, causing the observed dramatic drop in both I_{sc} and V_{oc}. Thus, optimal ion transport efficiency relies on providing sufficient charge density for Donnan exclusion while maintaining adequate free volume for high-flux mass transport.

Manuscript:

In the section of "Feature parameters for ion selectivity and energy generation", the

following text was added:

To investigate the structure-transport relationship and understand how nanochannel size influences ion selectivity, we evaluated the energy conversion behavior of S-AWM under a 100 mM/1 mM salinity gradient across three polymer crosslinking degrees (1%, 5%, and 50%) (Figure S19). The results indicate that an intermediate crosslinking degree (5%) yields the optimal electrical output, whereas a highly crosslinked network (50%) exhibits a dramatic decline in both I_{sc} and V_{oc}. Mechanistically, a low-to-intermediate crosslinking degree allows the polyelectrolyte chains to remain extended, providing effective overlapping Debye screening lengths for ion selectivity while retaining high free volume for water permeability. Conversely, while highly crosslinked networks possess a higher localized charge density per unit volume, they rigidly restrict the effective free volume size of the channels. This creates excessive steric hindrance for ion mobility and significantly increases the internal membrane resistance, resulting in lower permeability to water[41]. Therefore, achieving optimal ion transport efficiency (t_n ~ 0.6-0.7, Figure S11) dictates a careful balance: providing sufficient charge density for Donnan exclusion while maintaining adequate free volume for high-flux ionic mass transport. As actual energy generation is also significantly affected by operating conditions, such as the load resistor and solution flow rates, which determine the boundary layer thickness[42], optimizing this internal structural balance is critical.

Q5: Another point that requires clarification is the durability of the device under varying chemical environments. The manuscript itself notes reduced stability at low pH due to corrosion effects, which raises concerns about robustness in industrial wastewater streams where pH and ionic composition fluctuate. Demonstrating chemical stability, mechanical integrity, and performance retention after repeated cycles would significantly strengthen the claims of real-world applicability.

Response: The reviewer raises a highly valid point regarding fluctuating industrial environments. While our extended 4000-minute test at pH 1 (Figure S3) proves that the membrane resists immediate failure and maintains core electrical generation mechanics under severe corrosive stress, we recognize that true industrial robustness requires

transparently addressing material limits. Natural wood is intrinsically sensitive to extreme alkaline or highly acidic conditions over extended months due to the gradual degradation of hemicellulose and lignin networks. We have revised our discussion to clarify that our wood-based membrane is ideally suited for mildly acidic to neutral wastewater streams (e.g., pH 4–8), which covers a large subset of municipal and specific industrial effluents (such as dairy and aquaculture, as simulated in our tests). For highly aggressive or violently fluctuating chemical streams, we now explicitly state that essential pre-treatment (buffering) is required to ensure long-term mechanical integrity and prevent structural corrosion.

Manuscript:

In the section of “Feature parameters for ion selectivity and energy generation”, the following text was added:

Because natural wood is intrinsically sensitive to such prolonged exposure to extreme acidic or alkaline conditions, the current S-AWM is ideally suited for mildly acidic to neutral wastewater streams (e.g., pH 4–8). This operational window effectively covers a vast subset of effluents, including municipal, dairy, and aquaculture wastewater (as simulated in Figure 4). Within these applicable environments, the inherent hierarchical structure of wood, reinforced by its highly cross-linked lignin matrix, provides significant dimensional stability against macroscopic swelling. Furthermore, the covalent crosslinking of the polyelectrolyte layer via sunlight-induced polymerization prevents rapid polymer leaching under varying ionic strengths. Nevertheless, we acknowledge that for continuous operation over weeks or months in real-world organic-rich wastewater or chemically fluctuating wastewater, wood-based substrates may be susceptible to bio-fouling and slow microbial degradation, and chemical corrosion. Therefore, for industrial relevance and real-world implementation, the execution of standard pre-treatment protocols, such as pH buffering to neutralize highly aggressive streams, alongside coarse filtration to remove organic fouling species, will be essential to preserve structural integrity of the wood scaffold and prolong the device’s operational lifespan.

Reviewer #3

This study reports a wood-based ion-selective power generator designed to convert ionic gradients in wastewater into electrical energy. Wood membranes were modified by surface-encapsulating cationic or anionic polyelectrolytes through sunlight-induced polymerization, producing ion-selective channels. The modified membranes enable selective ion transport under salinity gradients and generate electricity without external pressure or applied voltage. A single device produced an open-circuit voltage of 55 mV and a current of 0.6 μA in simulated flue gas desulfurization wastewater. The results demonstrate that surface-modified wood membranes can achieve continuous osmotic energy conversion. Please first address the following comments and revise the technical issue to improve the manuscript's quality.

Response: We sincerely thank the reviewer for summarizing the core contributions of our work so accurately and for providing highly constructive feedback. We have carefully addressed each of your comments, adding necessary discussions, clarifications, and figure modifications to enhance the scientific depth and clarity of the paper. Detailed point-by-point responses are provided below.

Q1: Both the voltage and current output of this device are very low, and the whole device takes up a lot of space. Therefore, the volumetric power density is not compelling compared with other energy generators. The author should provide a real scenario of this device and address more about the significance of this work.

Response: We agree with the reviewer that the absolute volumetric power density of our bulk wood-based device is lower than that of state-of-the-art ultrathin 2D nanomembrane generators. However, the true significance of this work lies in a paradigm shift from "high power density per unit volume" to "extreme cost-effectiveness and massive scalability". In real-world industrial scenarios, such as massive holding ponds for flue gas desulfurization (FGD) wastewater or municipal wastewater treatment plants, available physical space is abundant, but the capital expenditure (CAPEX) for membrane materials is a prohibitive bottleneck. Instead of using expensive, fragile, and non-biodegradable synthetic nanomembranes (which cost

hundreds of dollars per square meter), our approach utilizes abundant natural wood and low-cost monomers (constituting ~98 wt% bio-based content). The significance of this work is demonstrating that we can achieve stable osmotic energy recovery directly from dirty, complex wastewater streams using a device that is structurally robust, extremely cheap to manufacture, and eco-friendly. We have added a discussion on this real-world scenario and the trade-off between power density and scalability in the revised manuscript to better highlight the work's significance.

Manuscript:

In the "Scalability and viability in water-energy circular economy cycle" section, add the following text:

This makes the device particularly ideal for real-world industrial scenarios with abundant spatial footprints, such as large-scale holding ponds for flue gas desulfurization (FGD) or municipal wastewater, where spatial footprint is less of a limiting factor than the prohibitive capital cost of commercial membranes.

Q2: The authors talk about the circular economy. However, the authors need to make a careful discussion about the cost of wood modification and consider the corresponding payback period, especially since the power output of this device is very low.

Response: We deeply appreciate for your comments.

We fully agree that based solely on the current absolute power output (0.08 mW/m^2), calculating a traditional payback period strictly from electricity generation yields a prolonged timeframe. However, the techno-economic feasibility of our device is grounded in its ultra-low Capital Expenditure (CAPEX). As noted in the literature (Daniilidis et al., *Applied Energy*, 2014, 119, 257-265), for osmotic power plants to be economically viable, the membrane cost must approach ~4 €/m^2 (~\$4.5/m^2), a target impossible for commercial synthetic membranes (e.g., Nafion) which typically cost \$500-\$1000/m^2.

By contrast, our material cost is orders of magnitude lower. Raw spruce wood (3 mm thick) costs approximately \$1~\$3/m^2, and the functionalization relies on inexpensive commodity monomers driven by free sunlight. The total material cost

easily approaches the theoretical threshold of <$10/m^2. In a circular economy context, the "payback" stems not only from grid-scale energy sales but from deploying a biodegradable, ultra-low-cost module that offsets the energy typically consumed by traditional wastewater treatment. We have incorporated a concise techno-economic discussion into the manuscript to address this.

Manuscript:

From a techno-economic perspective, while the current lab-scale power density yields a protracted payback period if evaluated solely on electricity sales, the capital expenditure (CAPEX) paradigm is highly disruptive. As previously reported[12], achieving economically viable osmotic power requires membrane costs to fall below ~$4.5/m^2. High-end commercial synthetic membranes (often costing $500–1000/m^2) are inherently excluded by this threshold. In contrast, the estimated material cost of S-AWM, comprising raw spruce (around $1–3/m^2), bulk monomers, and free sunlight polymerization, can practically approach this target.

Q3: In Figures 1d and e, it is difficult to see the structure of the wood. I cannot see the sketched and interconnected structures. What is the difference between these two structures?

Response: We sincerely appreciate the reviewer's careful observation and valuable feedback.

Figures 1d and 1e are high-magnification transverse cross-sections (10 μm scale) where the broader network is inherently difficult to observe. To clarify this, we added a lower-magnification global SEM image (Figure S14) displaying the large-scale periodic porous network, which is the exact top-down view illustrated in the schematic. We have also provided an enlarged view of the wood structure schematic (Figure R2) from Figure 2b to help you observe these interconnected structures more clearly.

Regarding the difference between Figures 1d and 1e: Their visual similarity at this magnification is actually the intended result of our "surface encapsulation" strategy. Unlike traditional "lumen-filling" methods that visibly block pores (as shown in Figures S16-S18 for our I-AWM samples), our ultra-thin polyelectrolyte layer only coats the

inner tracheid walls. This lack of visible difference perfectly proves that the channels remain unclogged, preserving high-flux fluid transport. The true difference is chemical, which is rigorously confirmed by the FTIR spectra (Figure 1c) and a tenfold increase in ion-selective energy generation.

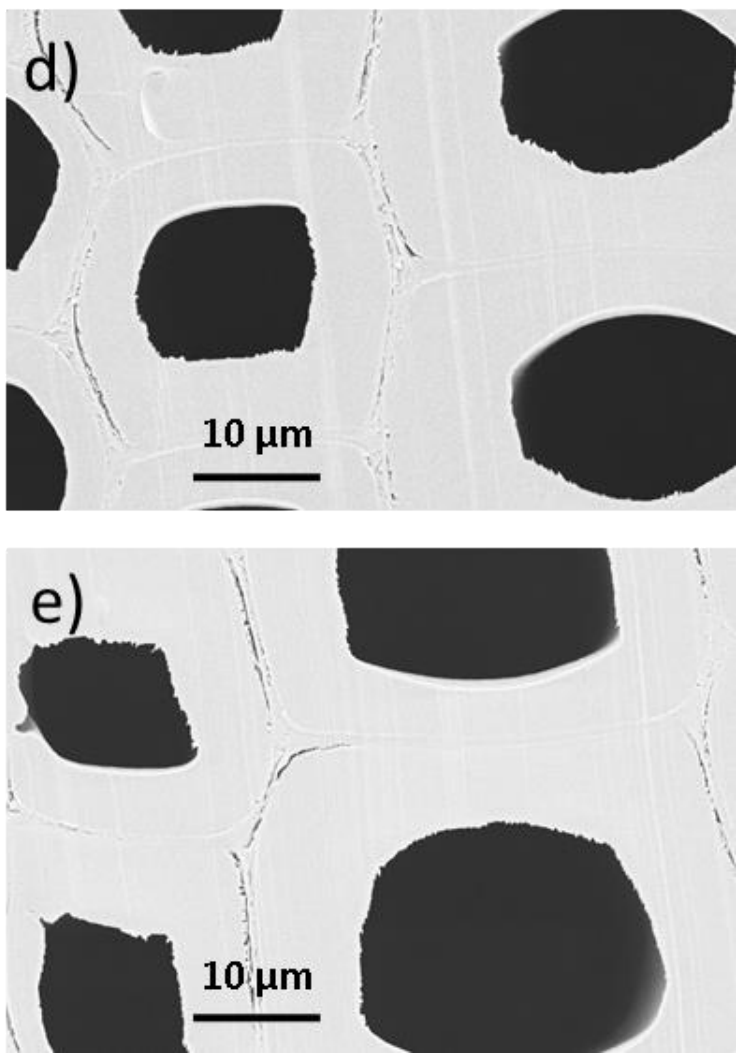

Figure 1 d, e) SEM images of cross sections: d) Morphology of S-AWM and e) Morphology of pristine wood.

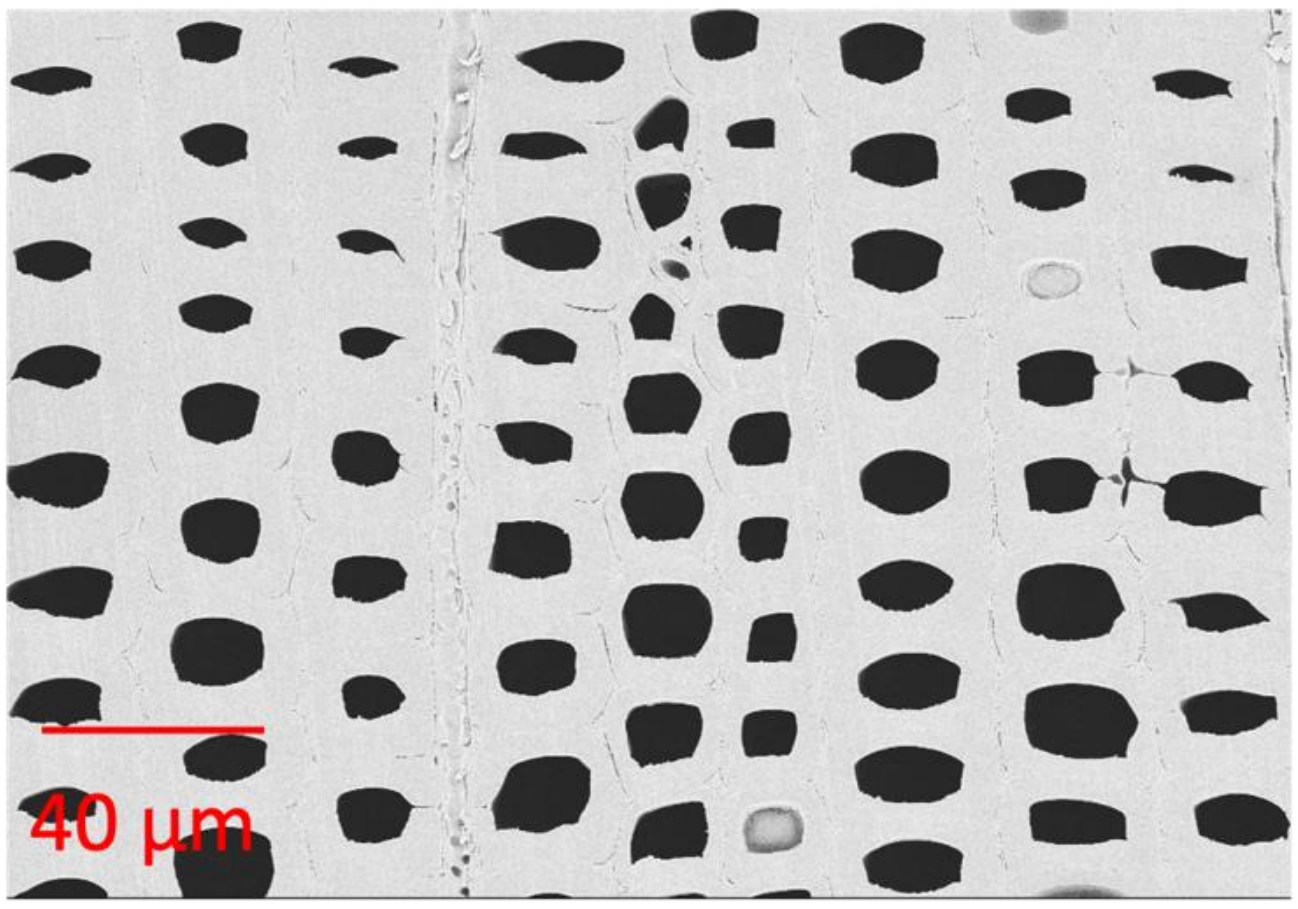

Figure S14. Microtone-cut S-AWM, zoom out.

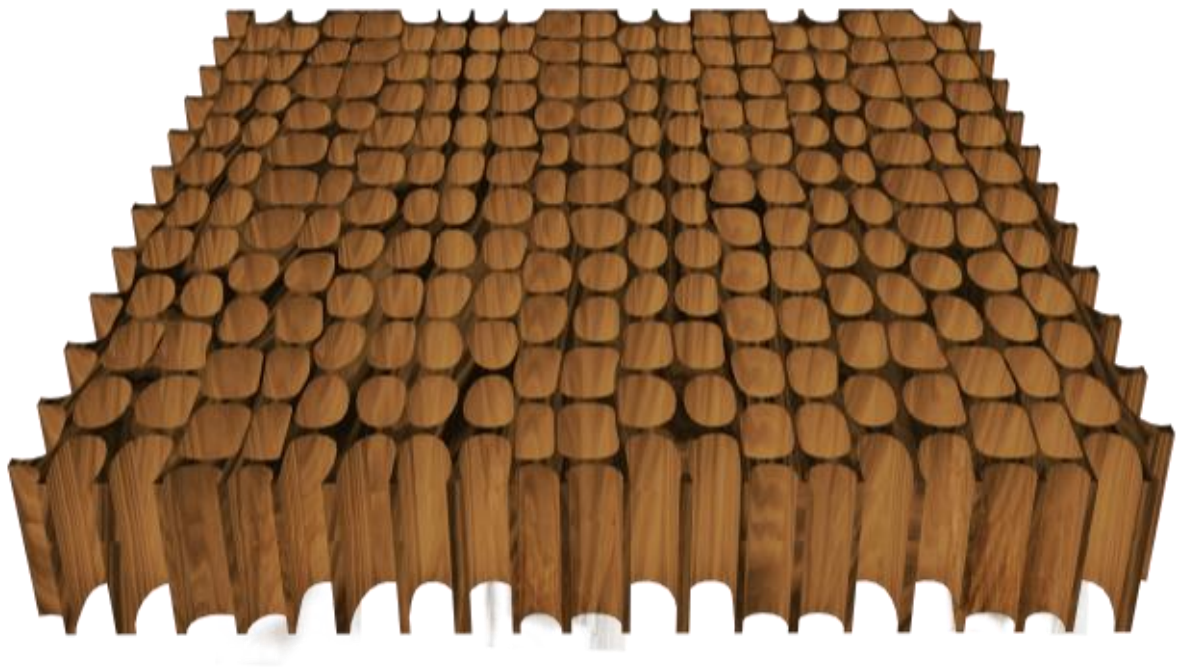

Figure R2. Enlarged 3D schematic of the wood structure illustrating the interconnected tracheid network.

Q4: In Figures 2g and h, why do the current and voltage output of the device demonstrate an increasing trend in 1st and 2nd. It should not be the fluctuation. Besides, the performance of the device based on sunlight polymerization demonstrates a totally different behavior with a very stable power output. The authors do not elaborate on the underlying reason behind it. In my opinion, it should be the internal microstructure difference based on the different polymerization method. Please add more discussion in this part. This is very important.

Response: The reviewer's keen observation and hypothesis are absolutely correct. The distinct electrical behaviors stem from microstructural differences induced by the different polymerization kinetics. The "1st" and "2nd" samples were cured rapidly in a high-intensity UV chamber, which likely created a dense, highly stressed, and slightly inhomogeneous polymer network on the wood surface. When exposed to the electrolyte, this tightly crosslinked network undergoes a slow, continuous hydration and structural relaxation process, leading to the gradual opening of nanochannels and the observed increasing trend in current and voltage. Conversely, the "3rd" sample was polymerized via natural, lower-intensity sunlight over 12 hours. This slow, mild curing kinetics yields a much more uniform, relaxed, and equilibrium-state polymer network. Consequently, it requires minimal hydration equilibrium time, instantly delivering a highly stable power output. We completely agree this is an important point and have incorporated a detailed mechanistic discussion of this phenomenon in the revised text.

Manuscript:

Notably, an observable difference in behavior exists between polymerization methods (Figure 2g and h). The '1st' and '2nd' samples, synthesized via rapid high-intensity UV chamber curing, exhibit a gradual increasing trend in current and voltage. This is attributed to the fast polymerization kinetics creating a denser, internally stressed polymer network that requires a prolonged hydration and structural relaxation period to fully open the ion transport channels. Conversely, the '3rd' sample, polymerized slowly under mild, natural sunlight over 12 hours, demonstrates an immediately stable power output. The slow curing kinetics facilitate the formation of a more uniform, relaxed, and structurally equilibrated polyelectrolyte layer, eliminating the initial hydration lag and highlighting the distinct microstructural superiority of the natural sunlight approach.

Q5: Please elaborate on why the redox reaction potential takes place in this work. What does non-equal potential drop mean?

Response: We apologize for not explaining this electrochemistry concept clearly enough. In our testing setup, we use standard Ag/AgCl reference electrodes to measure the electrical output. The redox reaction potential (E_{redox}) does not refer to the oxidation or reduction of the wastewater itself; rather, it refers to the standard Nernstian potential generated at the interface of the testing electrodes. Because the two fluid chambers contain unequal concentrations of chloride/nitrate salts, the local Nernst potential at the left Ag/AgCl electrode is different from that at the right Ag/AgCl electrode. This difference creates a baseline potential difference in the measurement circuit, which we refer to as the "non-equal potential drop." Therefore, to calculate the pure osmotic potential (V_{os}) exclusively generated by the ion-selective wood membrane, we must mathematically subtract this electrode-induced E_{redox} from the total measured open-circuit voltage (V_{oc}), as shown in Equation 1. We have revised the text to clearly define this mechanism.

Manuscript:

Specifically, rather than originating from the oxidation or reduction of the wastewater itself, this E_{redox} arises because the two fluid chambers possess unequal salt

concentrations. Consequently, the Ag/AgCl testing electrode in each chamber experiences a distinct local Nernstian potential, which creates a baseline "non-equal potential drop" across the measurement circuit. The pure osmotic potential (V_{OS}) contributed by a power source can be obtained by subtracting the contribution from the electrode-solution interface at different concentrations[35]:

Q6: How about the universality of the wood? Can this approach be applied to other types of wood or other systems?

Response: Yes, this approach possesses excellent universality. The fundamental requirement for our strategy is a highly anisotropic, porous cellulosic scaffold with interconnected micro-channels. The Norway spruce used in this study represents a typical softwood with bordered pits connecting the tracheids. This surface-encapsulation approach can be readily translated to other softwoods (e.g., pine, cedar, fir). Furthermore, hardwoods (which contain larger vessels alongside fibers) or even engineered cellulosic sponges and bamboo scaffolds could theoretically be utilized. While different species possess varying pore diameters and permeabilities (which would influence the final water flux and internal resistance), the underlying chemical modification via sunlight-induced polymerization of acrylic monomers is universal to any hydroxyl-rich lignocellulosic surface. We have added a brief statement and a reference to acknowledging this universality.

Manuscript:

Theoretically, this sunlight-induced surface-encapsulation strategy can be universally extended to other wood species and hydroxyl-rich cellulosic scaffolds [32].

Q7: Figure 4 is poorly arrangement and please reorganize this figure. Actually, this is a big figure, but it only takes up a very small space on this paper. Please rearrange it. Some typos should be revised.

Response: We fully accept the reviewer's criticism regarding the poor visual arrangement of Figure 4. We have now completely reorganized and replotted Figure 4. Furthermore, we have carefully proofread the entire figure and manuscript to correct

all grammatical and typographical errors.

Manuscript:

For instance, both textile wastewater and flue gas desulfurization wastewater contain ions like Ca^{2+}, Na^{+}, Mg^{2+}, Cl^{-}, and SO_4^{2-}.

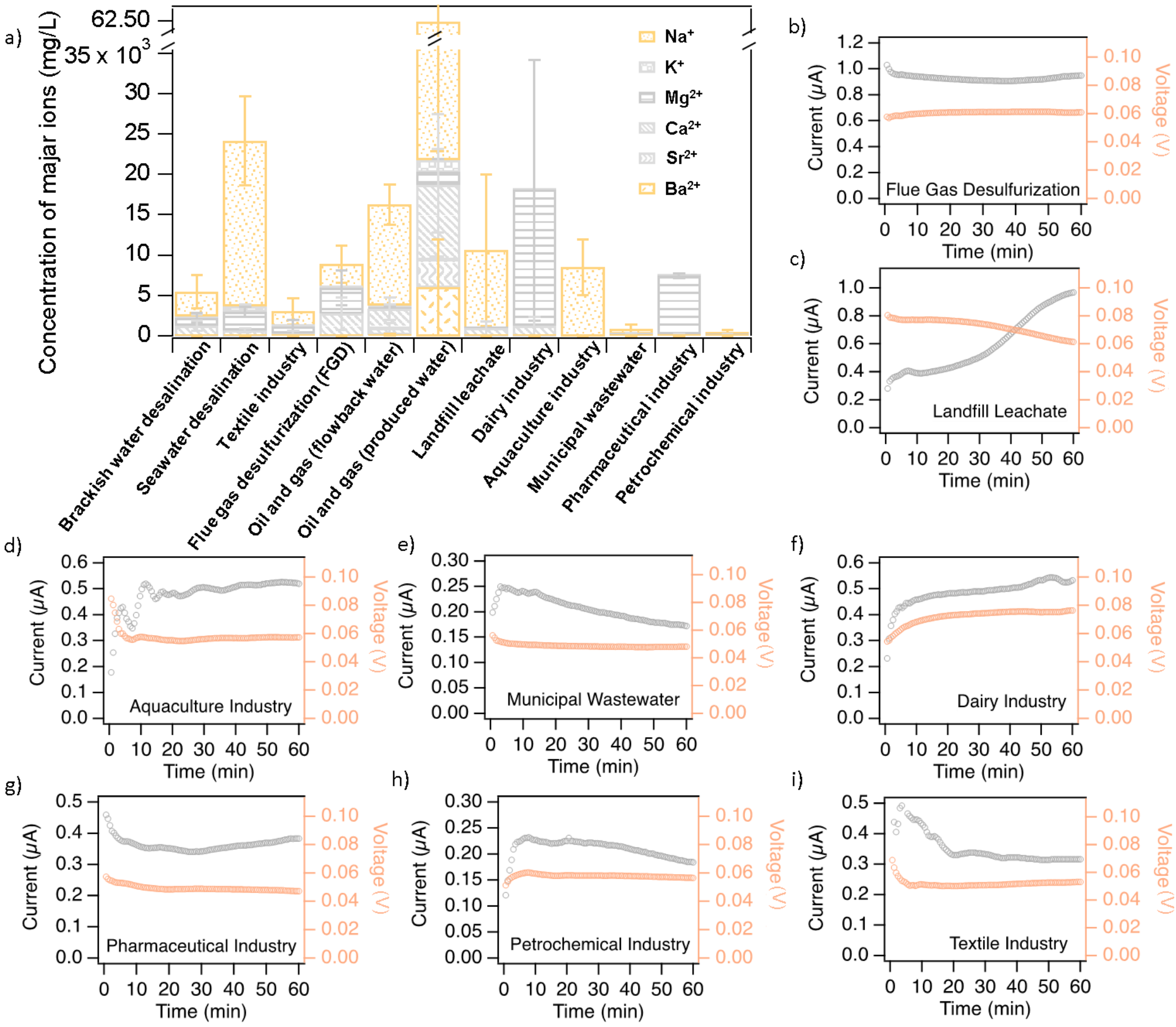

Figure 4. a) The main characteristics of brine effluents from different industries were plotted from data in literature[2]. b) – i) are I_{SC} and V_{OC} generated by S-AWM corresponding to different reaction time from t=0 to t=1h, measured in different industry wastes. The container on the opposite side always contains clean water. The concentration of each characteristic brine solution was simulated as median value in a), see figure S20.

Point-by-Point Response to Reviewers' Comments

We sincerely thank all three reviewers for their time, expertise, and encouraging evaluations of our revised manuscript. We are pleased that our revisions have satisfactorily addressed all previous concerns. Below, we provide a point-by-point response to each reviewer's comments.

Reviewer #1

Comment: *"The authors have addressed all the issues properly. Therefore, I would like to recommend the acceptance of this revised manuscript."*

Response: We sincerely thank Reviewer for the thorough evaluation and for recommending acceptance of our manuscript. We are glad that all previously raised concerns have been adequately addressed.

Reviewer #2

Comment: *"The comments are addressed properly."*

Response: We sincerely thank Reviewer for confirming that all comments have been properly addressed. We appreciate the reviewer's time and constructive feedback throughout the review process.

Reviewer #3

Comment: *"I have read the response letter with the revised manuscript carefully. The authors have done a great job in their revision, which dispelled all of my concerns. As such, I would like to give my highest recommendation to this wonderful work. In a short summary, this work presents a wood-based ion-selective power generator that converts salinity gradients in wastewater into continuous electricity using surface-encapsulated polyelectrolyte-modified wood membranes. By exploiting wood's hierarchical porous structure, the authors fabricate anion- and cation-selective membranes through dip-coating and sunlight-induced polymerization, achieving a device composed of about*

98% bio-based materials. The study provides a low-cost, scalable, and sustainable route for coupling wastewater utilization with osmotic energy harvesting in circular water-energy systems."

Response: We are deeply grateful to Reviewer for the careful and thorough reading of our revised manuscript and response letter, and especially for the highest recommendation and the generous summary of our work. The reviewer's insightful comments throughout the review process have been invaluable in strengthening the quality and clarity of this manuscript. We are pleased that the revisions have fully addressed all concerns raised.

We hope that the revised manuscript, together with this point-by-point response, is now suitable for publication in Nature Communications. We remain available to address any further questions or requests from the editorial office.

Yours sincerely,

Kunkun Tu and Shihang Li

On behalf of all co-authors
